# Supplementary figures and images for: Projecting Future Climate Change-Mediated Impacts in Three Paralytic Shellfish Toxins-Producing Dinoflagellate Species
Source: Biology (Basel). 2022 Sep 29;11(10):1424. doi: 10.3390/biology11101424 (PMC9598431; doi:10.3390/biology11101424)

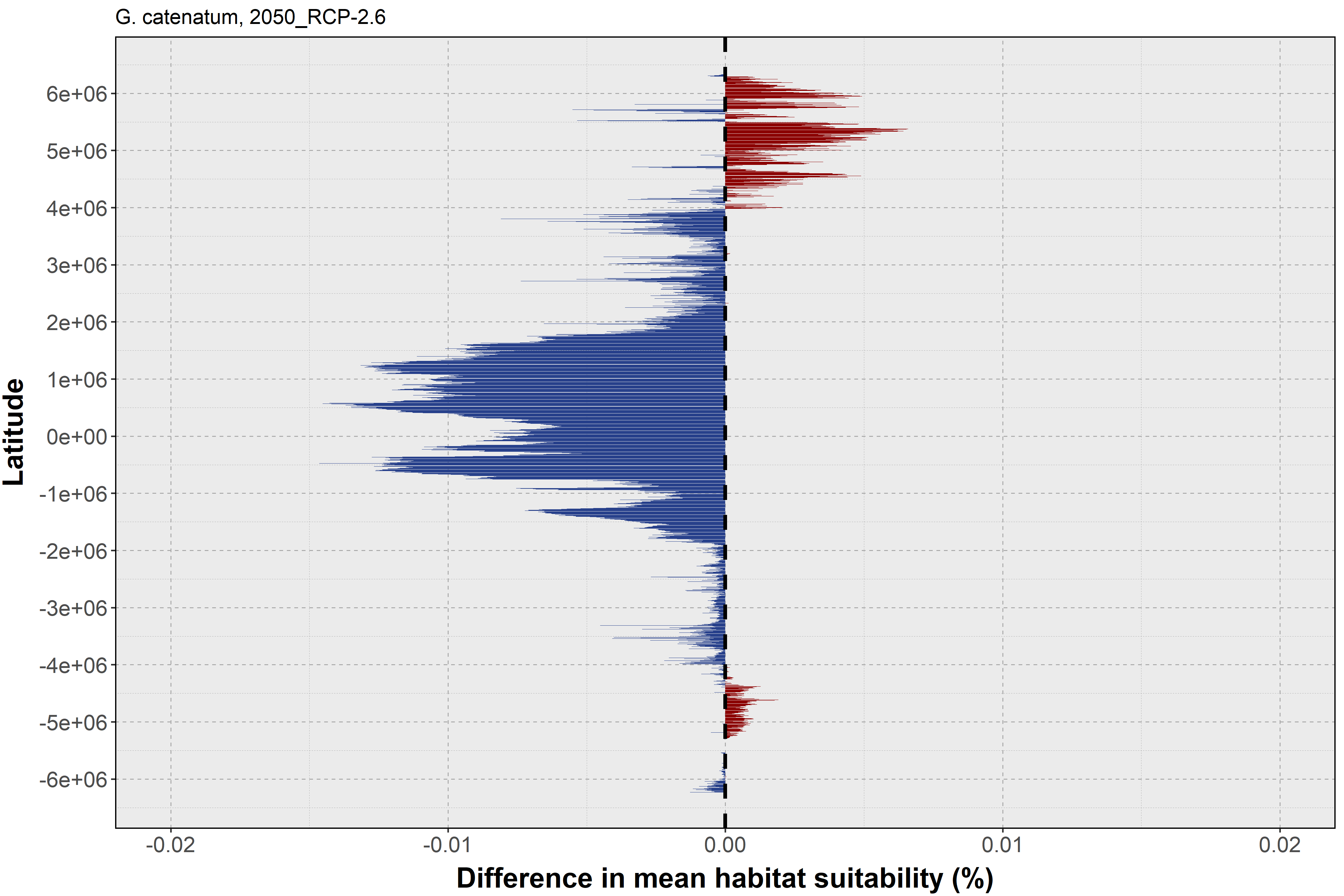

Supplement: Supplementary file 1 [file biology-11-01424-s001.zip › High_Res_Figures/catenatum_habdif205026.tiff]

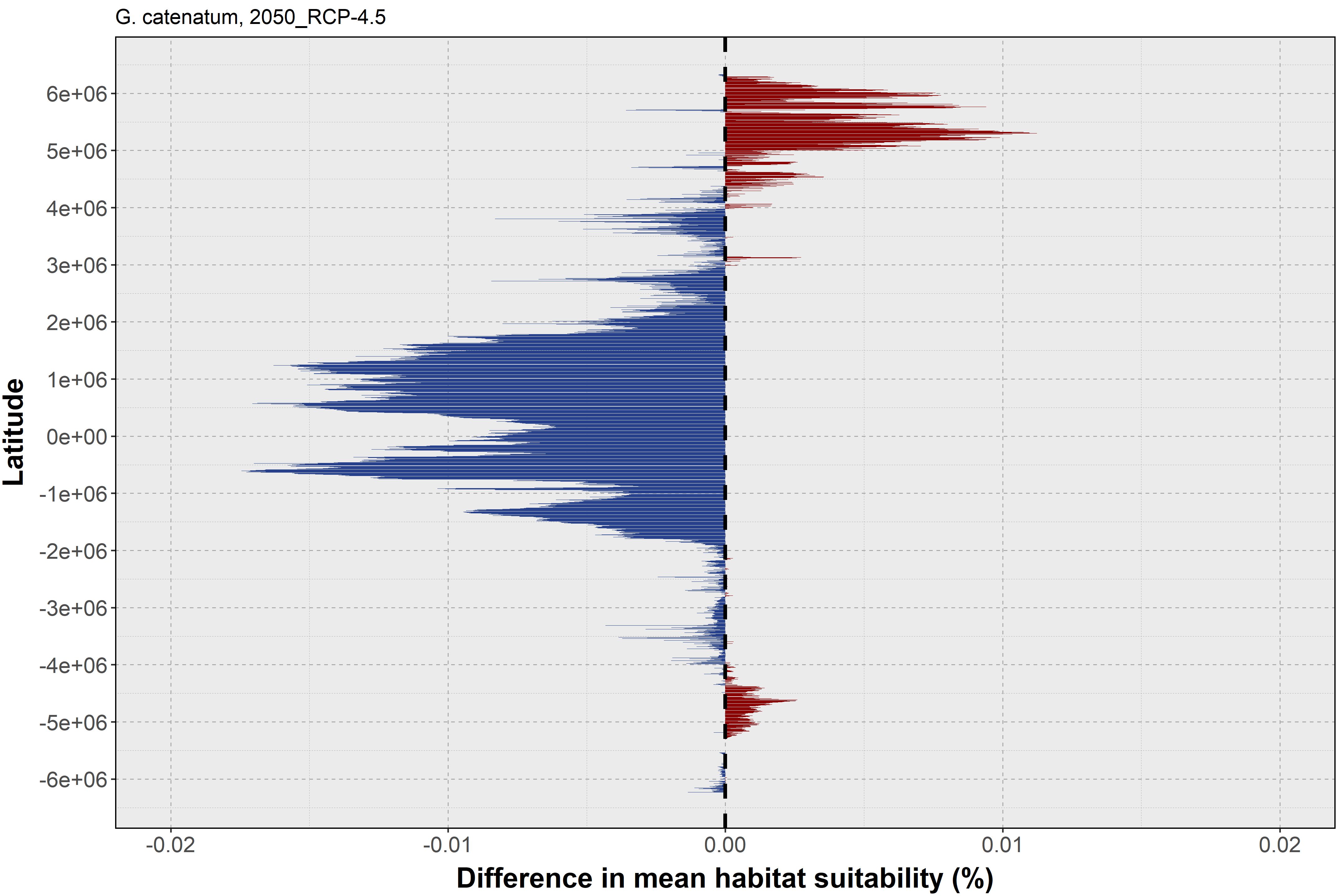

Supplement: Supplementary file 1 [file biology-11-01424-s001.zip › High_Res_Figures/catenatum_habdif205045.tiff]

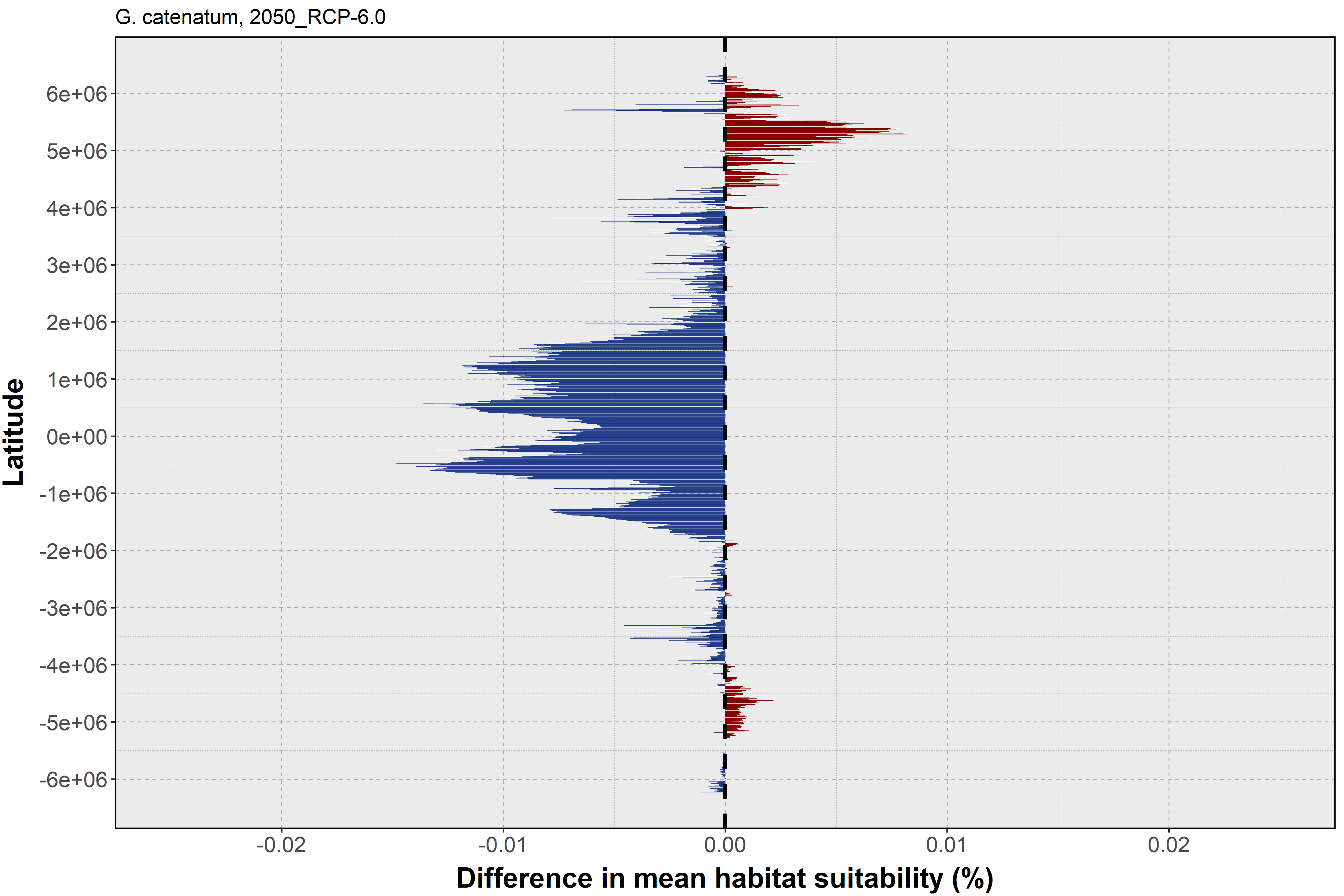

Supplement: Supplementary file 1 [file biology-11-01424-s001.zip › High_Res_Figures/catenatum_habdif205060.tiff]

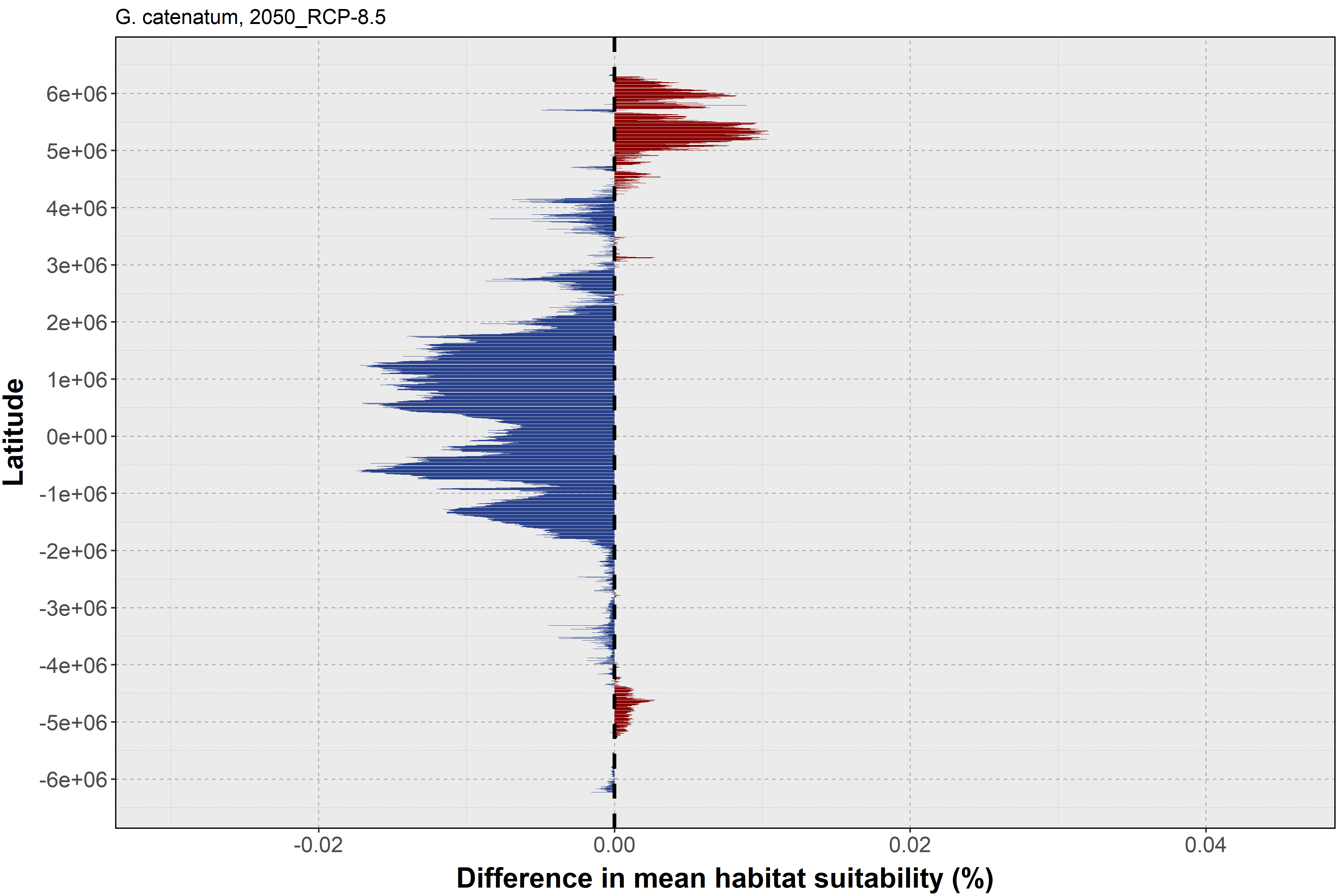

Supplement: Supplementary file 1 [file biology-11-01424-s001.zip › High_Res_Figures/catenatum_habdif205085.tiff]

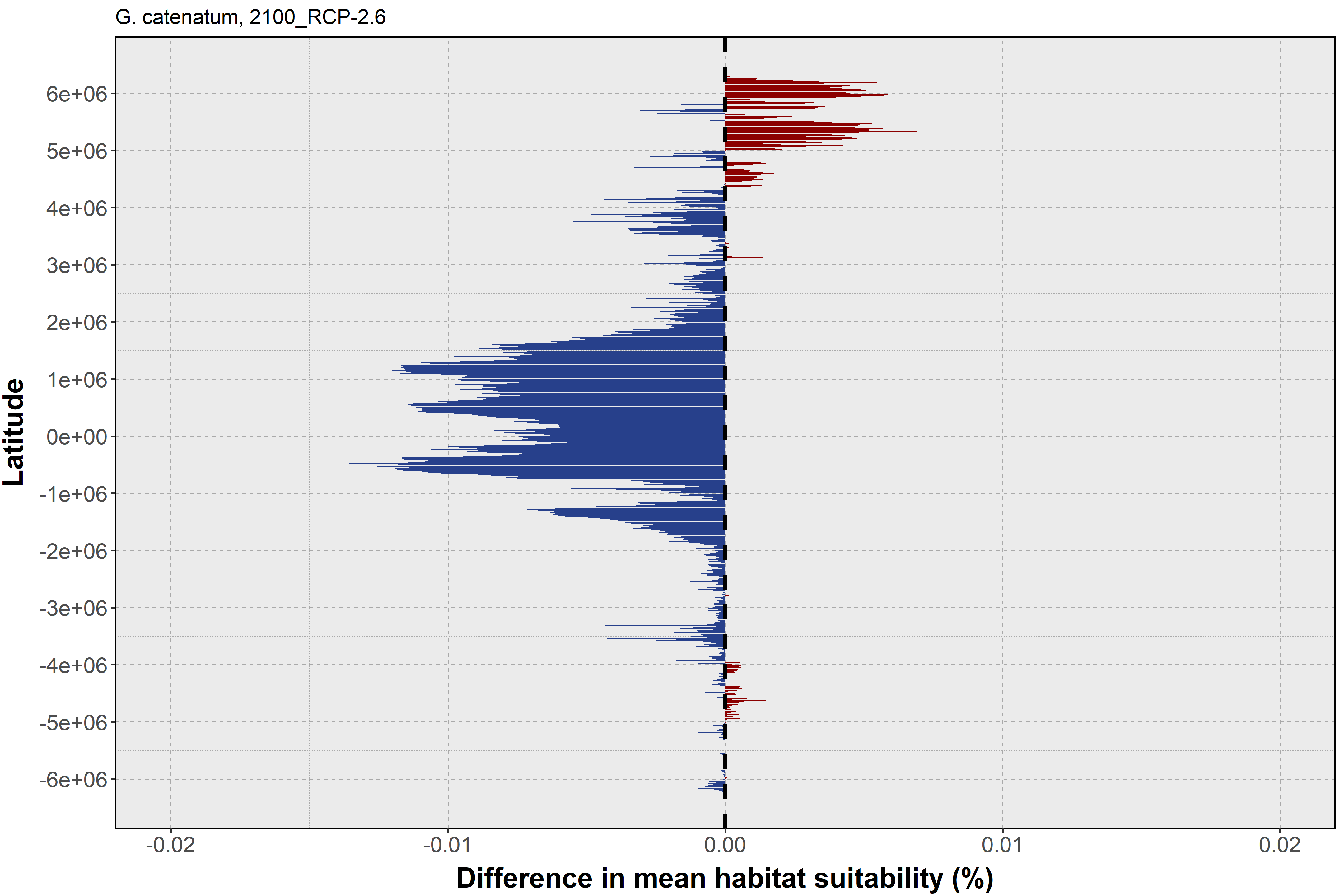

Supplement: Supplementary file 1 [file biology-11-01424-s001.zip › High_Res_Figures/catenatum_habdif210026.tiff]

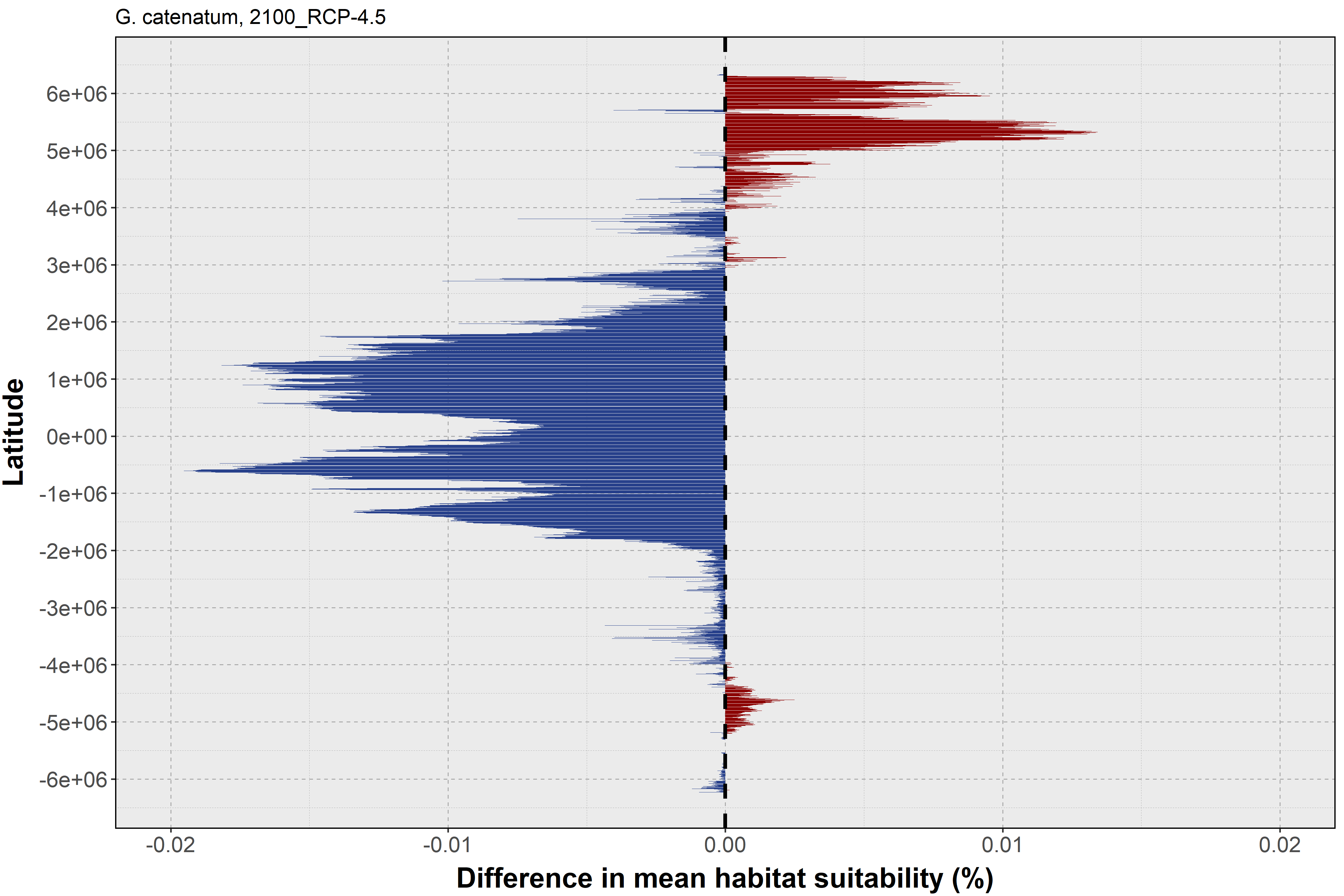

Supplement: Supplementary file 1 [file biology-11-01424-s001.zip › High_Res_Figures/catenatum_habdif210045.tiff]

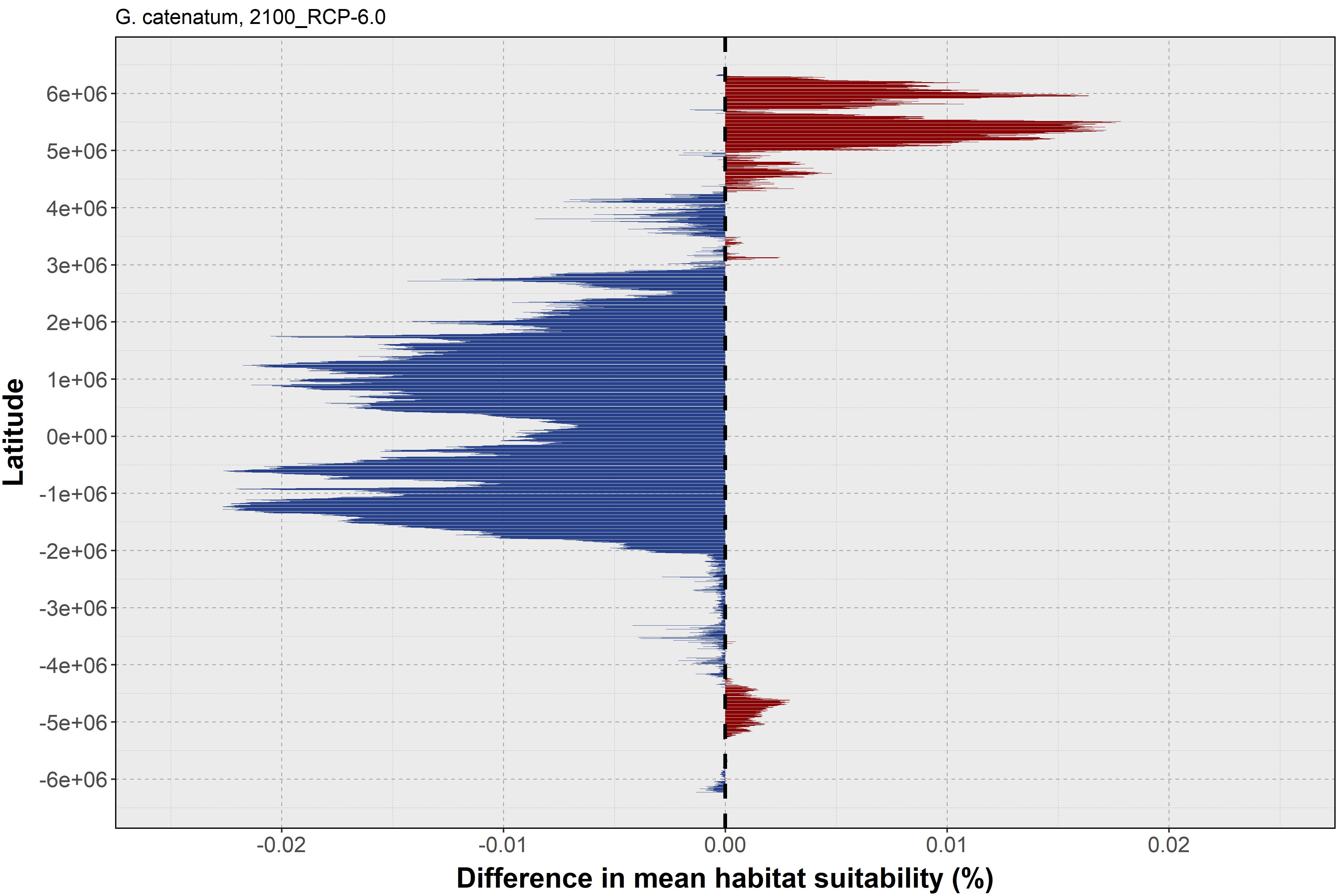

Supplement: Supplementary file 1 [file biology-11-01424-s001.zip › High_Res_Figures/catenatum_habdif210060.tiff]

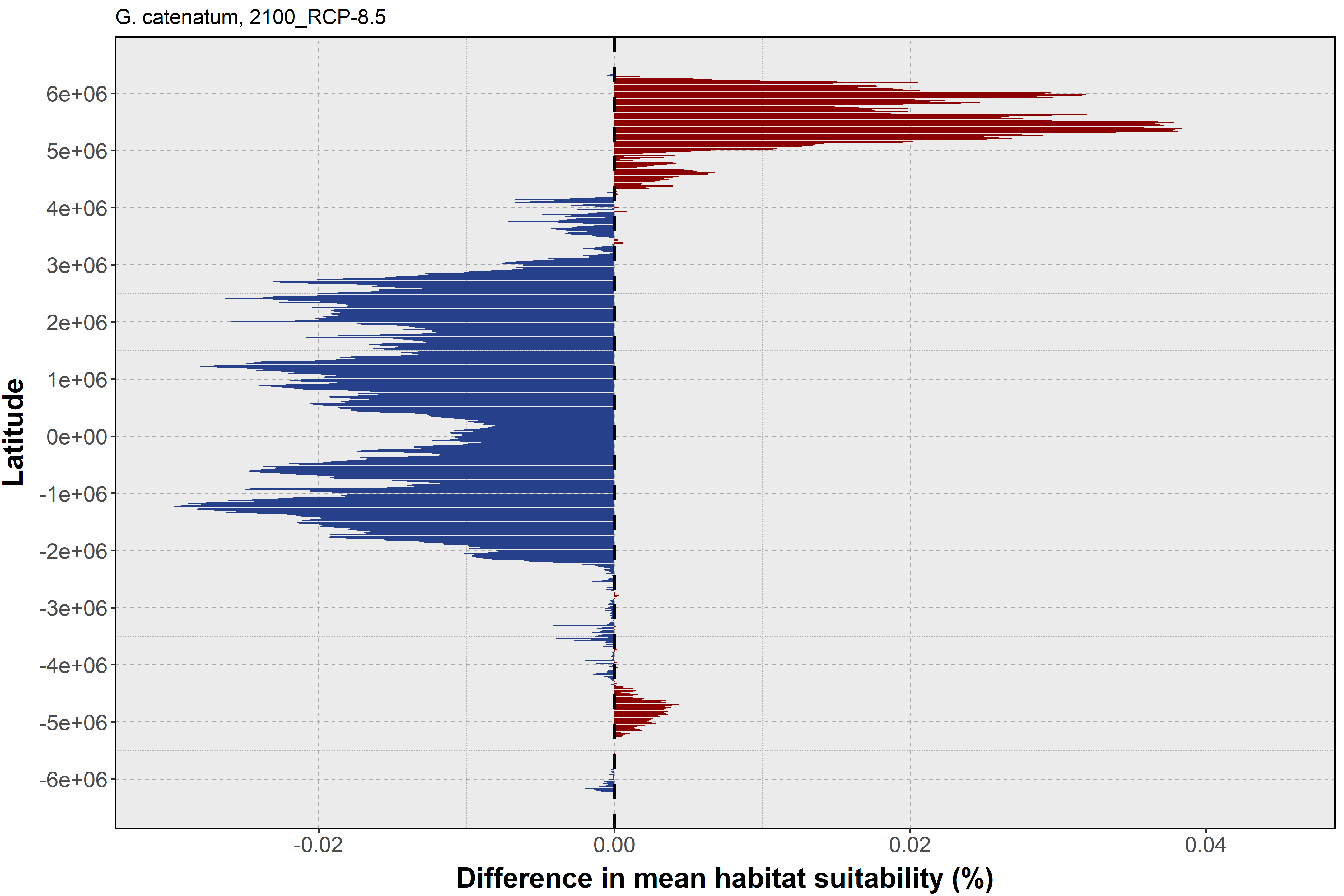

Supplement: Supplementary file 1 [file biology-11-01424-s001.zip › High_Res_Figures/catenatum_habdif210085.tiff]

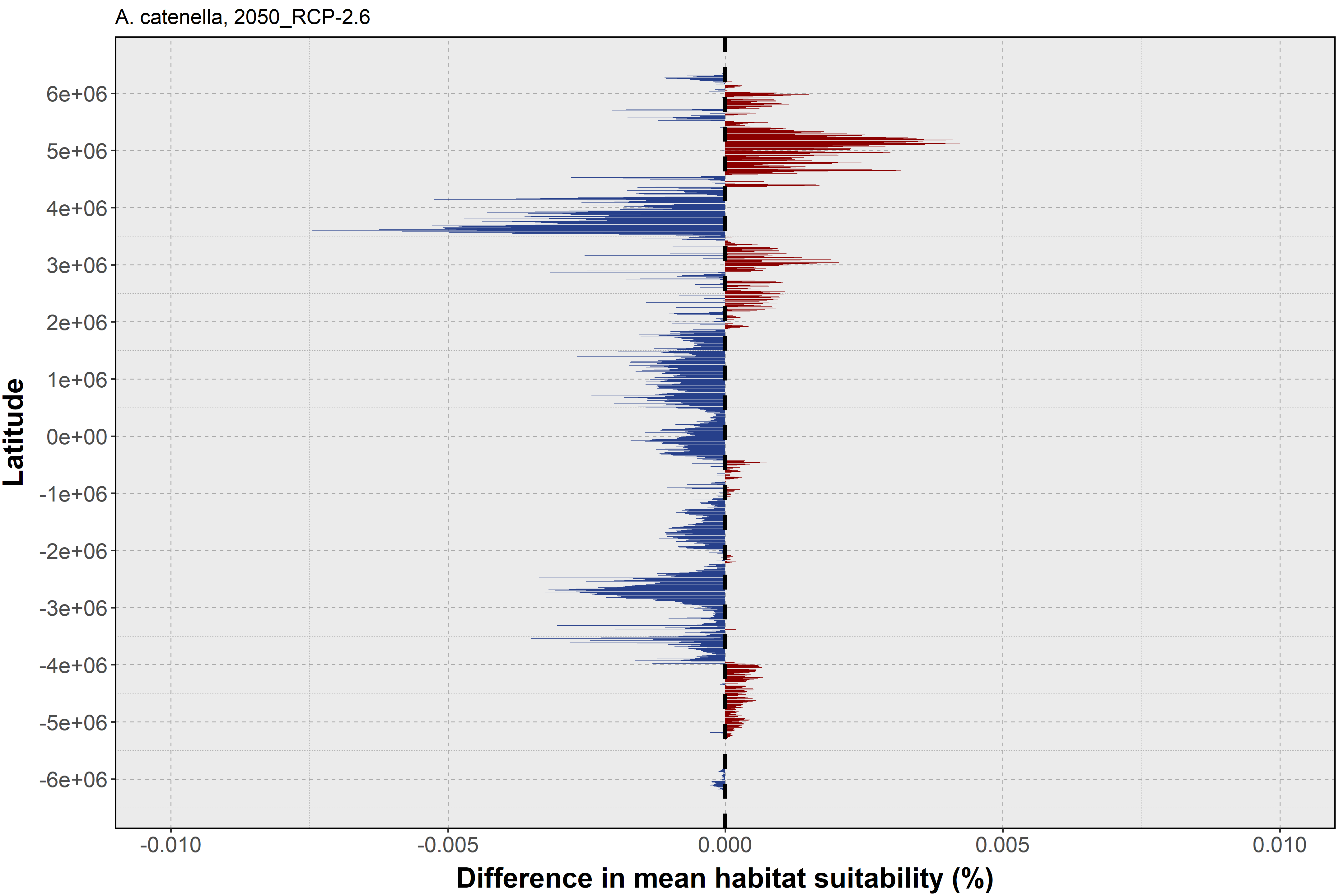

Supplement: Supplementary file 1 [file biology-11-01424-s001.zip › High_Res_Figures/catenella_habdif205026.tiff]

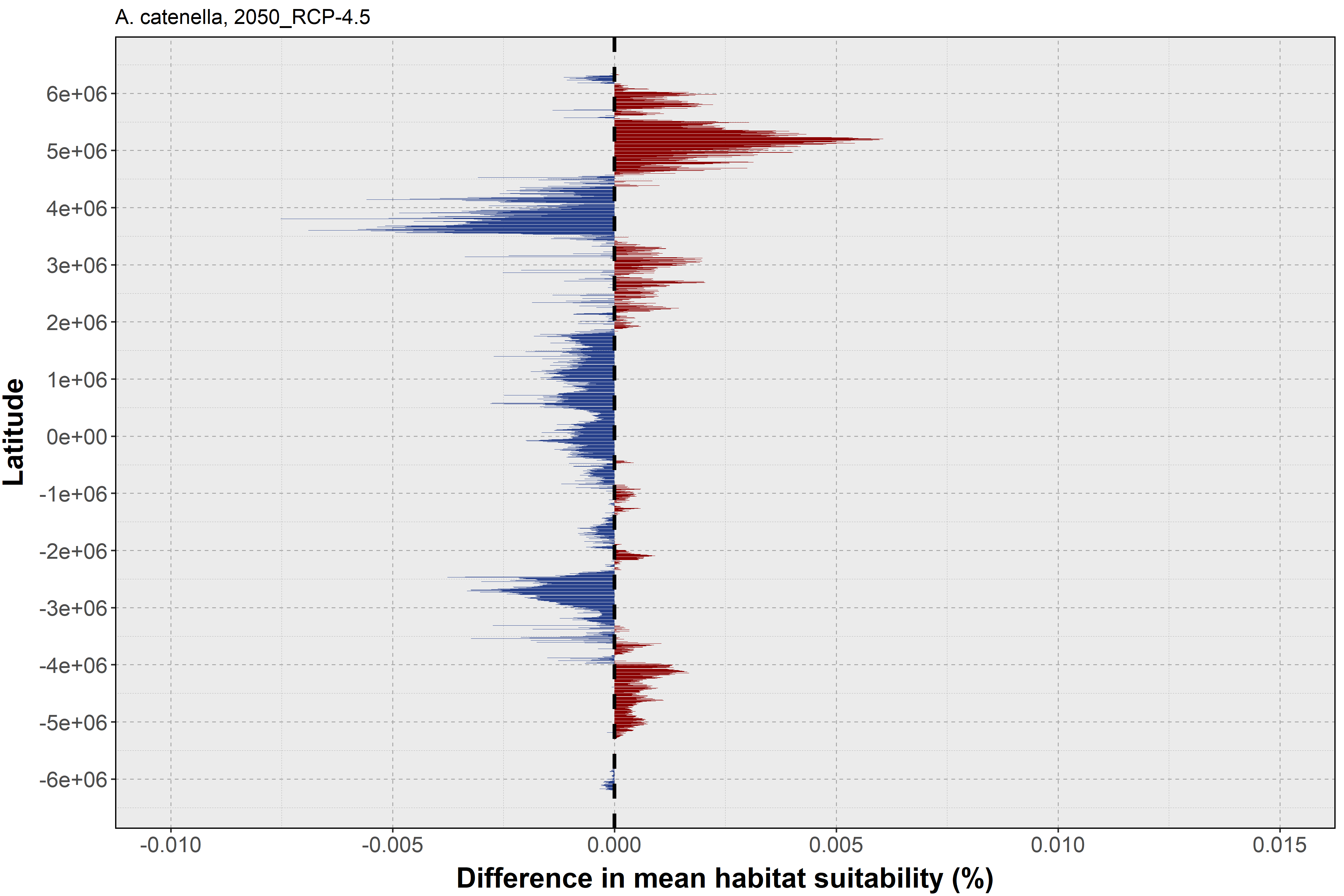

Supplement: Supplementary file 1 [file biology-11-01424-s001.zip › High_Res_Figures/catenella_habdif205045.tiff]

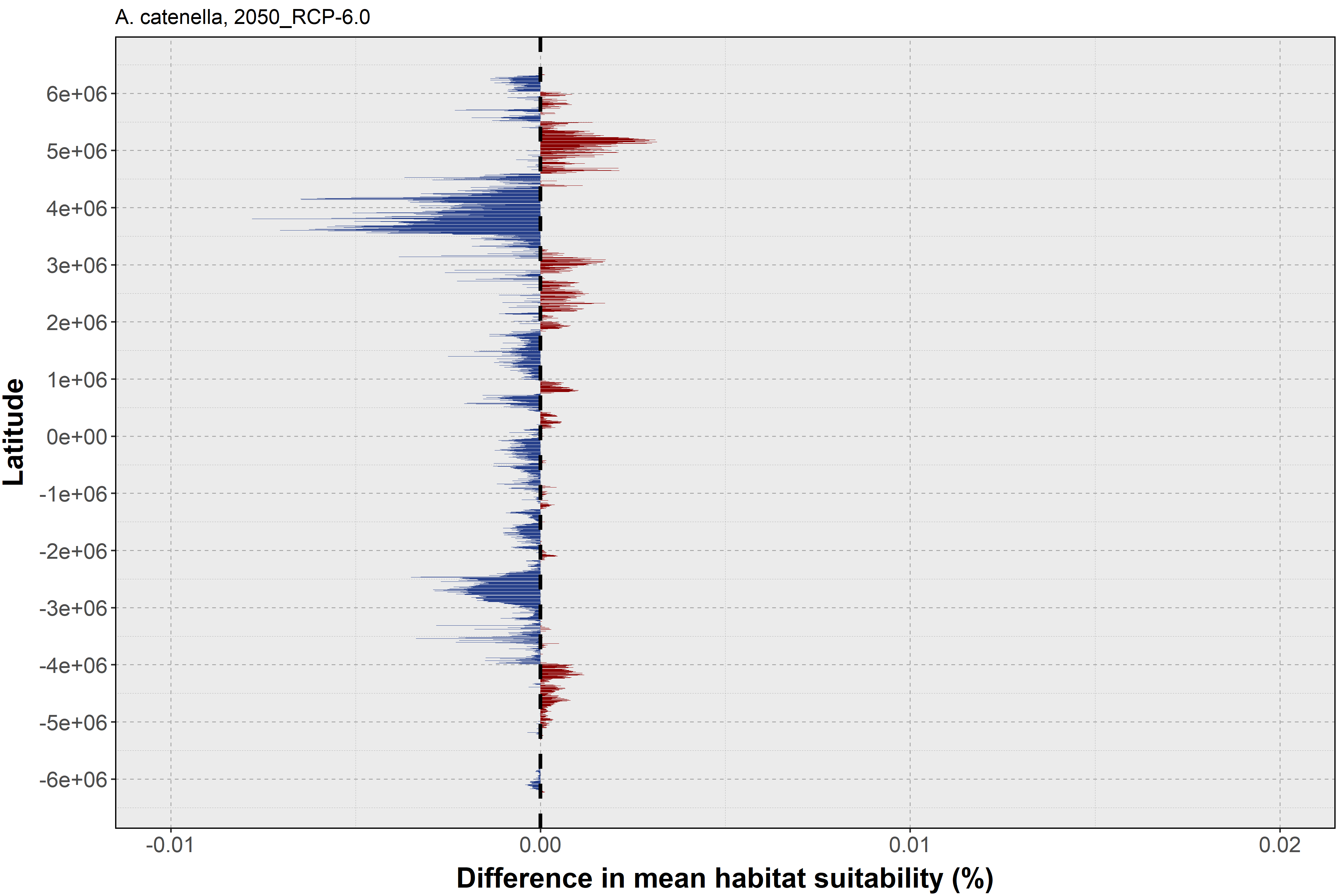

Supplement: Supplementary file 1 [file biology-11-01424-s001.zip › High_Res_Figures/catenella_habdif205060.tiff]

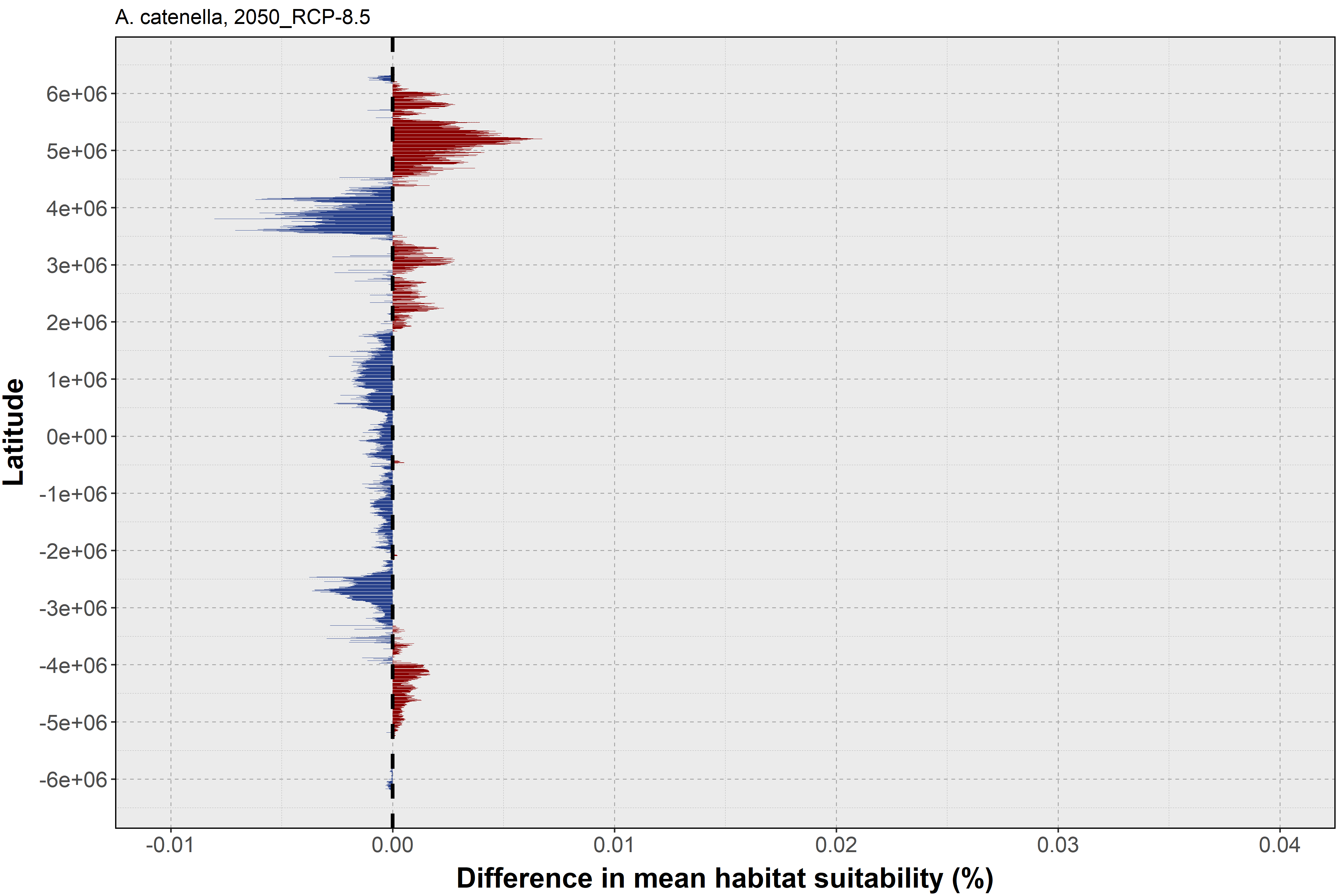

Supplement: Supplementary file 1 [file biology-11-01424-s001.zip › High_Res_Figures/catenella_habdif205085.tiff]

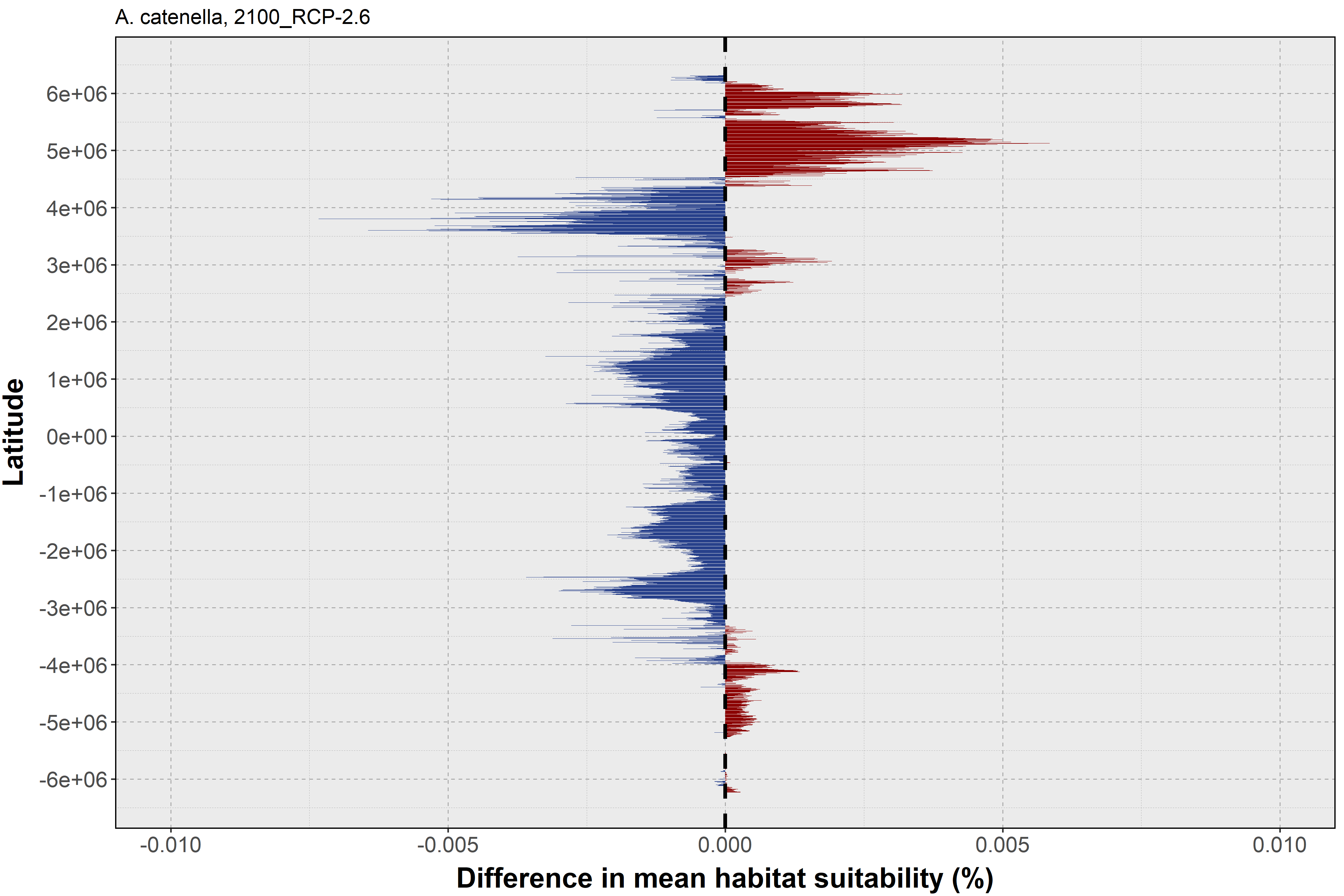

Supplement: Supplementary file 1 [file biology-11-01424-s001.zip › High_Res_Figures/catenella_habdif210026.tiff]

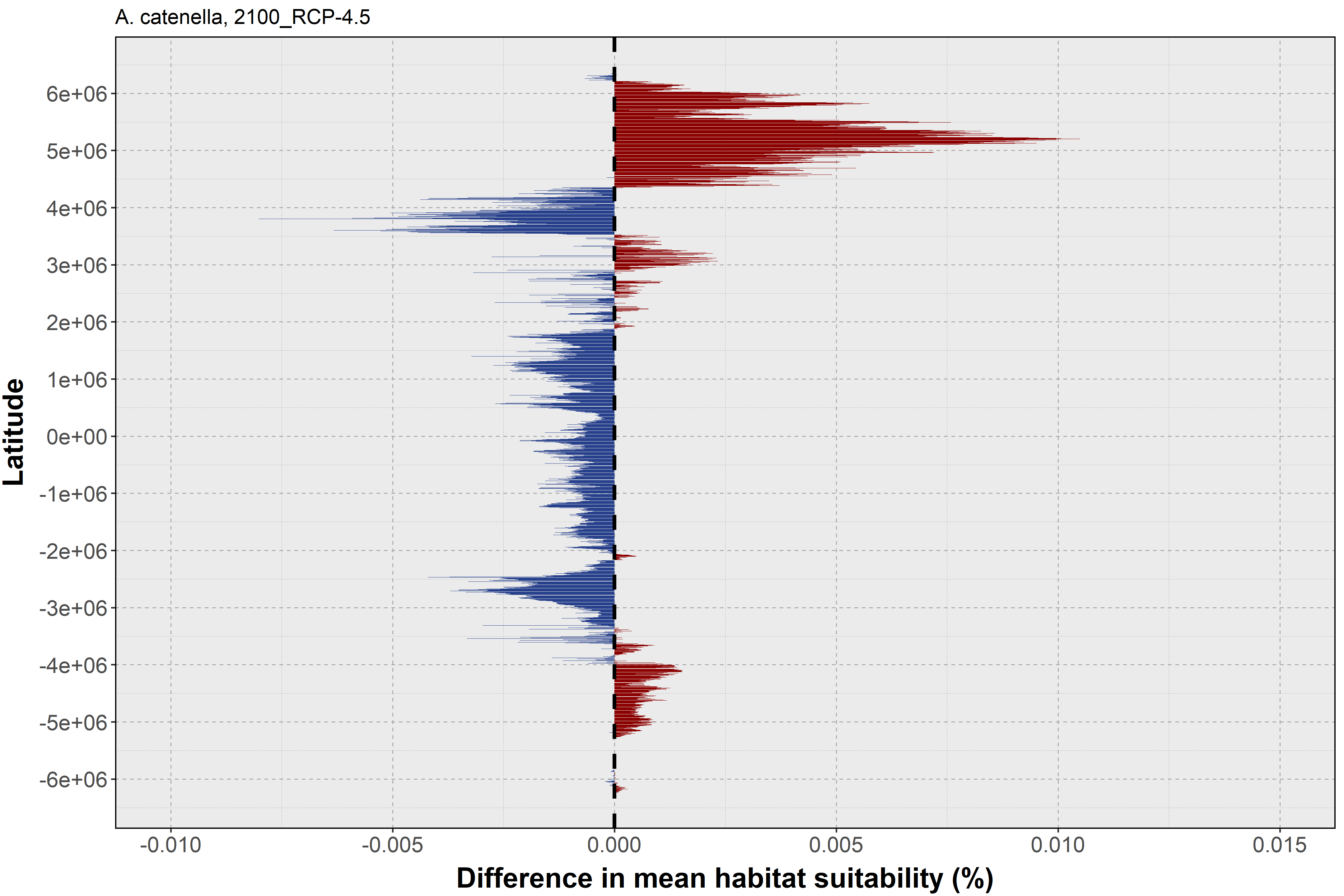

Supplement: Supplementary file 1 [file biology-11-01424-s001.zip › High_Res_Figures/catenella_habdif210045.tiff]

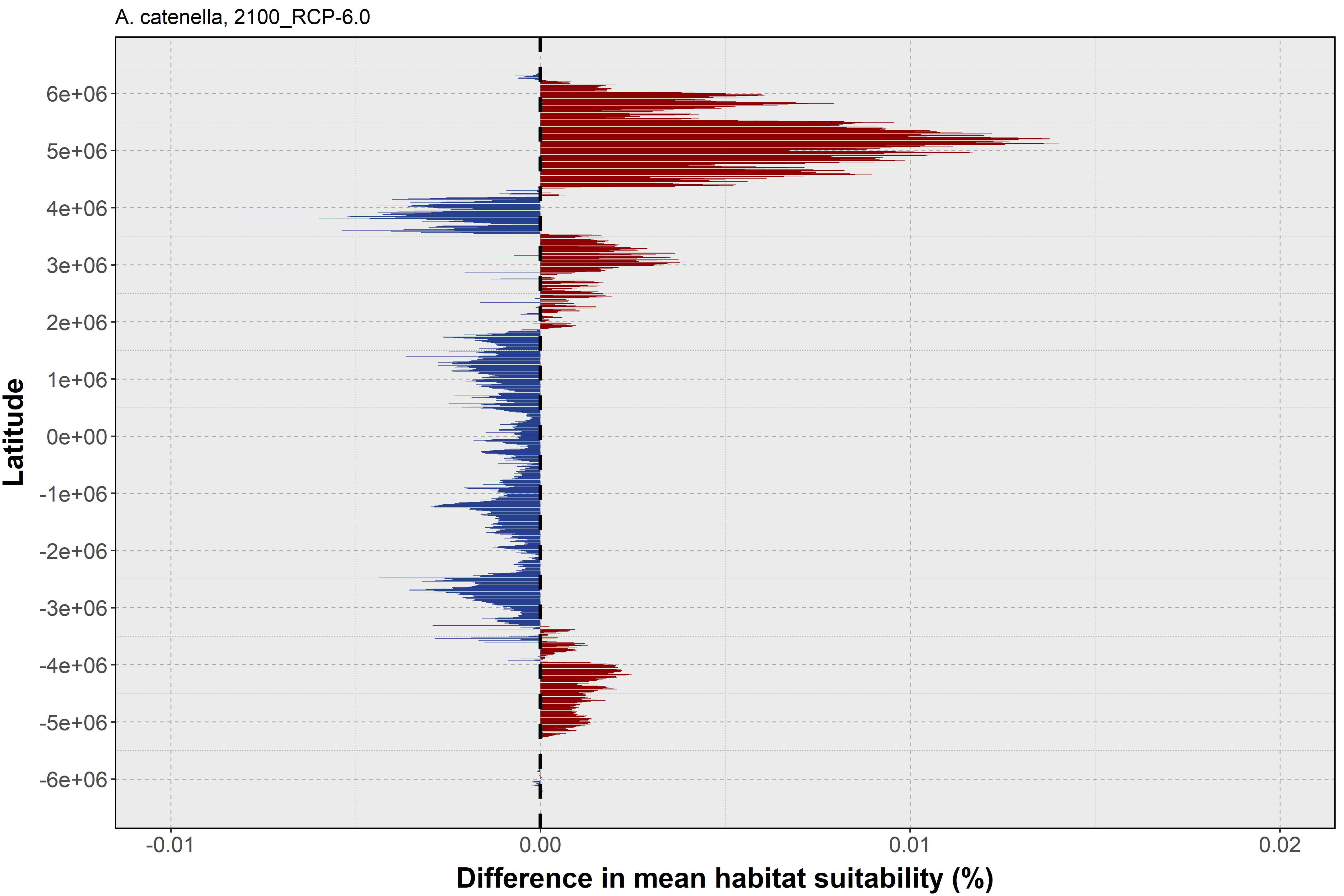

Supplement: Supplementary file 1 [file biology-11-01424-s001.zip › High_Res_Figures/catenella_habdif210060.tiff]

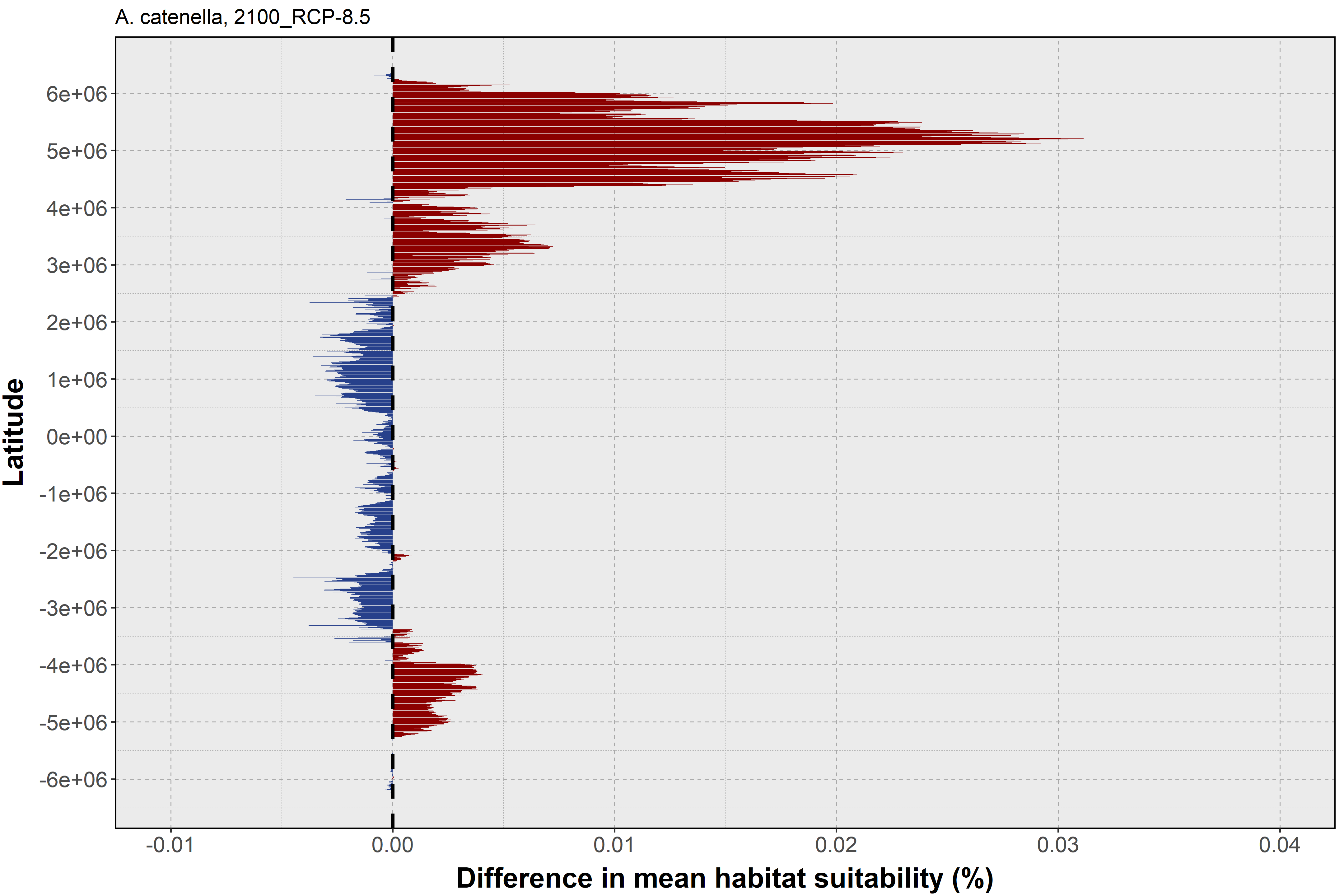

Supplement: Supplementary file 1 [file biology-11-01424-s001.zip › High_Res_Figures/catenella_habdif210085.tiff]

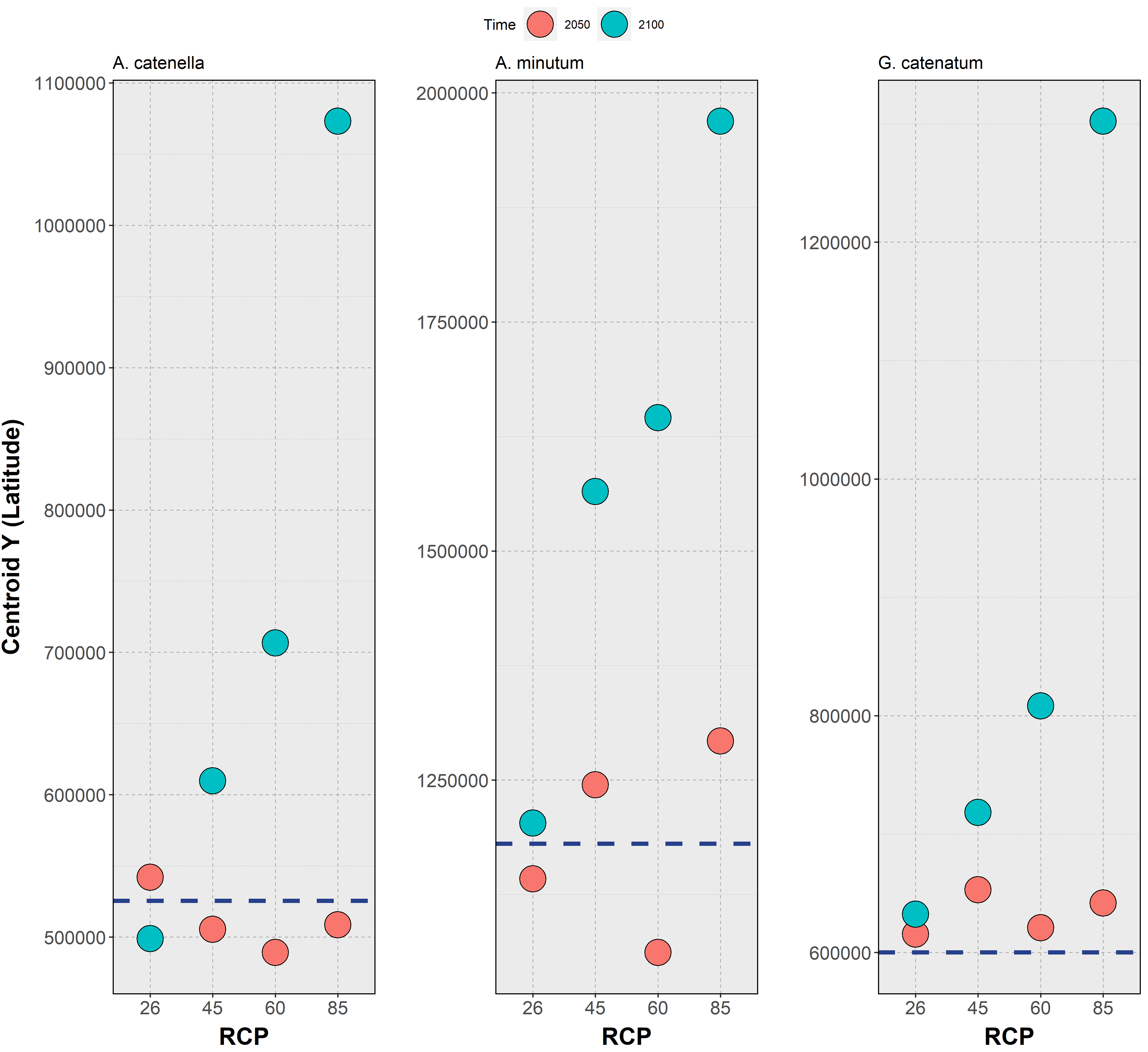

Supplement: Supplementary file 1 [file biology-11-01424-s001.zip › High_Res_Figures/centroids.tiff]

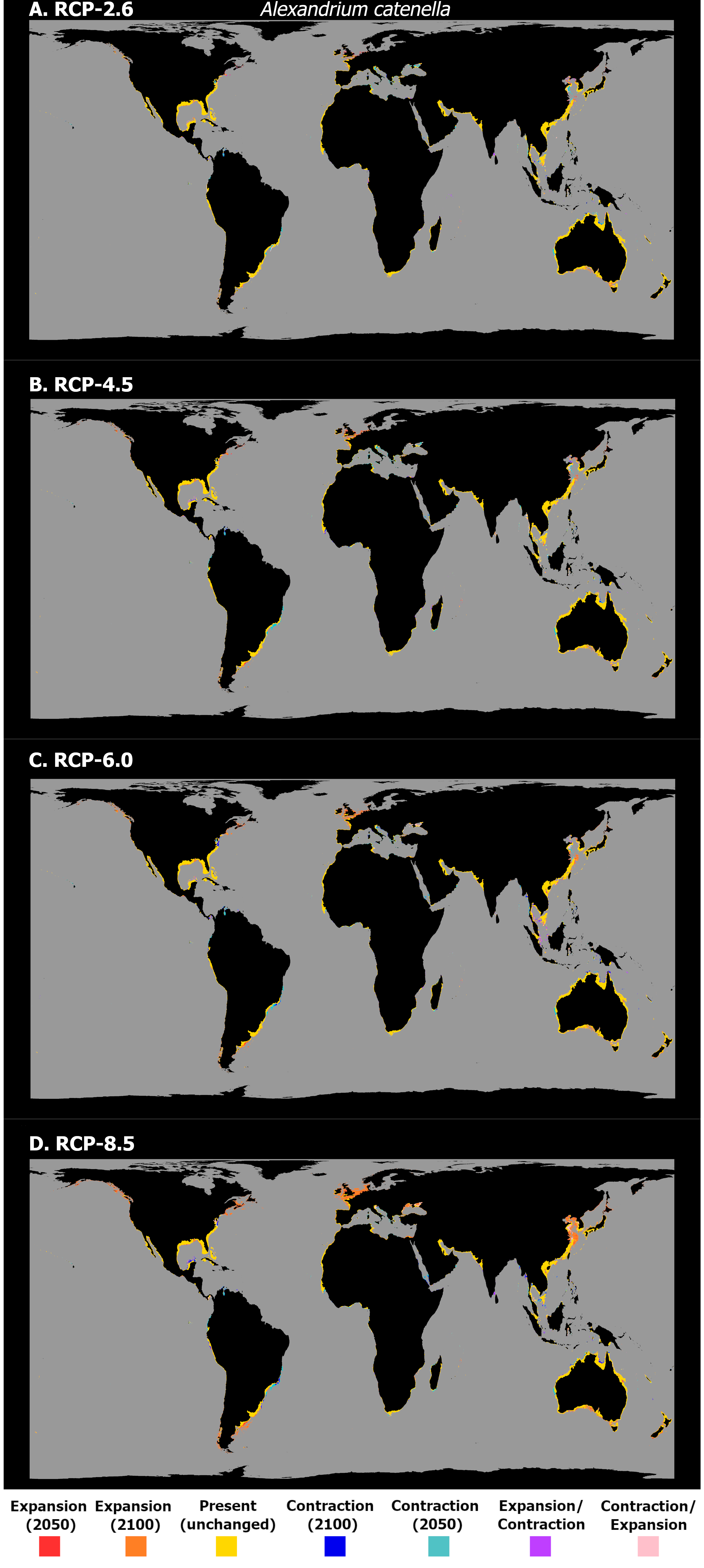

Supplement: Supplementary file 1 [file biology-11-01424-s001.zip › High_Res_Figures/Figure3_new.png]

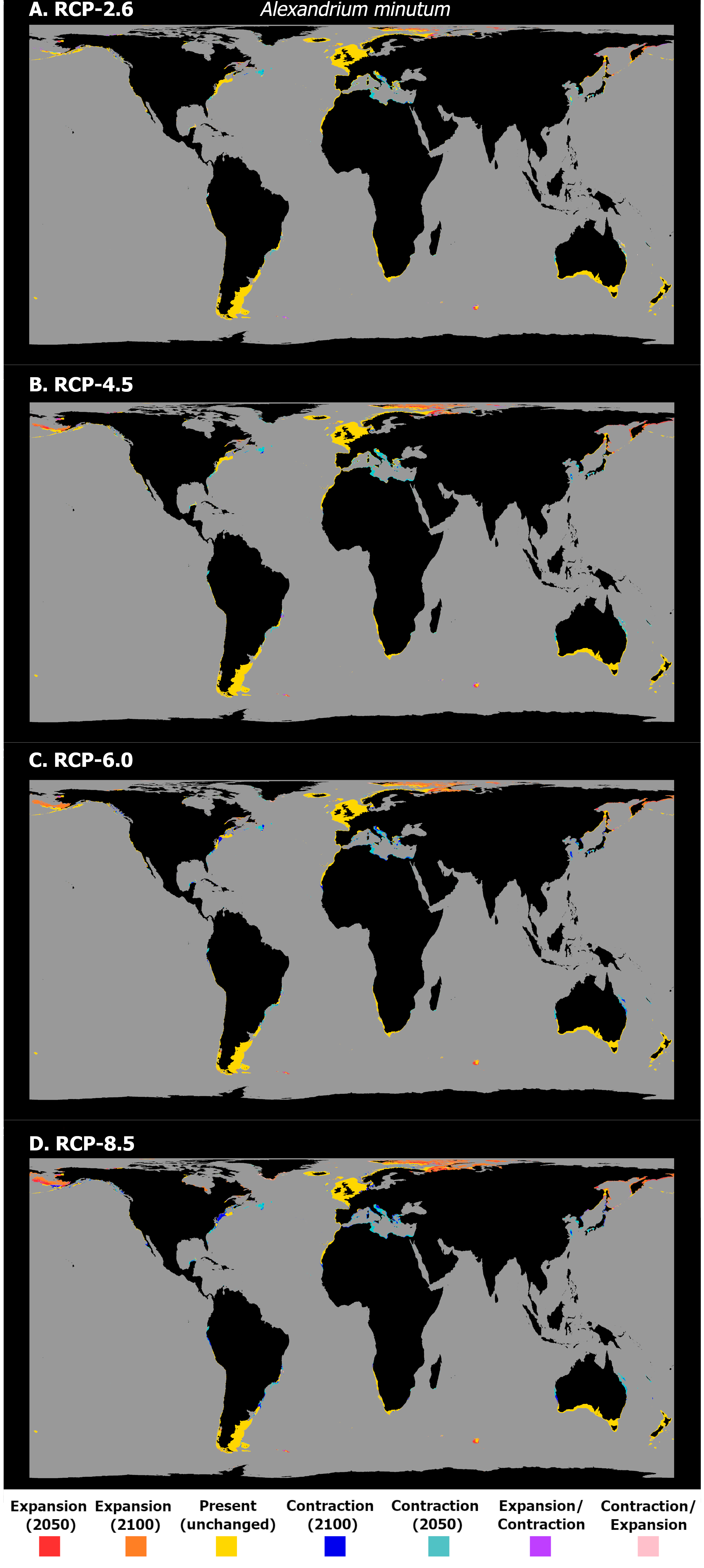

Supplement: Supplementary file 1 [file biology-11-01424-s001.zip › High_Res_Figures/Figure5_new.png]

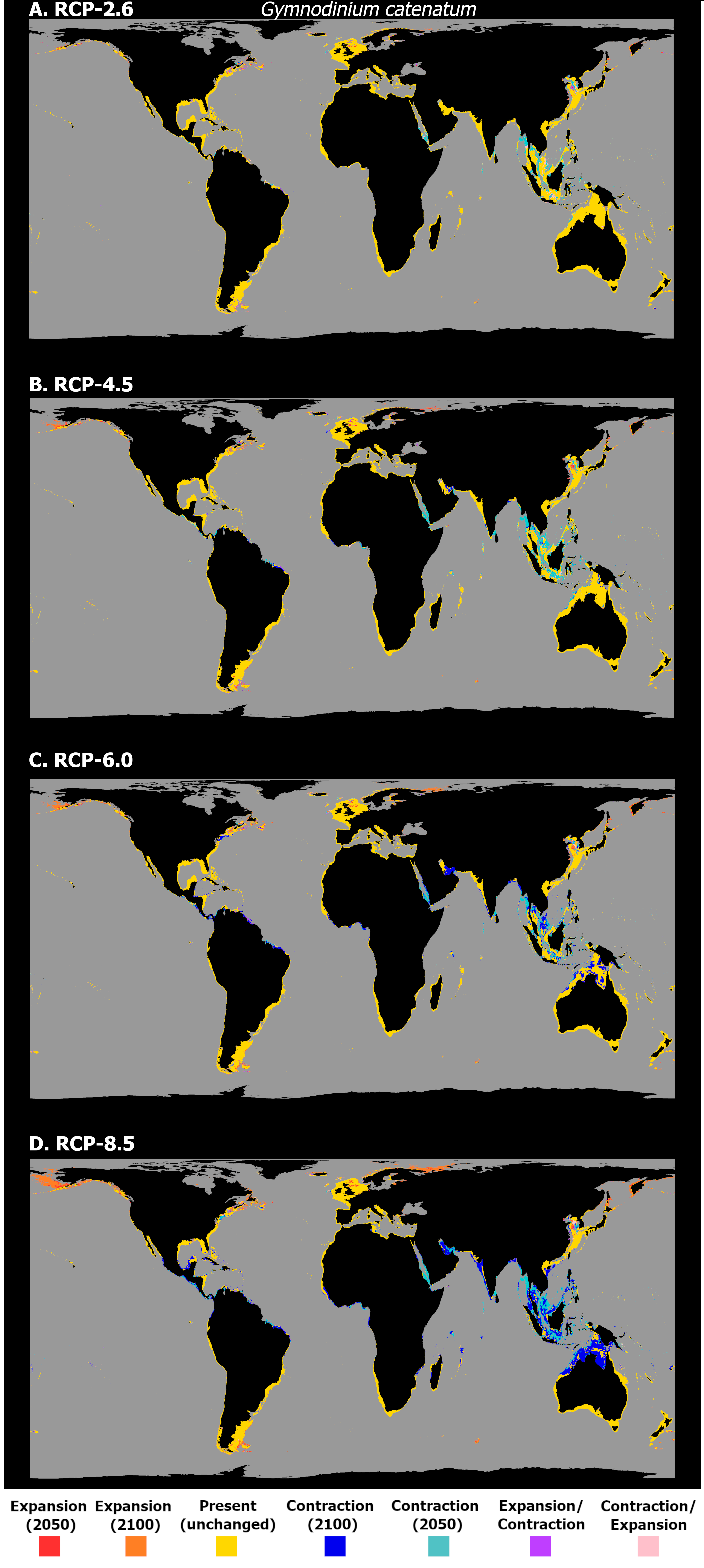

Supplement: Supplementary file 1 [file biology-11-01424-s001.zip › High_Res_Figures/Figure6_new.png]

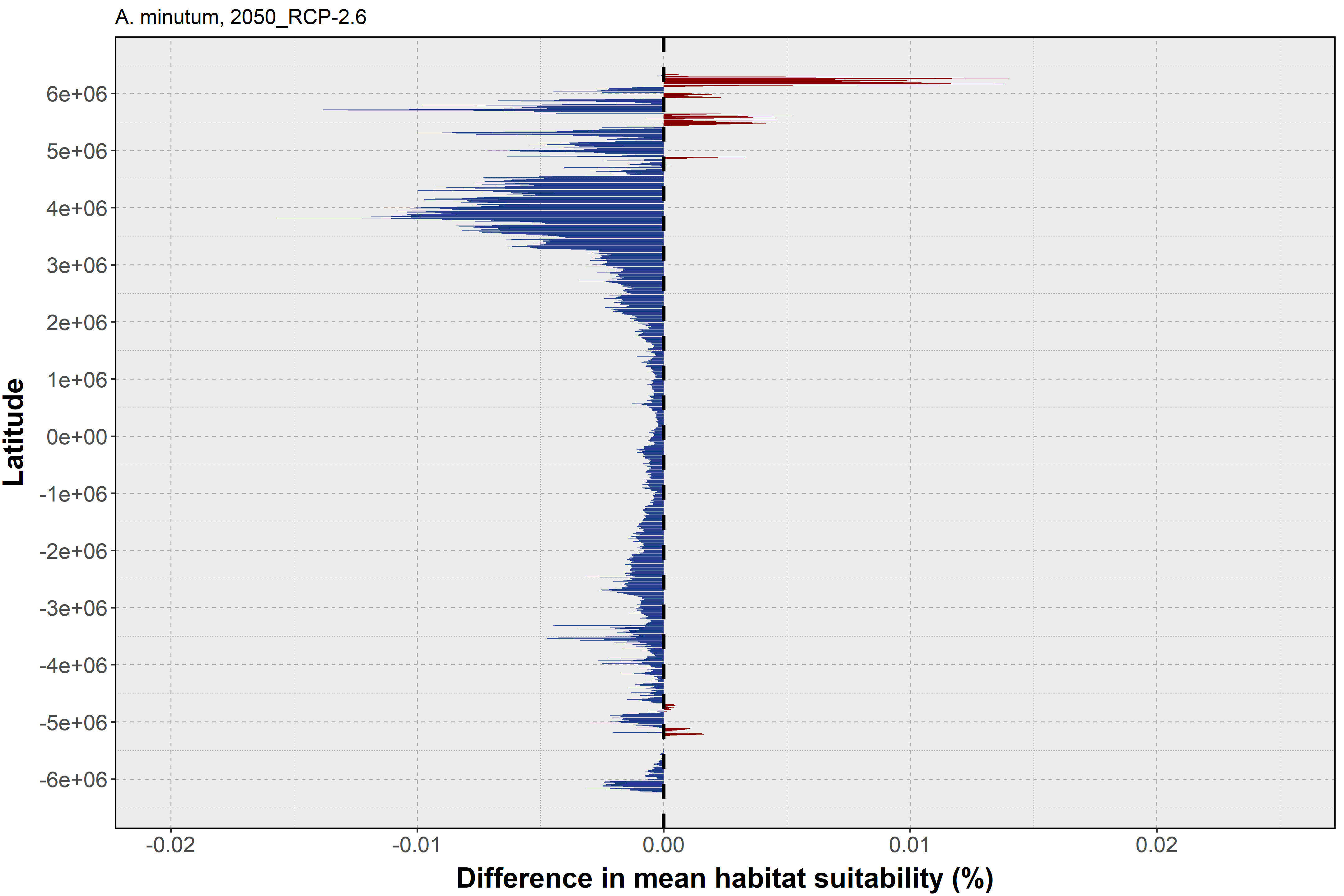

Supplement: Supplementary file 1 [file biology-11-01424-s001.zip › High_Res_Figures/minutum_habdif205026.tiff]

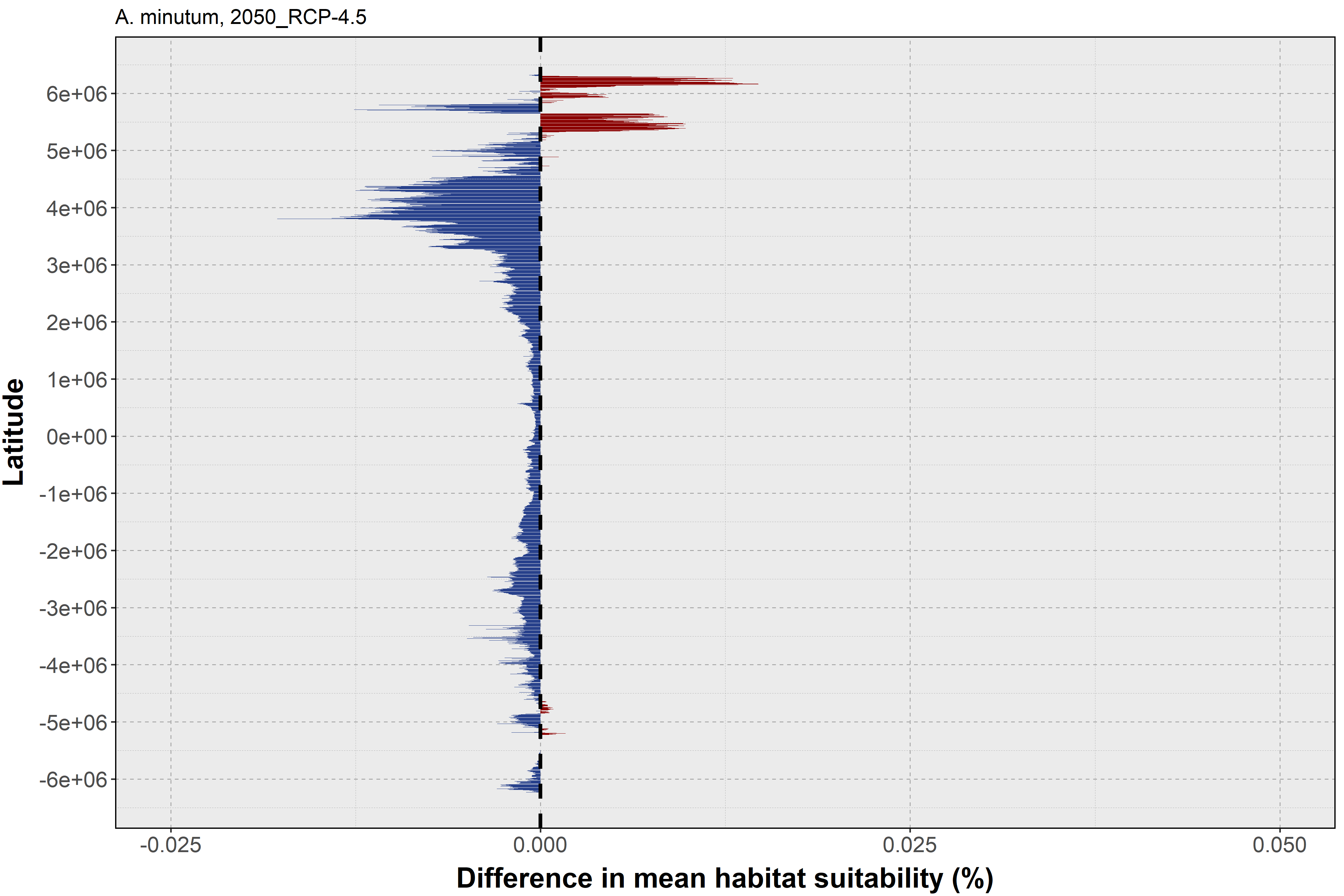

Supplement: Supplementary file 1 [file biology-11-01424-s001.zip › High_Res_Figures/minutum_habdif205045.tiff]

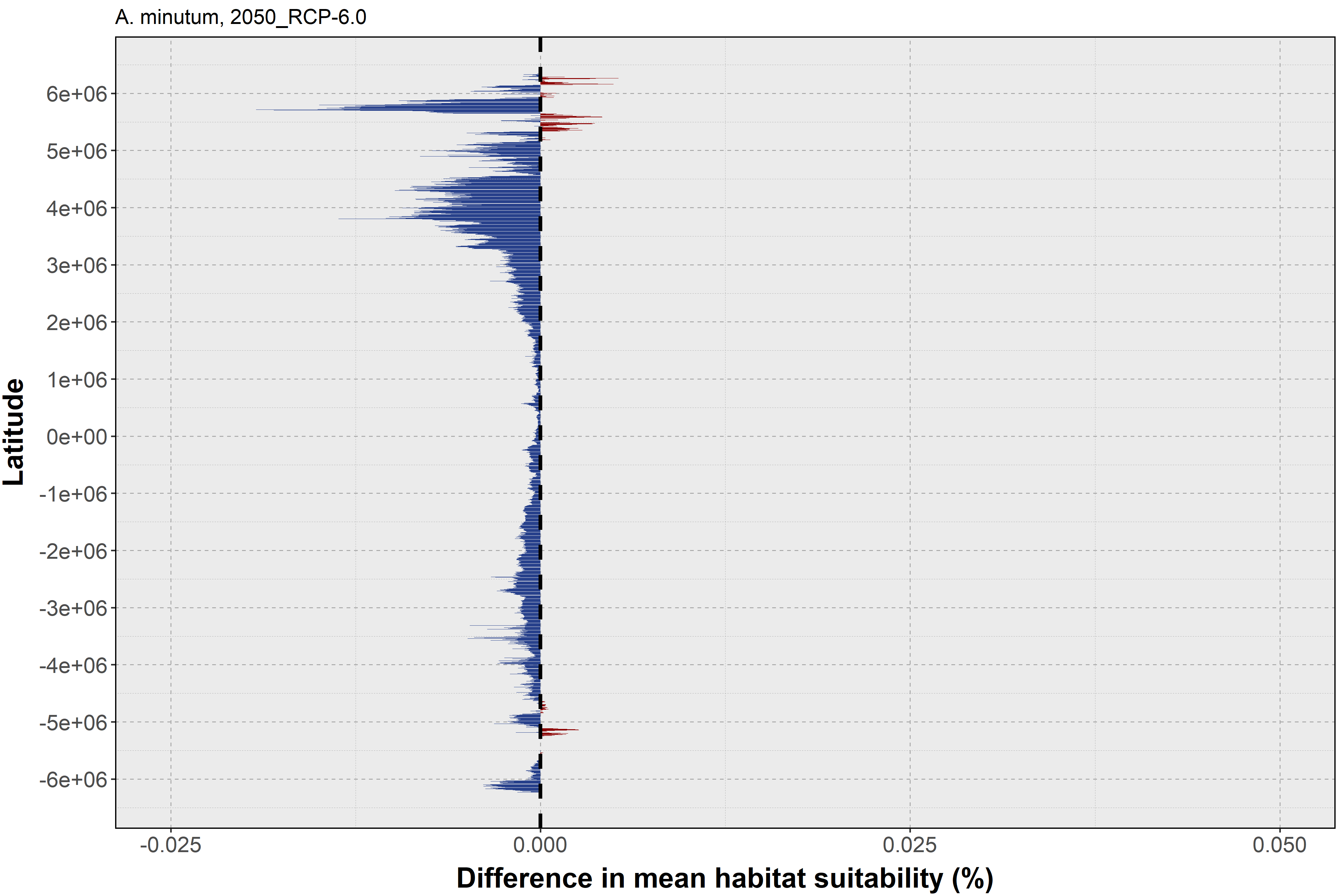

Supplement: Supplementary file 1 [file biology-11-01424-s001.zip › High_Res_Figures/minutum_habdif205060.tiff]

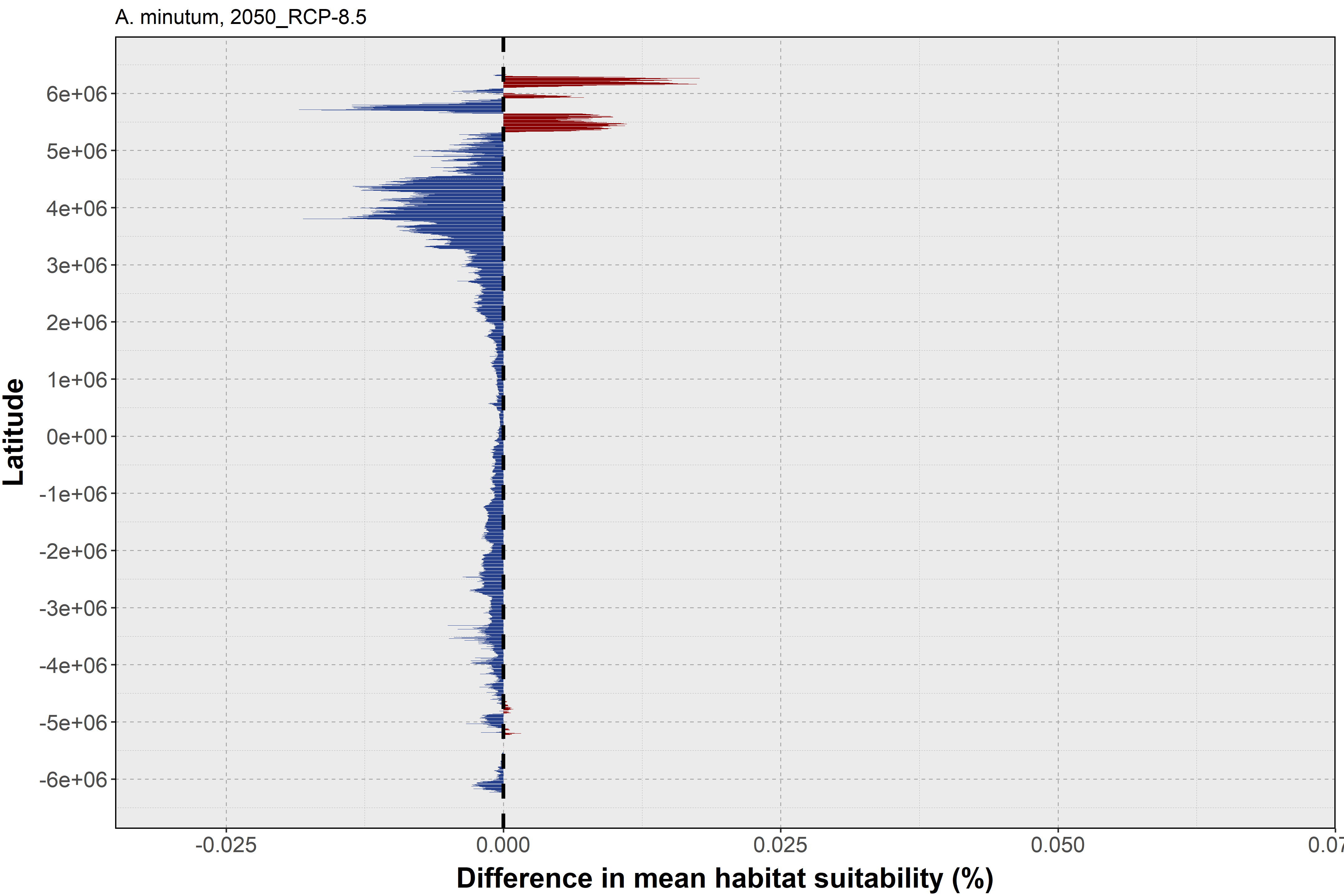

Supplement: Supplementary file 1 [file biology-11-01424-s001.zip › High_Res_Figures/minutum_habdif205085.tiff]

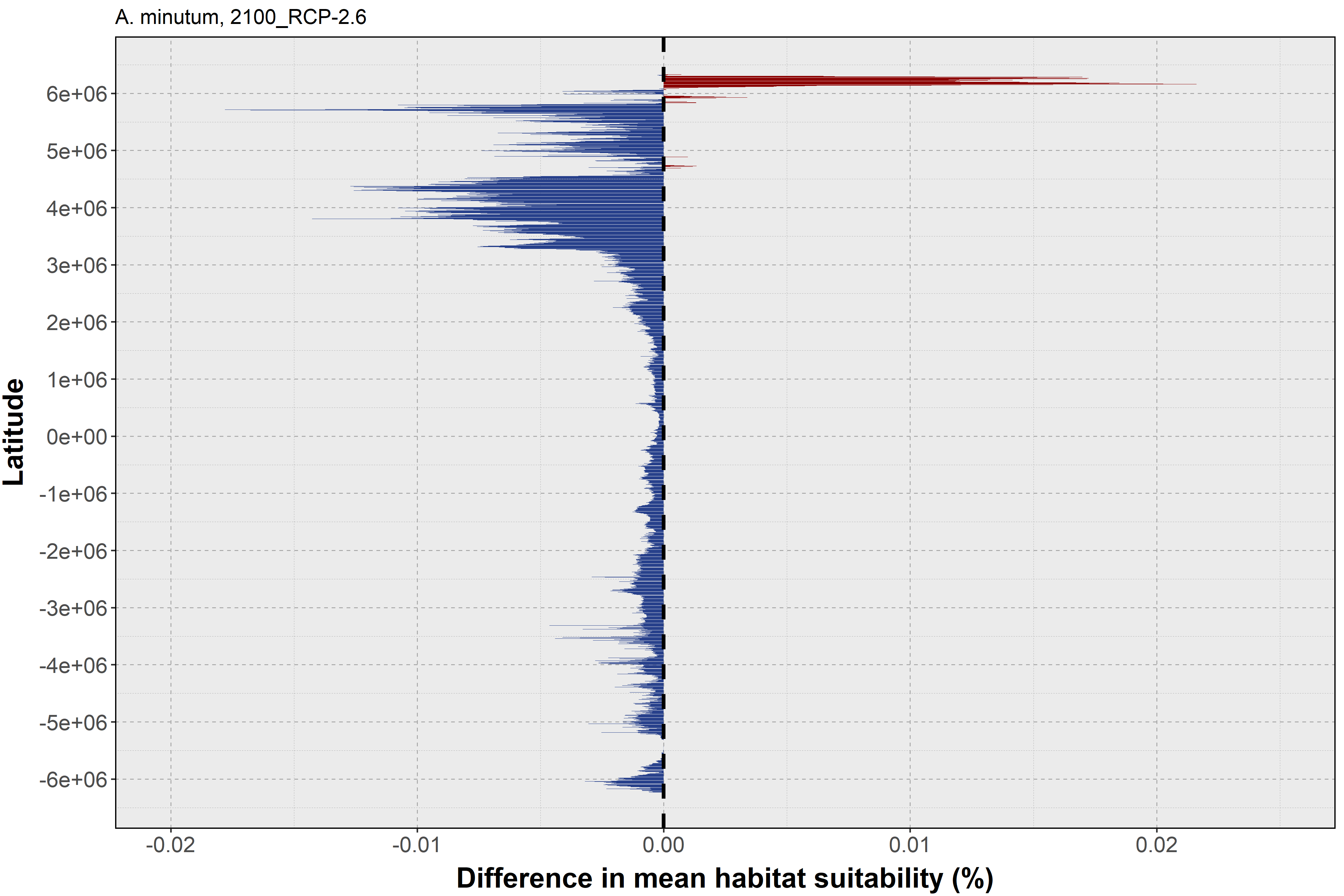

Supplement: Supplementary file 1 [file biology-11-01424-s001.zip › High_Res_Figures/minutum_habdif210026.tiff]

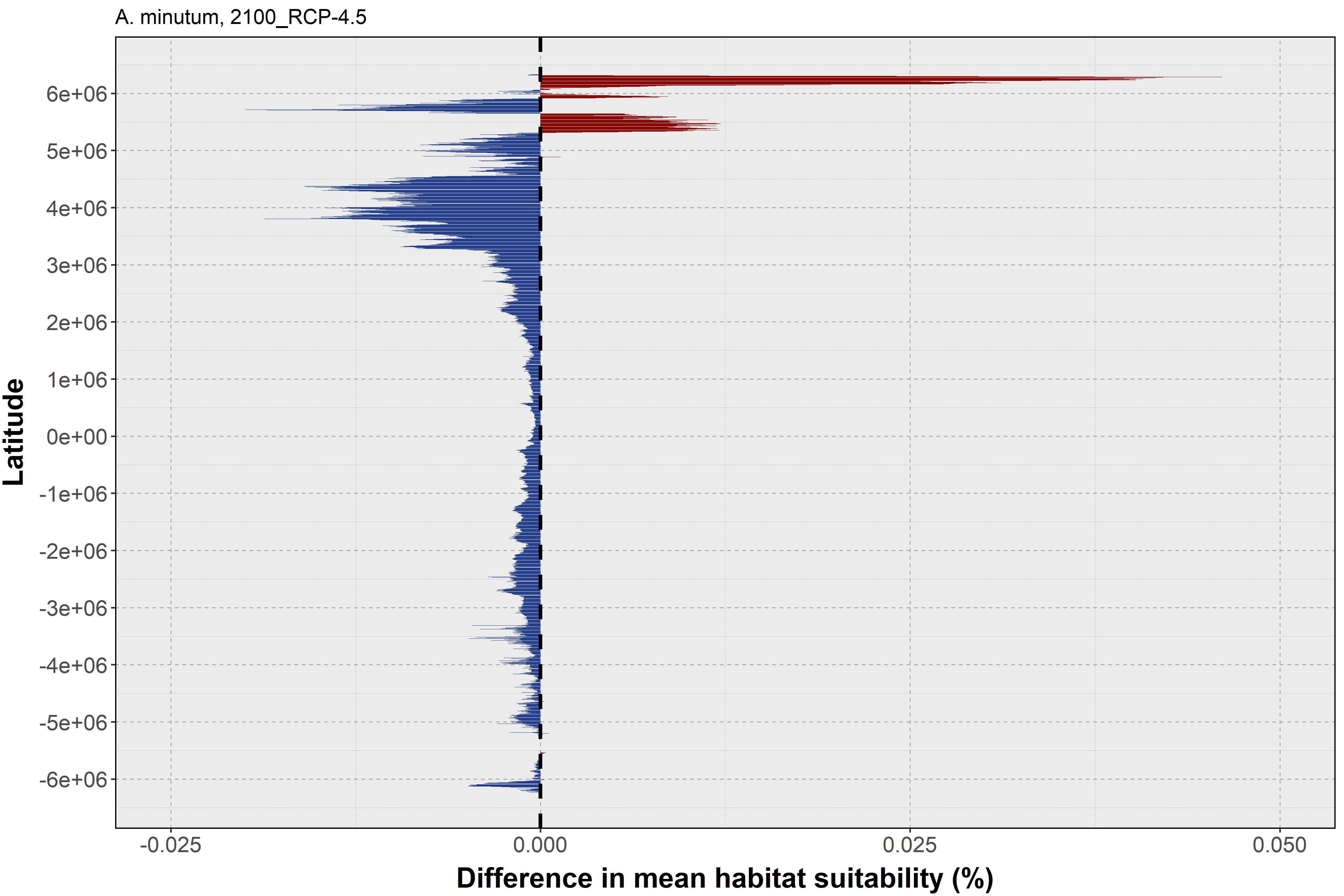

Supplement: Supplementary file 1 [file biology-11-01424-s001.zip › High_Res_Figures/minutum_habdif210045.tiff]

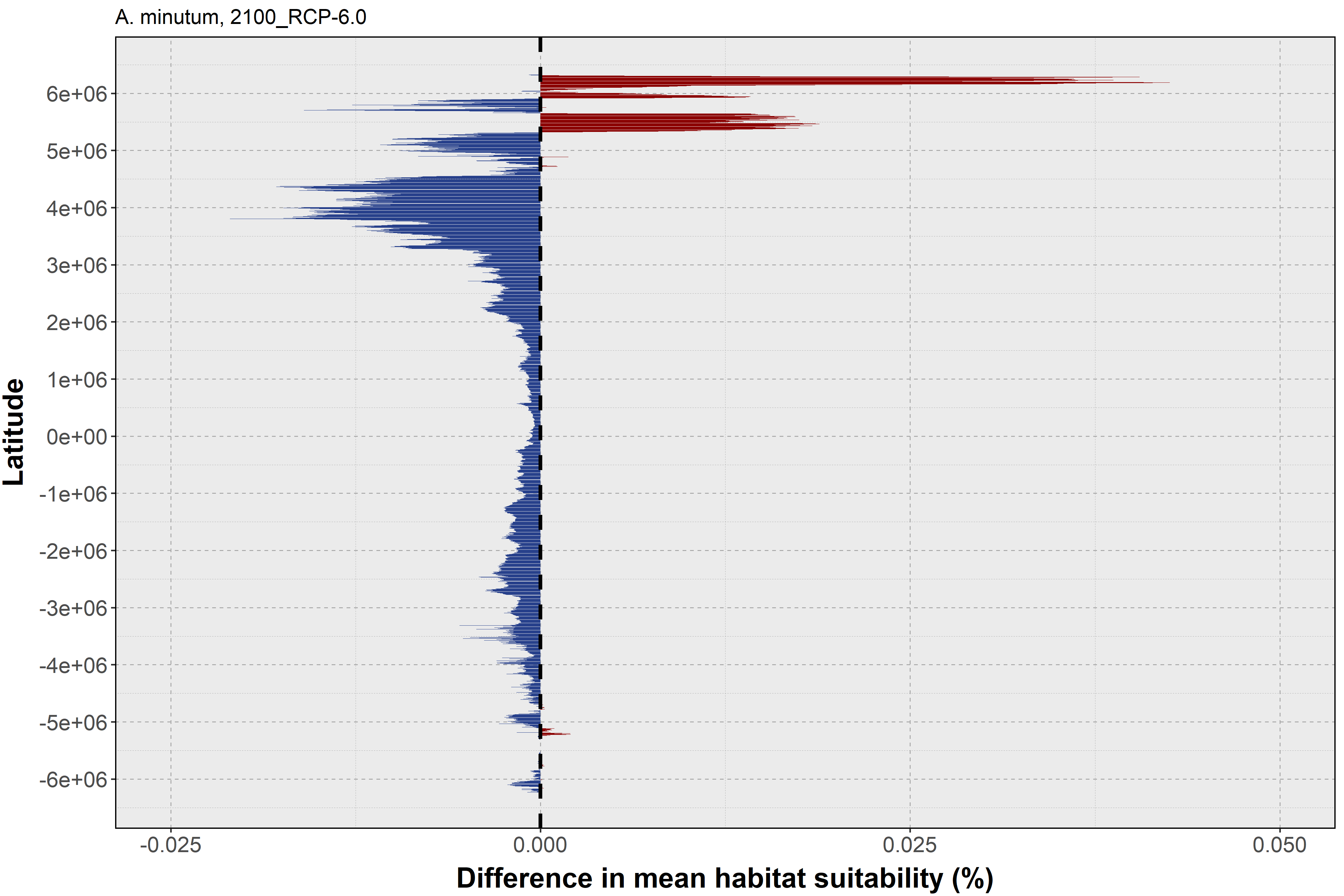

Supplement: Supplementary file 1 [file biology-11-01424-s001.zip › High_Res_Figures/minutum_habdif210060.tiff]

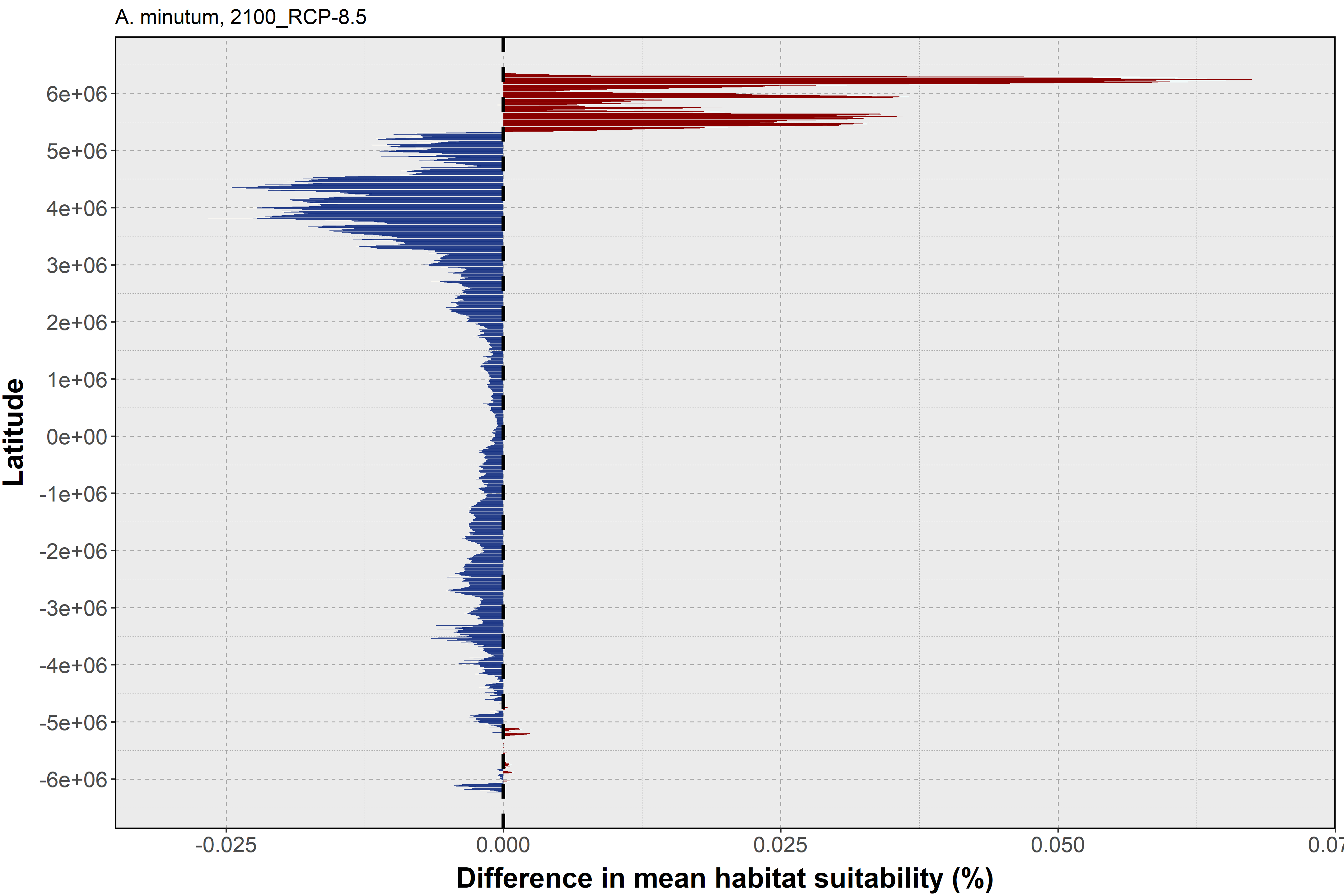

Supplement: Supplementary file 1 [file biology-11-01424-s001.zip › High_Res_Figures/minutum_habdif210085.tiff]

# Alexandrium\_catenella\_2050\_RCP2.6\_binary

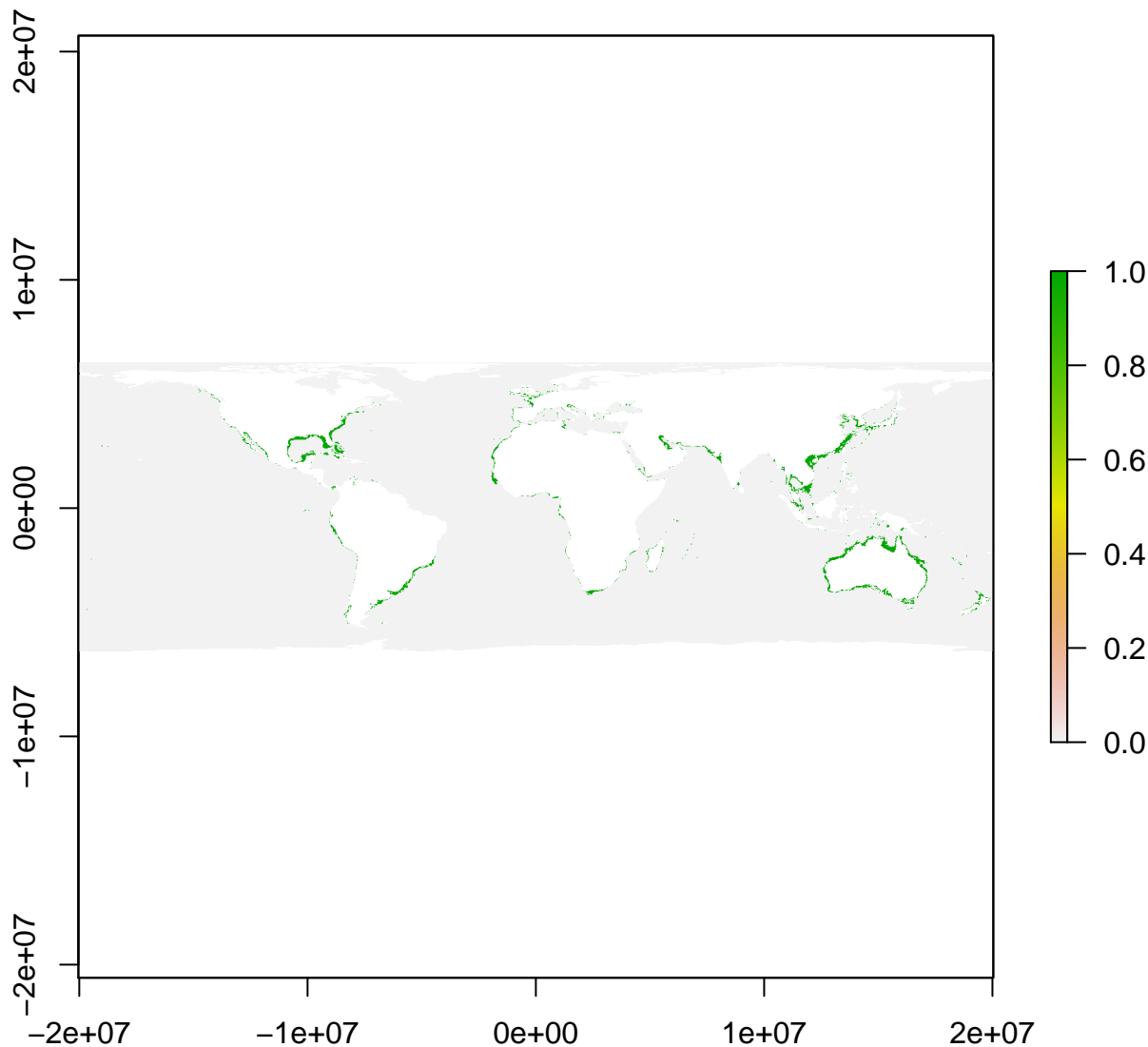

Supplement: Supplementary file 1 [file biology-11-01424-s001.zip › Maps/Binary/a_catenella/2050_RCP2.6_binary.pdf]

# Alexandrium\_catenella\_2050\_RCP4.5\_binary

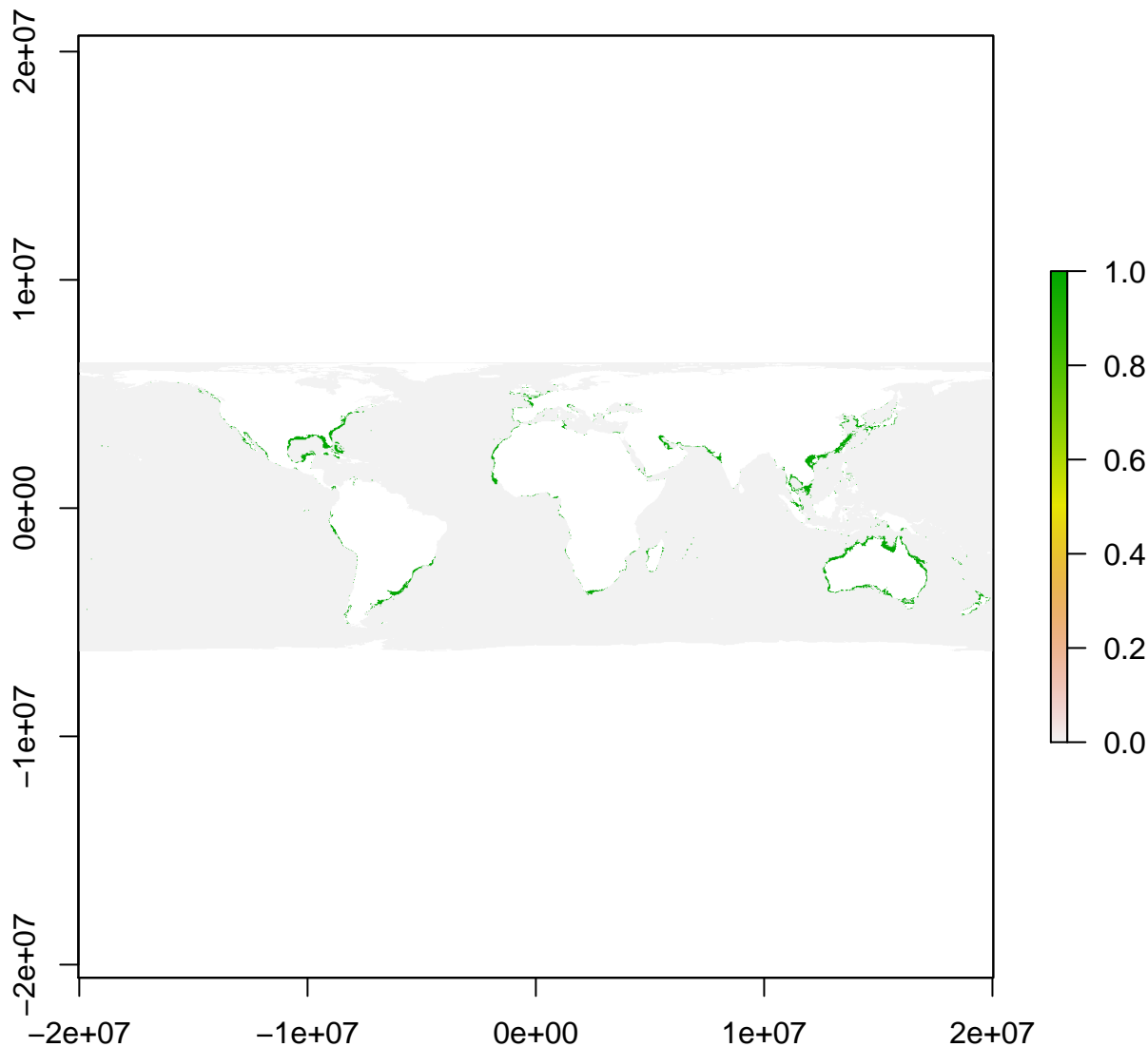

Supplement: Supplementary file 1 [file biology-11-01424-s001.zip › Maps/Binary/a_catenella/2050_RCP4.5_binary.pdf]

# Alexandrium\_catenella\_2050\_RCP6.0\_binary

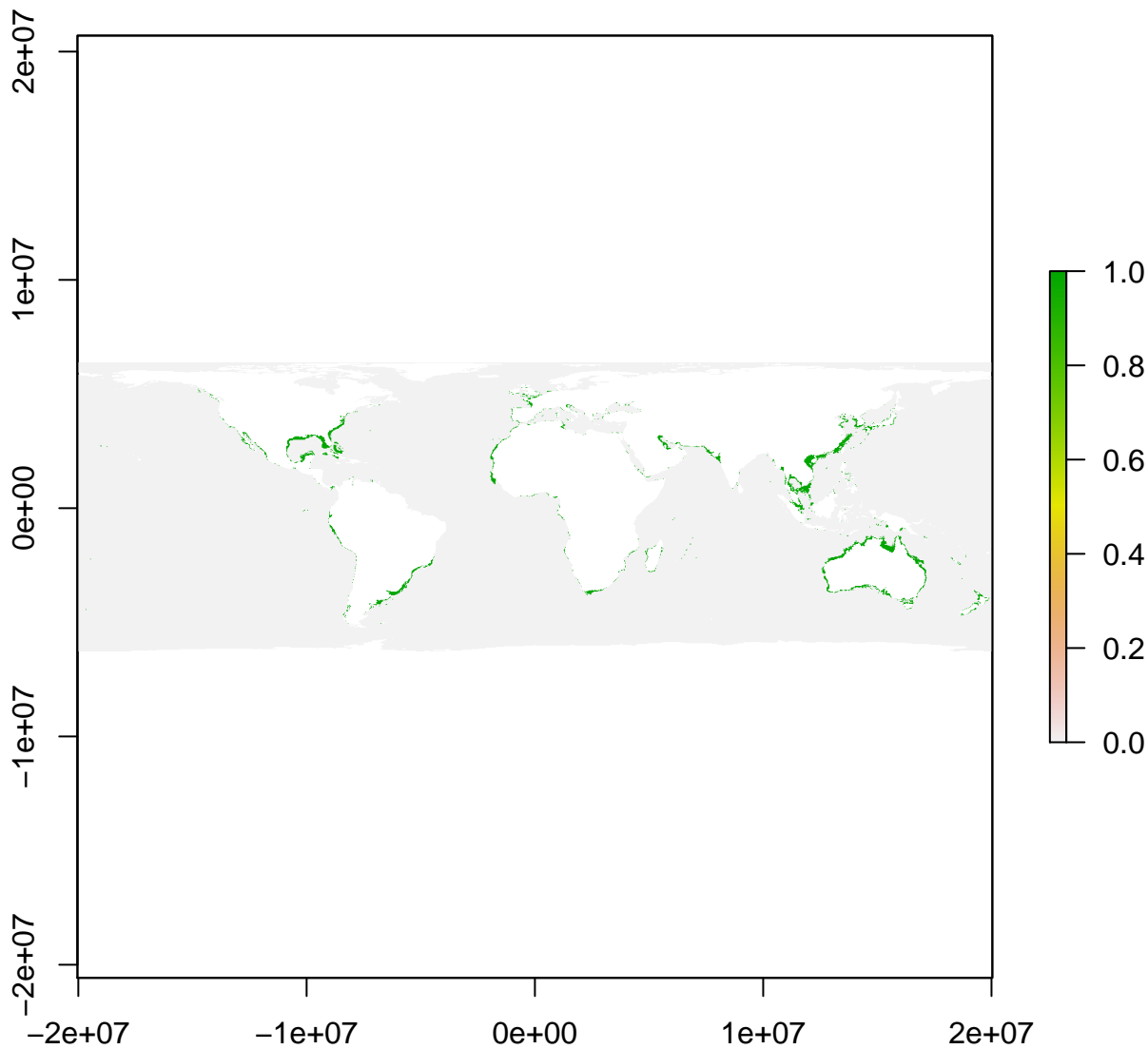

Supplement: Supplementary file 1 [file biology-11-01424-s001.zip › Maps/Binary/a_catenella/2050_RCP6.0_binary.pdf]

# Alexandrium\_catenella\_2050\_RCP8.5\_binary

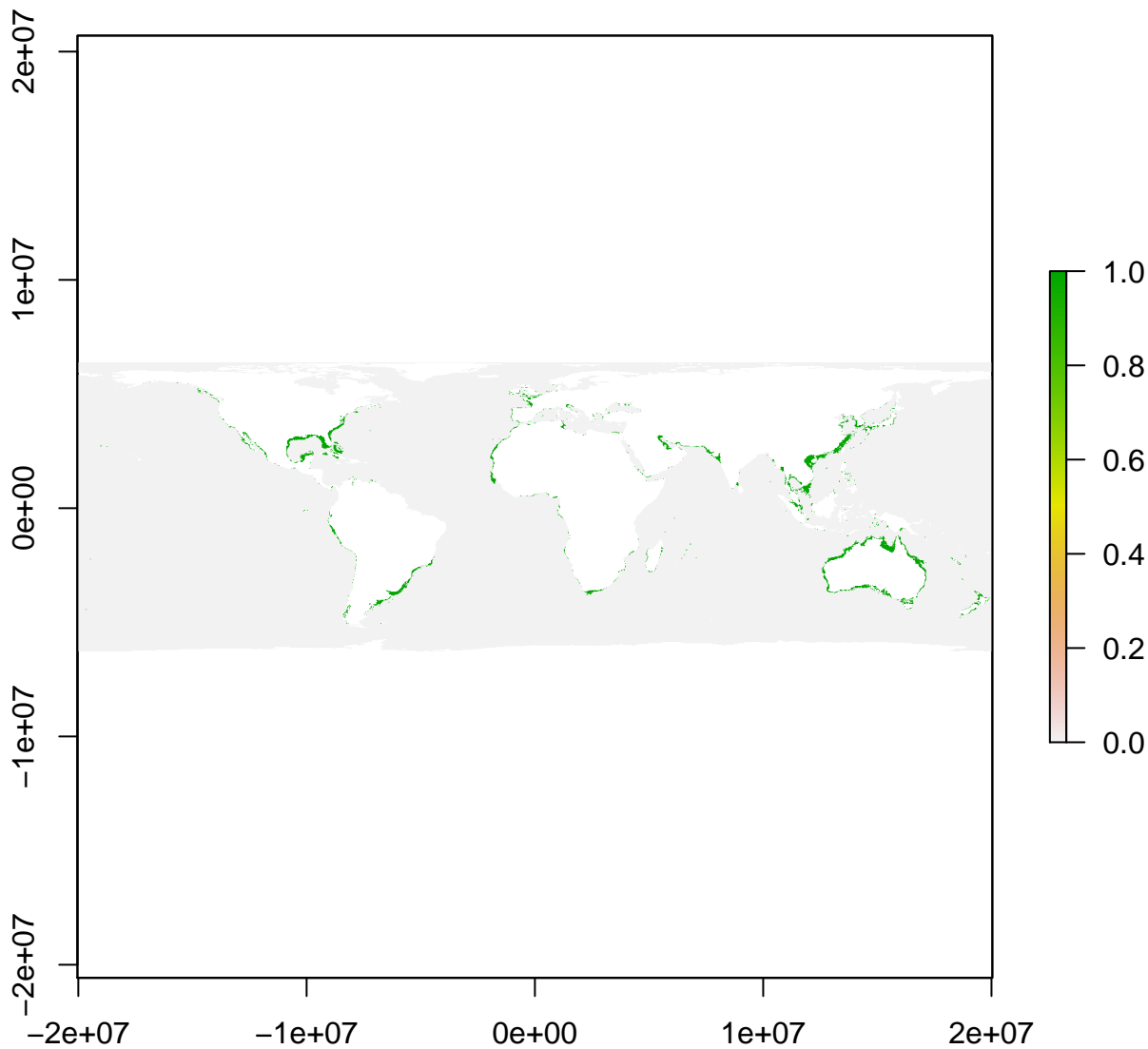

Supplement: Supplementary file 1 [file biology-11-01424-s001.zip › Maps/Binary/a_catenella/2050_RCP8.5_binary.pdf]

# Alexandrium\_catenella\_2100\_RCP2.6\_binary

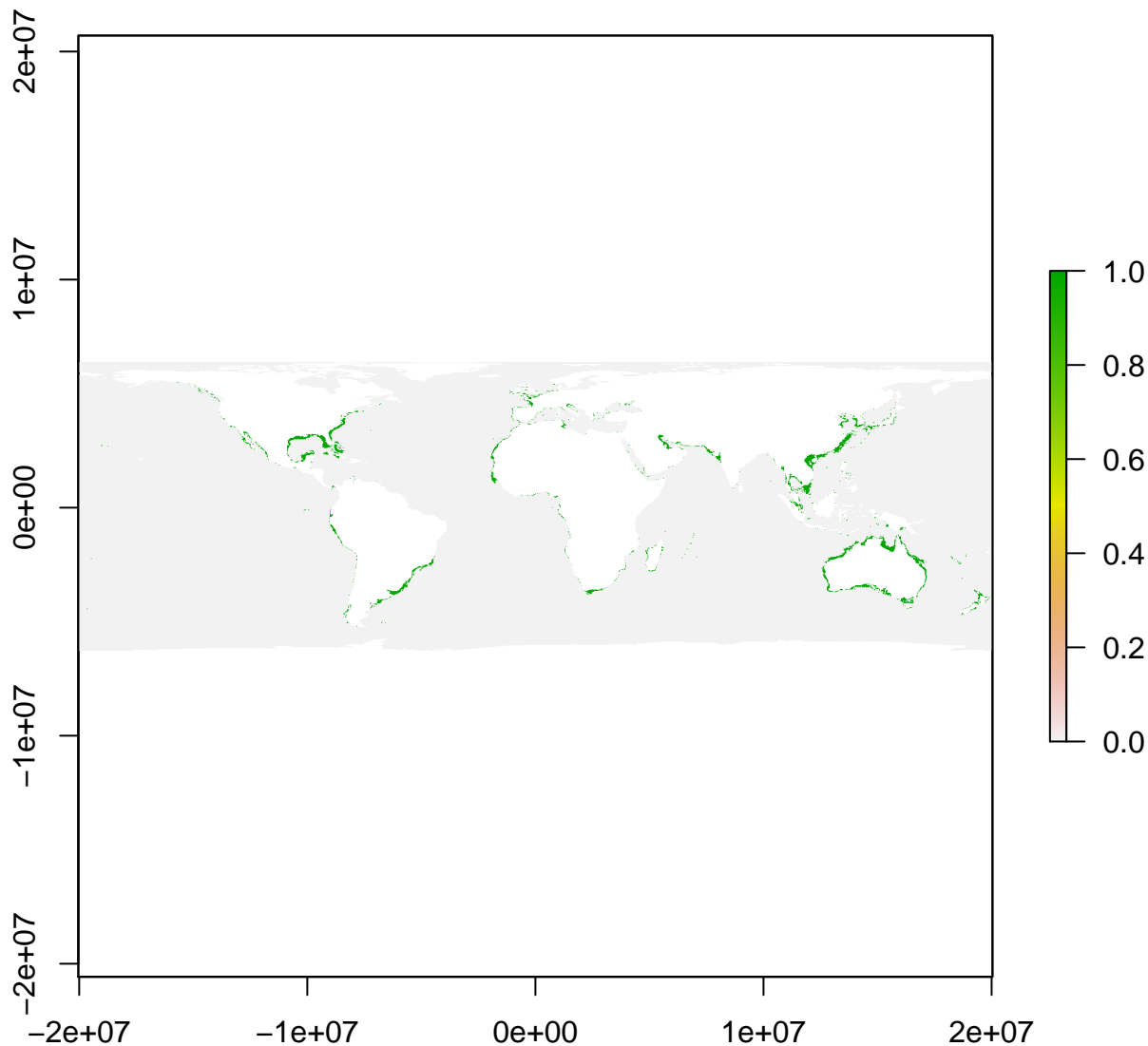

Supplement: Supplementary file 1 [file biology-11-01424-s001.zip › Maps/Binary/a_catenella/2100_RCP2.6_binary.pdf]

# Alexandrium\_catenella\_2100\_RCP4.5\_binary

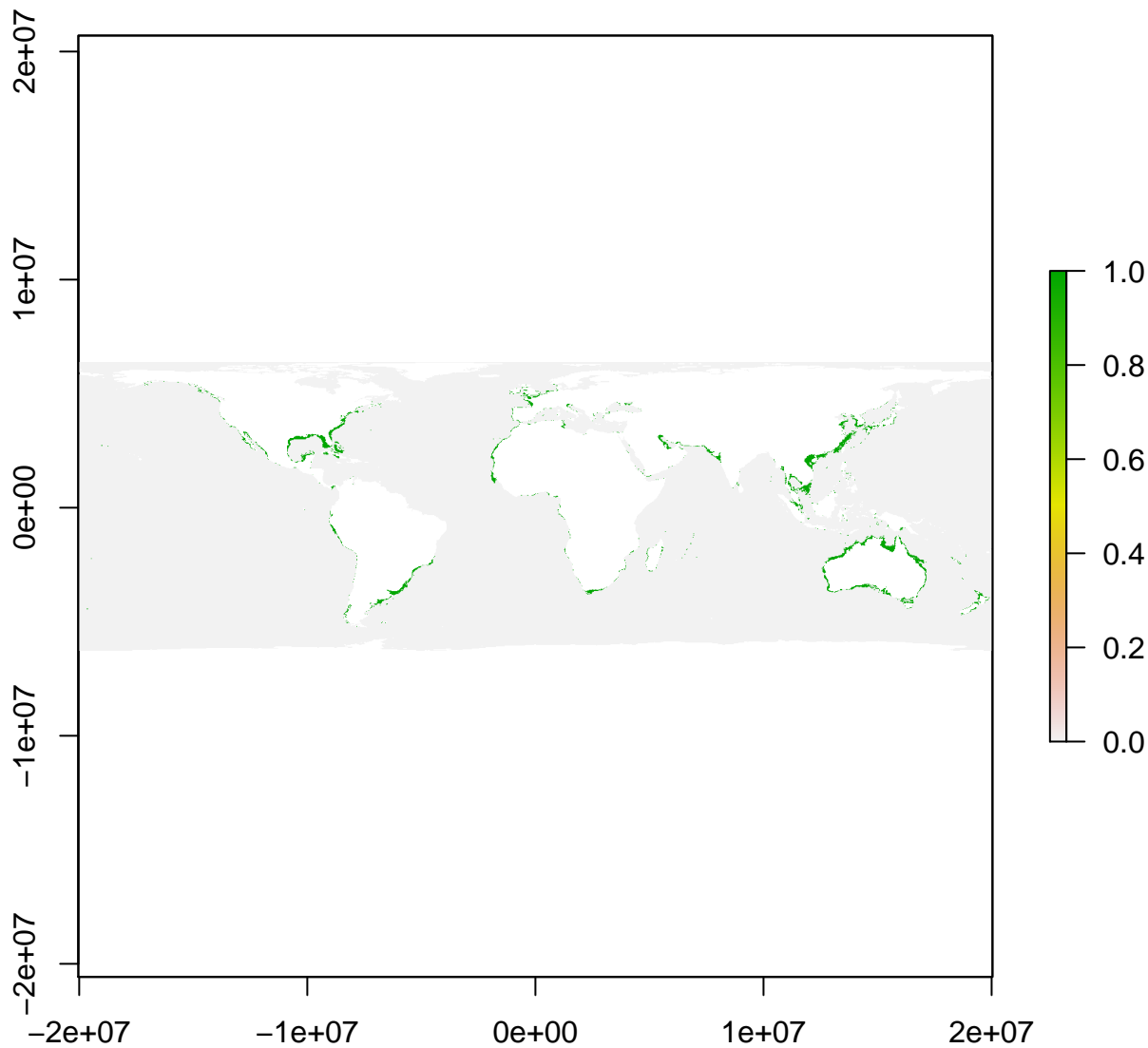

Supplement: Supplementary file 1 [file biology-11-01424-s001.zip › Maps/Binary/a_catenella/2100_RCP4.5_binary.pdf]

# Alexandrium\_catenella\_2100\_RCP6.0\_binary

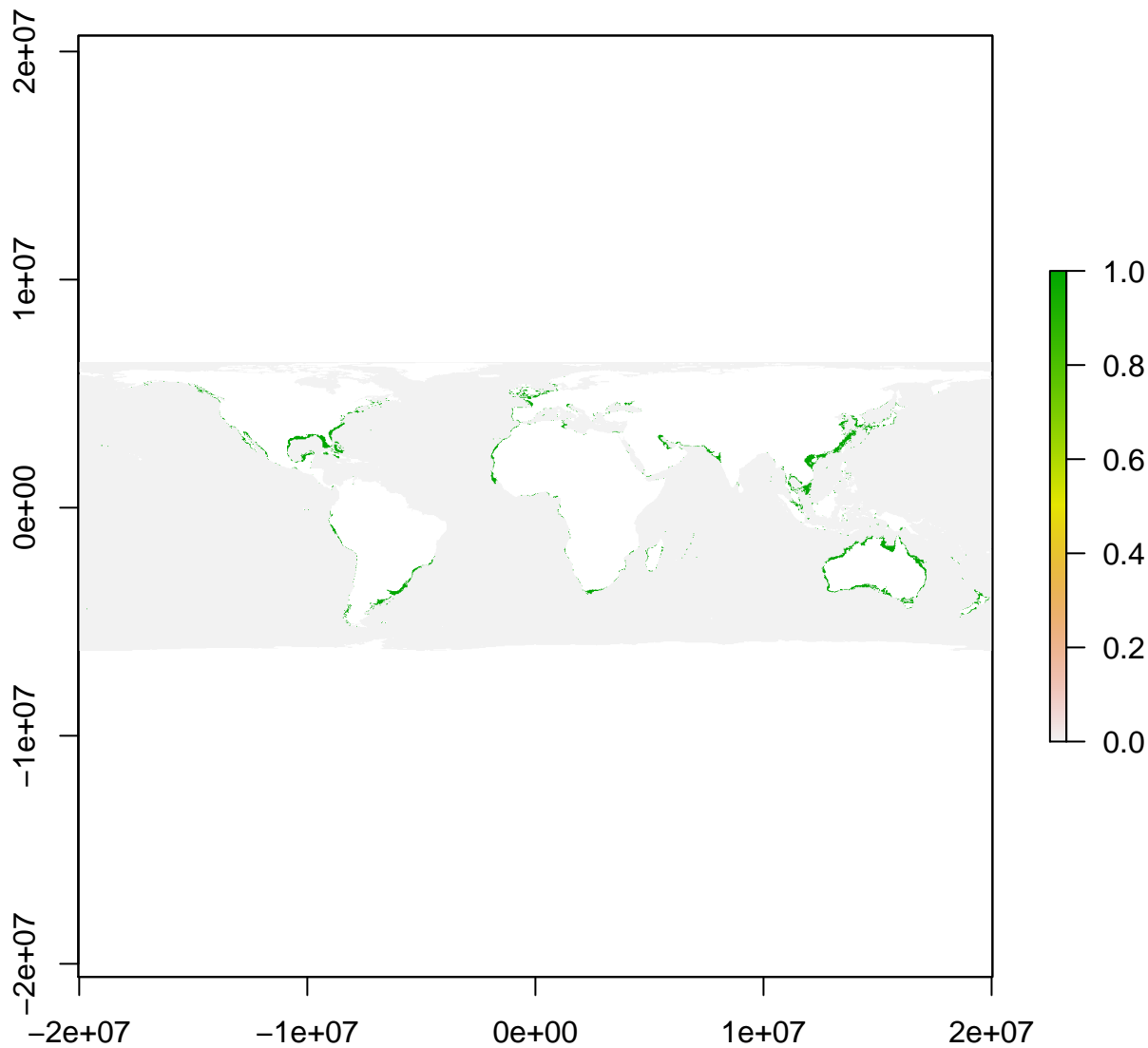

Supplement: Supplementary file 1 [file biology-11-01424-s001.zip › Maps/Binary/a_catenella/2100_RCP6.0_binary.pdf]

# Alexandrium\_catenella\_2100\_RCP8.5\_binary

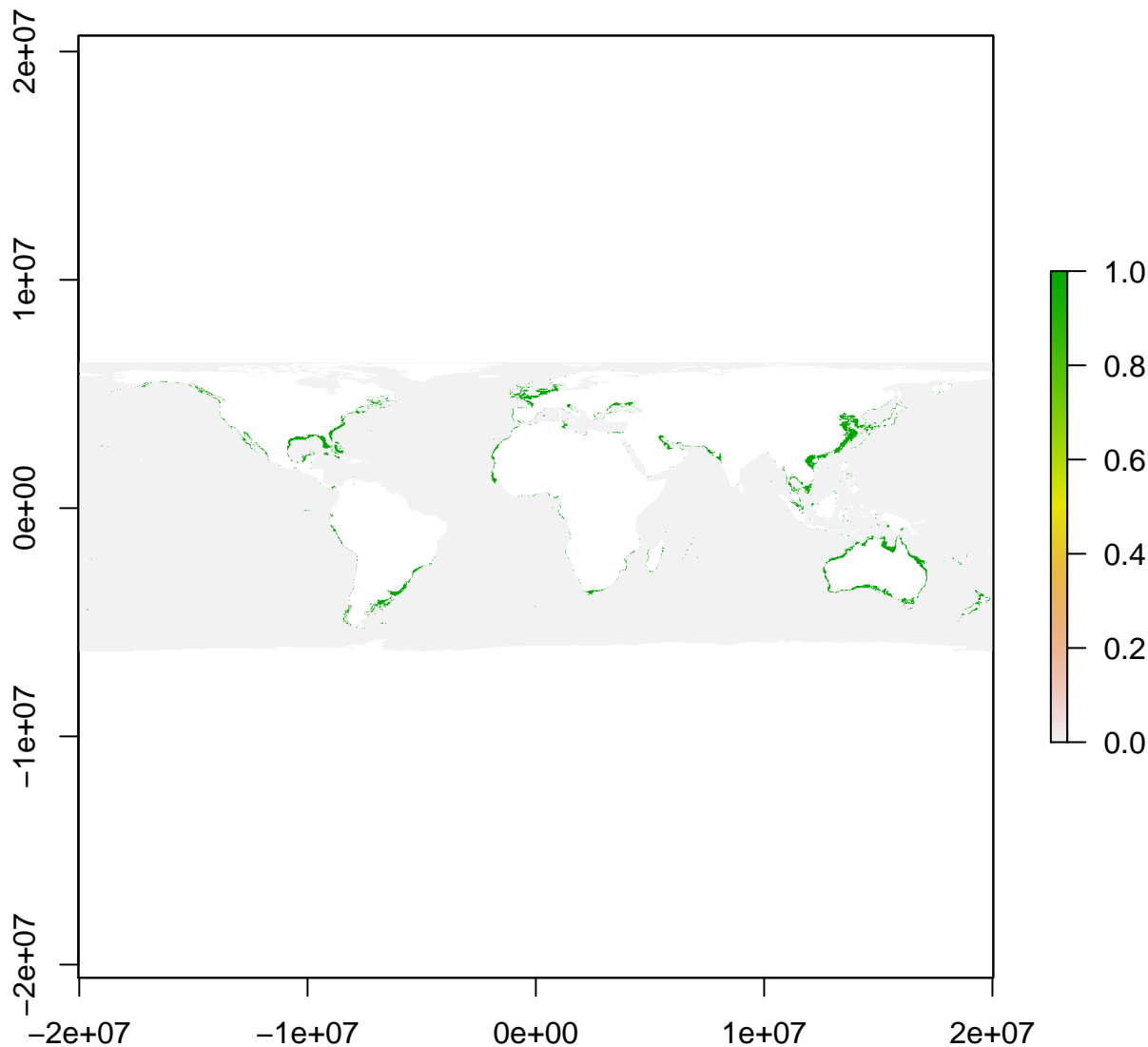

Supplement: Supplementary file 1 [file biology-11-01424-s001.zip › Maps/Binary/a_catenella/2100_RCP8.5_binary.pdf]

# Alexandrium\_catenella\_present\_binary

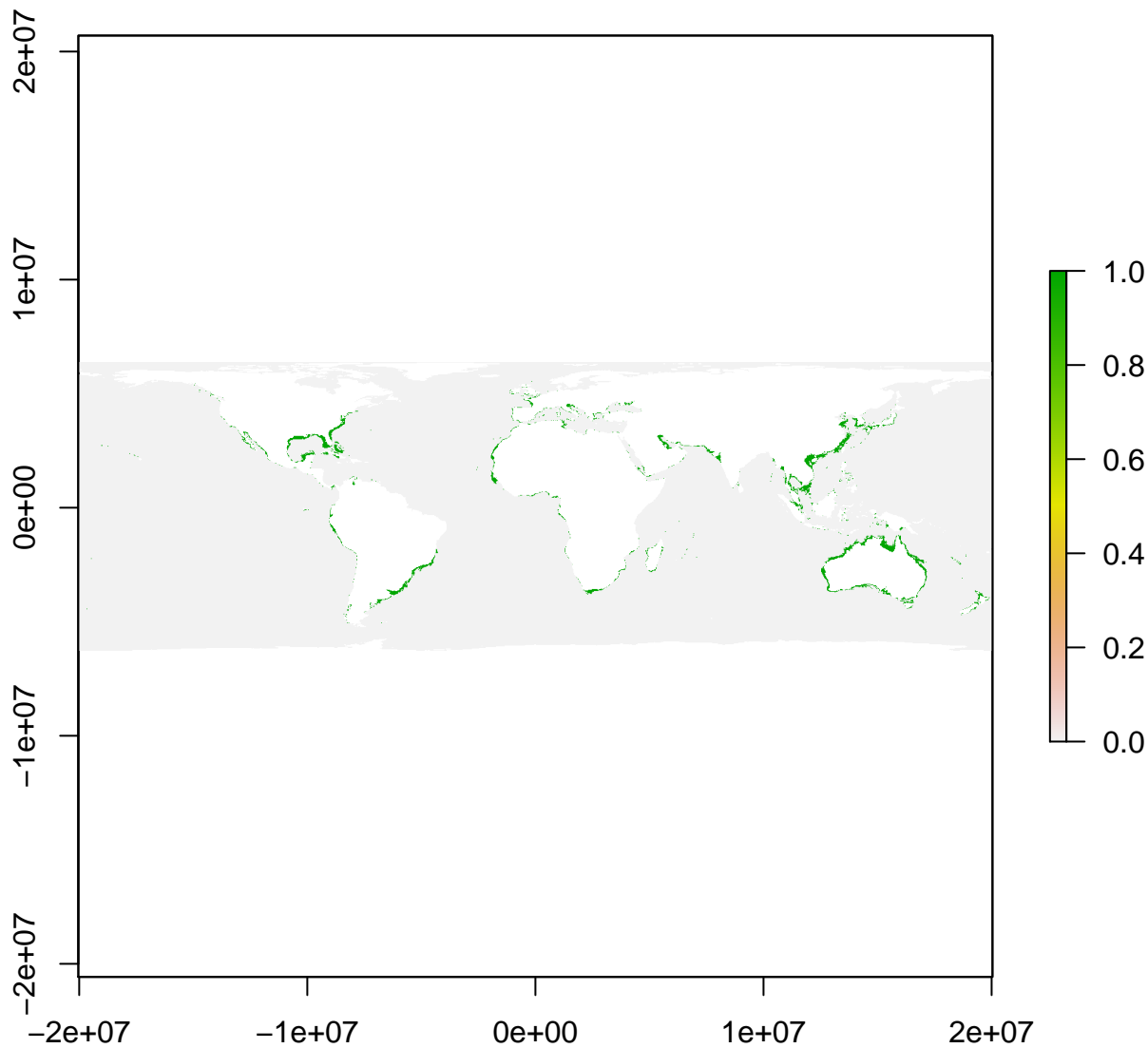

Supplement: Supplementary file 1 [file biology-11-01424-s001.zip › Maps/Binary/a_catenella/present_binary.pdf]

# Alexandrium\_minutum\_2050\_RCP2.6\_binary

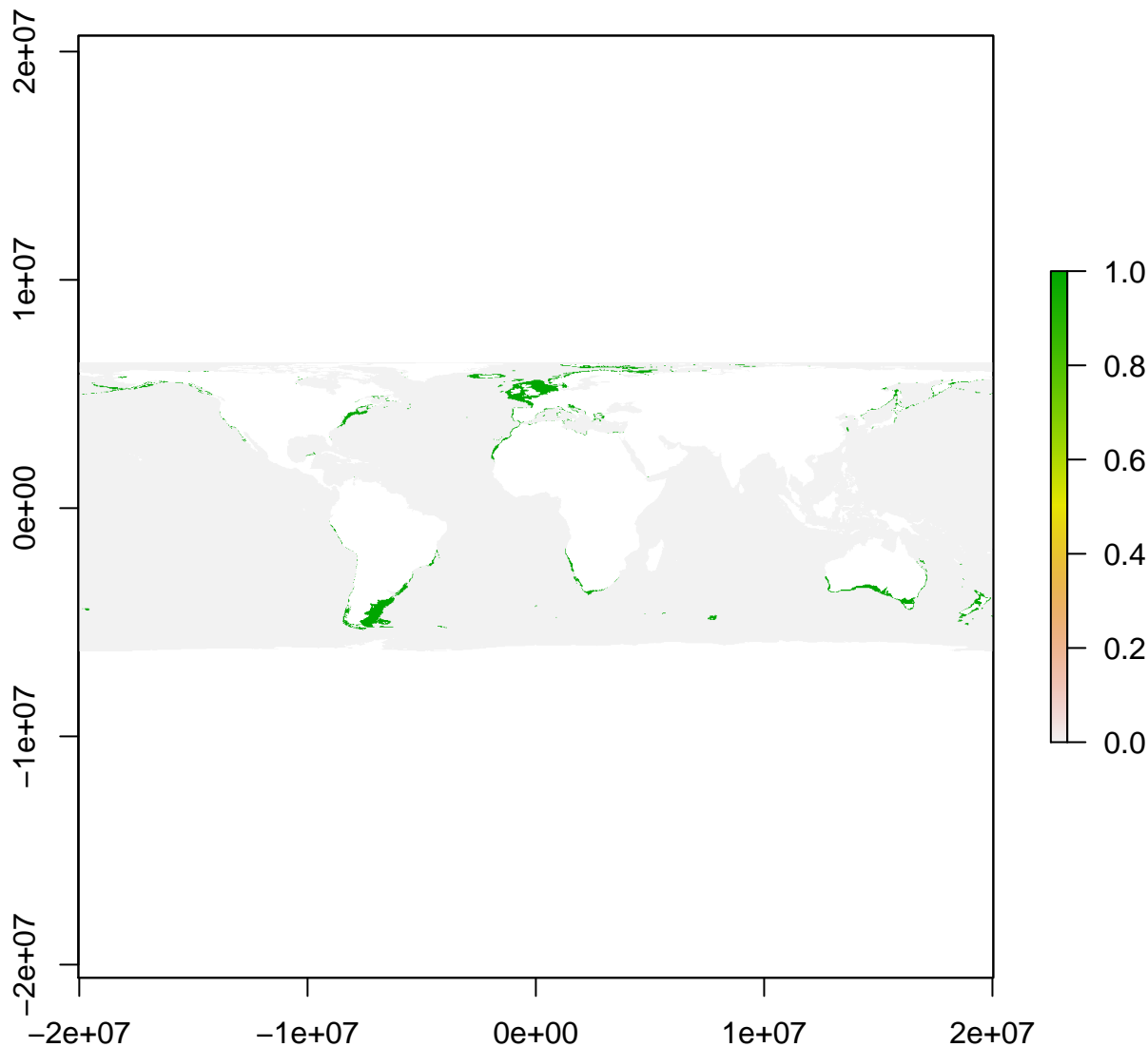

Supplement: Supplementary file 1 [file biology-11-01424-s001.zip › Maps/Binary/a_minutum/2050_RCP2.6_binary.pdf]

# Alexandrium\_minutum\_2050\_RCP4.5\_binary

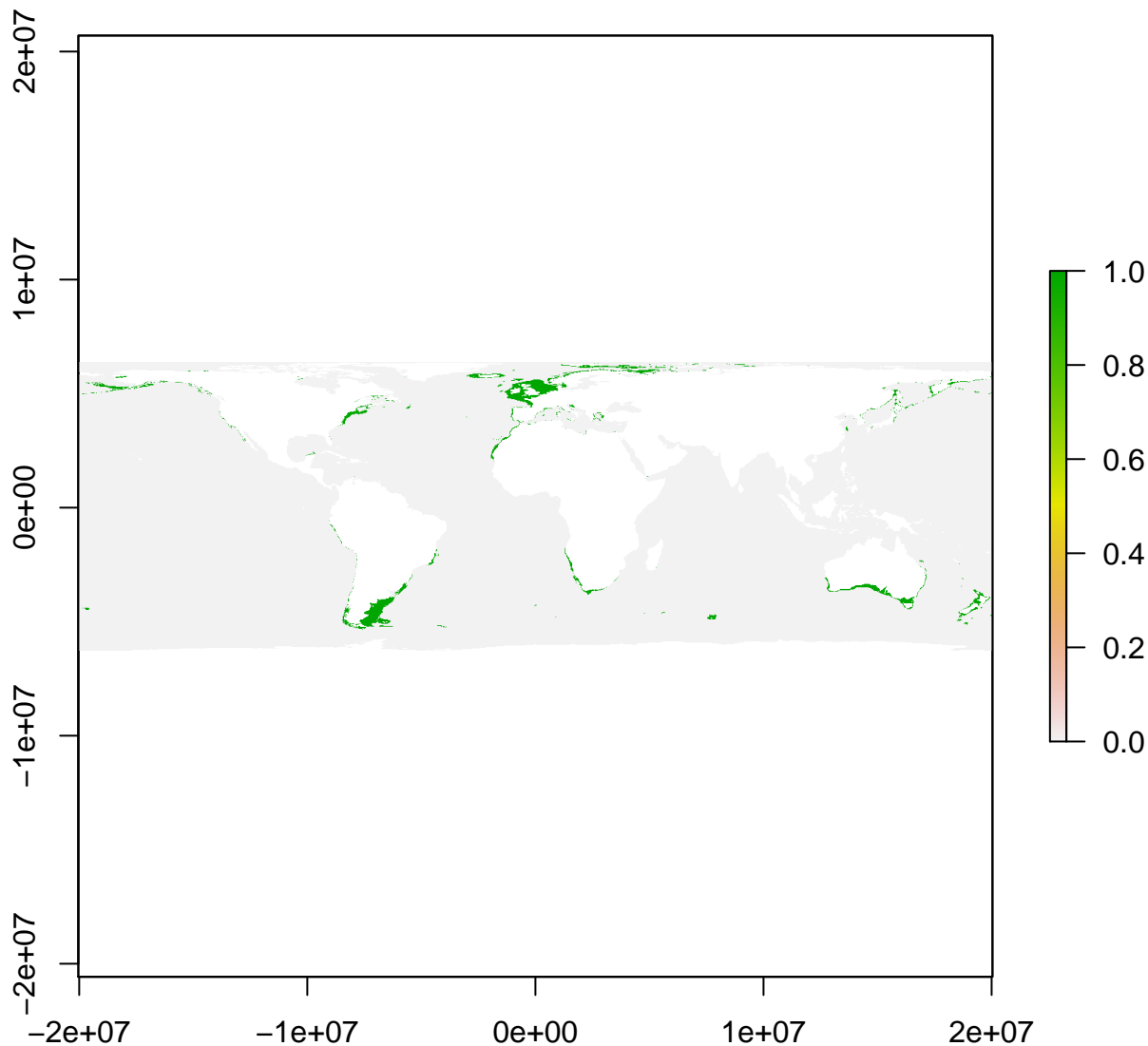

Supplement: Supplementary file 1 [file biology-11-01424-s001.zip › Maps/Binary/a_minutum/2050_RCP4.5_binary.pdf]

# Alexandrium\_minutum\_2050\_RCP6.0\_binary

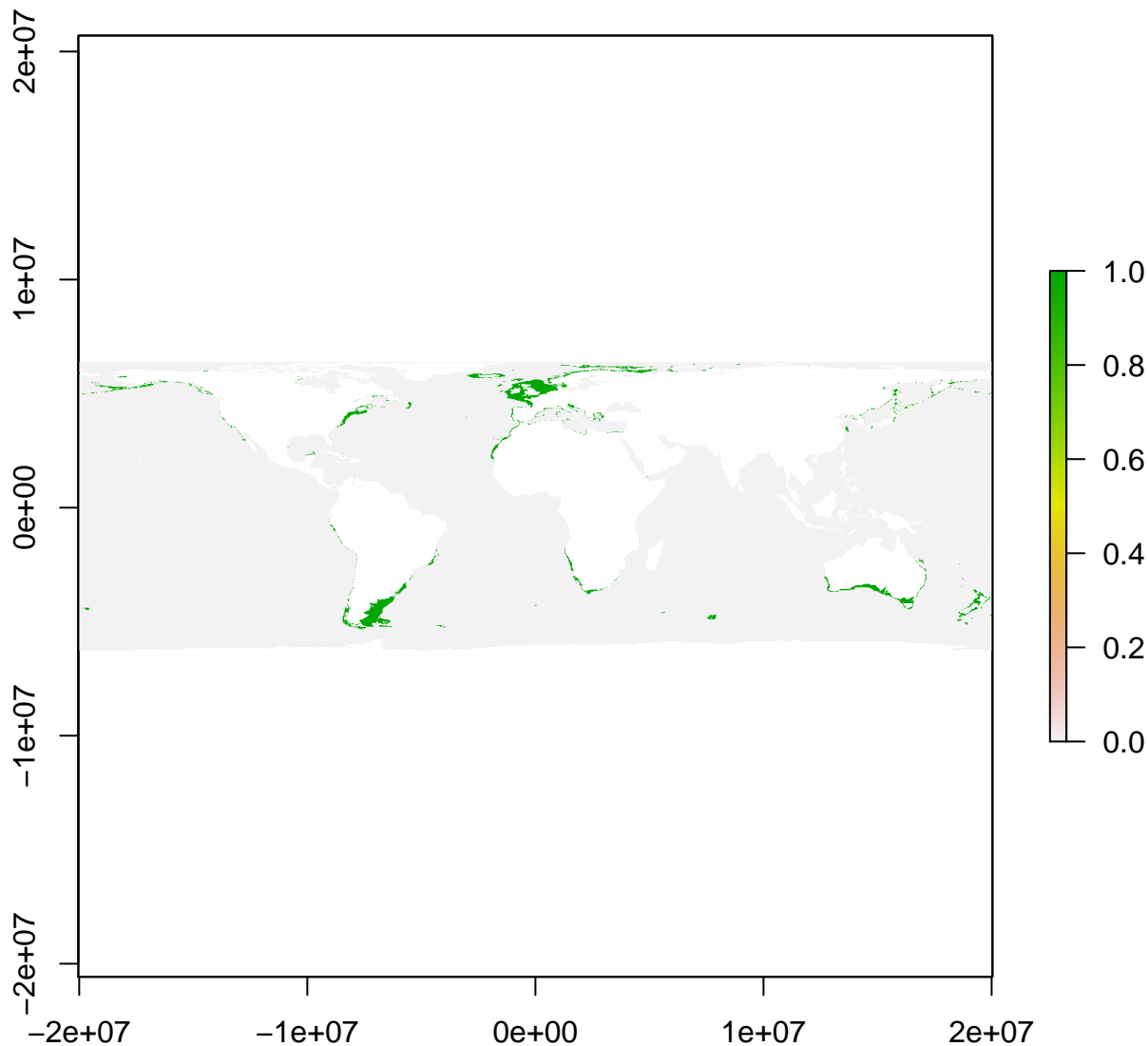

Supplement: Supplementary file 1 [file biology-11-01424-s001.zip › Maps/Binary/a_minutum/2050_RCP6.0_binary.pdf]

# Alexandrium\_minutum\_2050\_RCP8.5\_binary

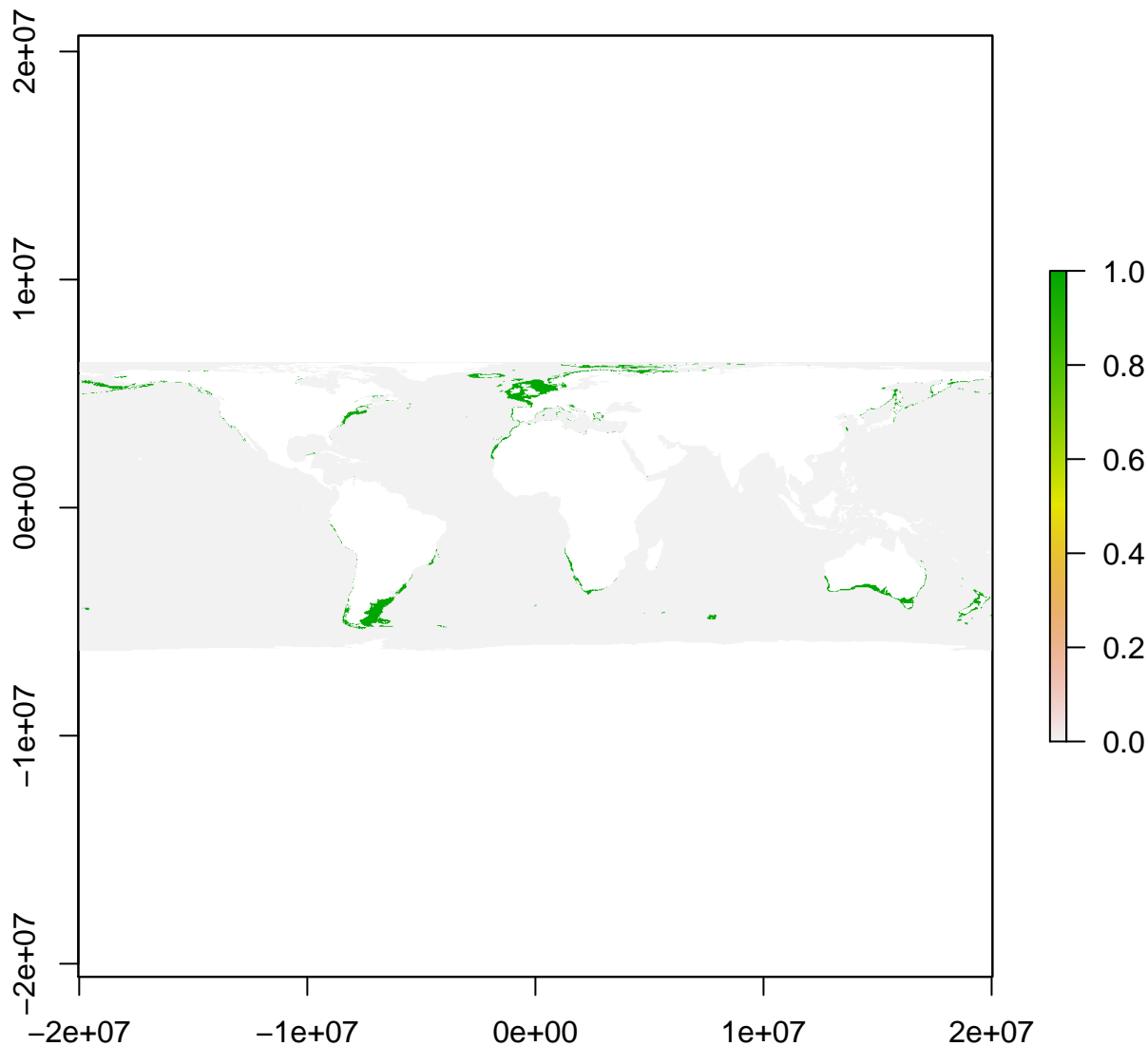

Supplement: Supplementary file 1 [file biology-11-01424-s001.zip › Maps/Binary/a_minutum/2050_RCP8.5_binary.pdf]

# Alexandrium\_minutum\_2100\_RCP2.6\_binary

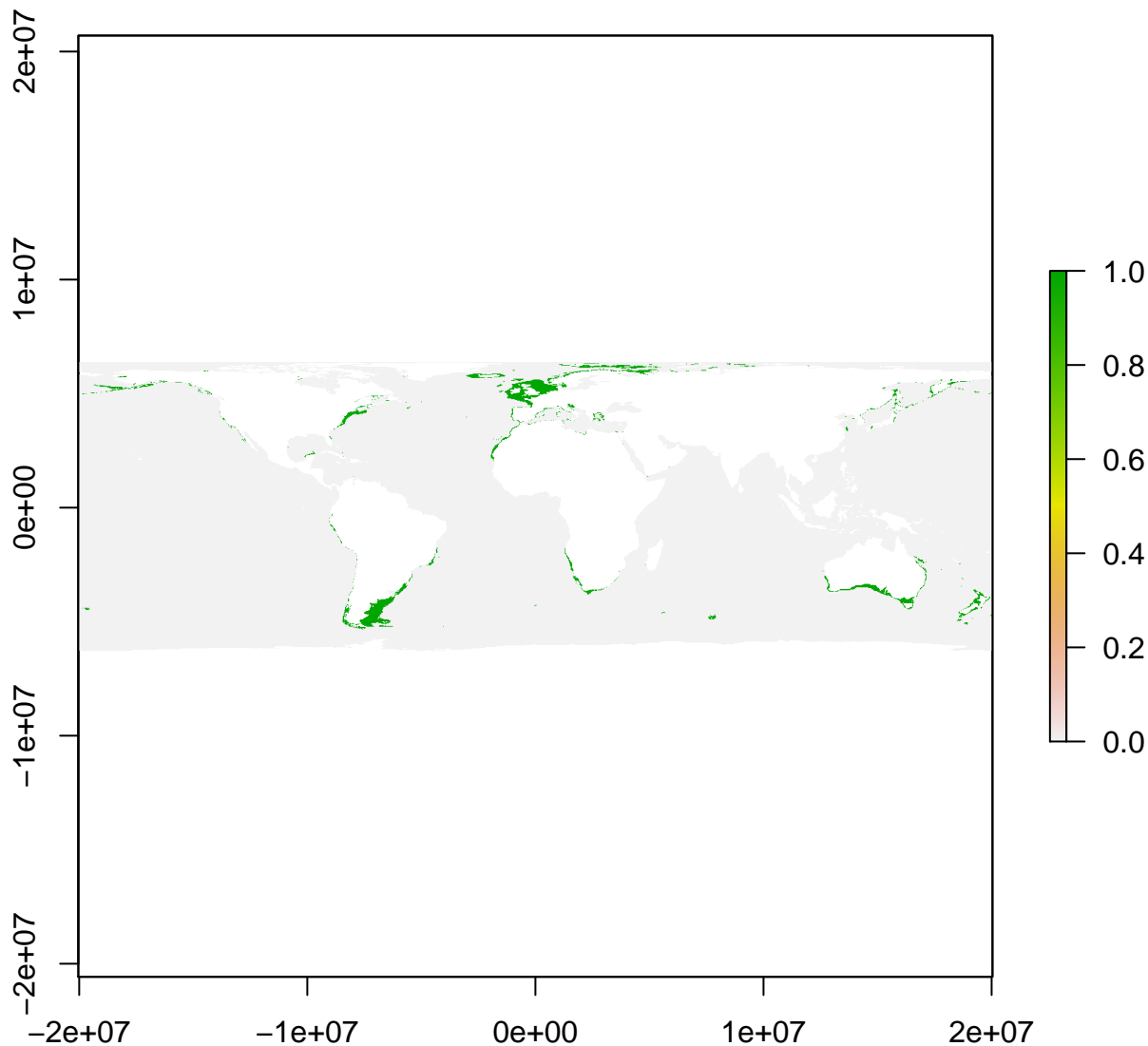

Supplement: Supplementary file 1 [file biology-11-01424-s001.zip › Maps/Binary/a_minutum/2100_RCP2.6_binary.pdf]

# Alexandrium\_minutum\_2100\_RCP4.5\_binary

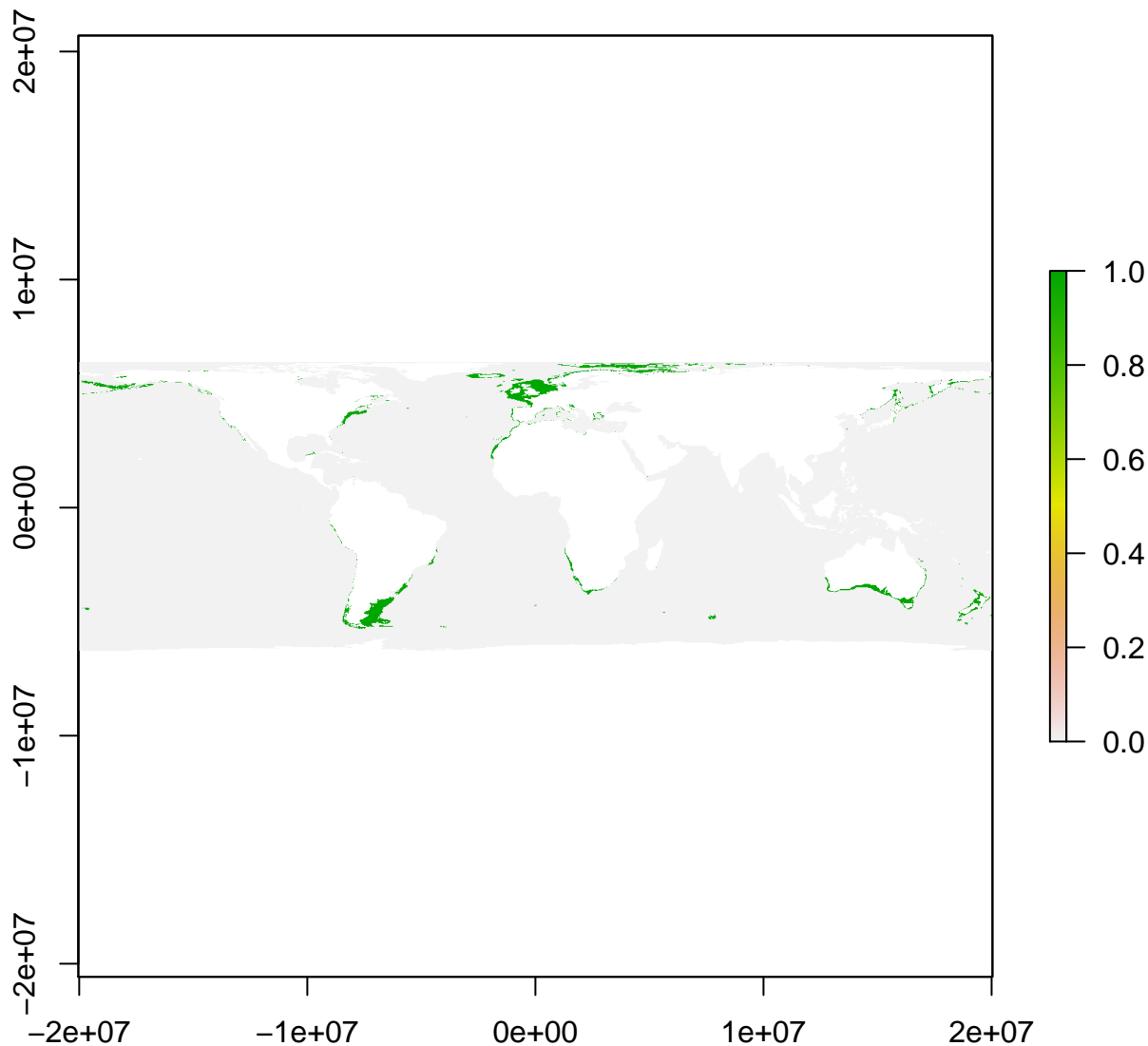

Supplement: Supplementary file 1 [file biology-11-01424-s001.zip › Maps/Binary/a_minutum/2100_RCP4.5_binary.pdf]

# Alexandrium\_minutum\_2100\_RCP6.0\_binary

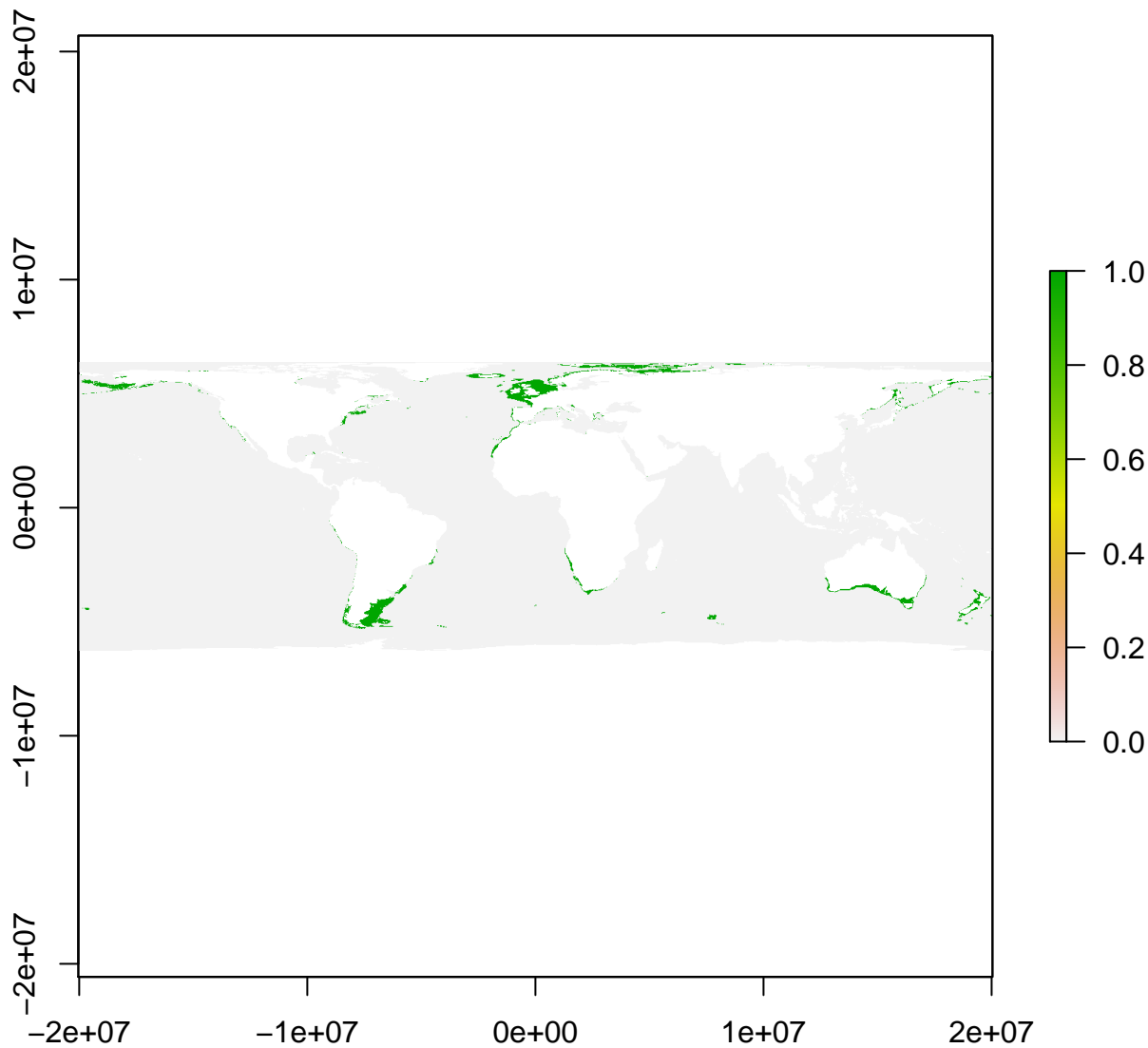

Supplement: Supplementary file 1 [file biology-11-01424-s001.zip › Maps/Binary/a_minutum/2100_RCP6.0_binary.pdf]

# Alexandrium\_minutum\_2100\_RCP8.5\_binary

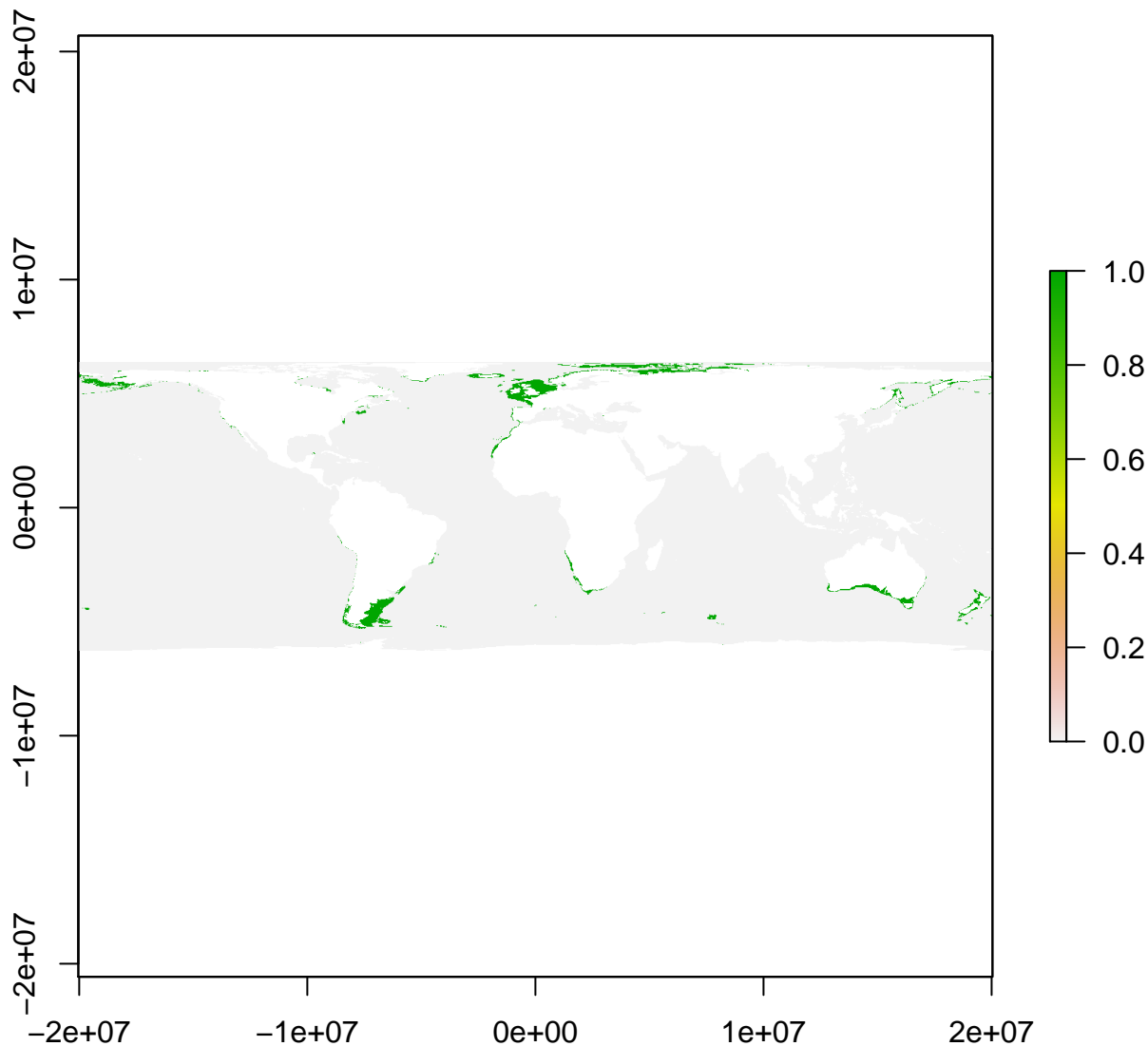

Supplement: Supplementary file 1 [file biology-11-01424-s001.zip › Maps/Binary/a_minutum/2100_RCP8.5_binary.pdf]

# Alexandrium\_minutum\_present\_binary

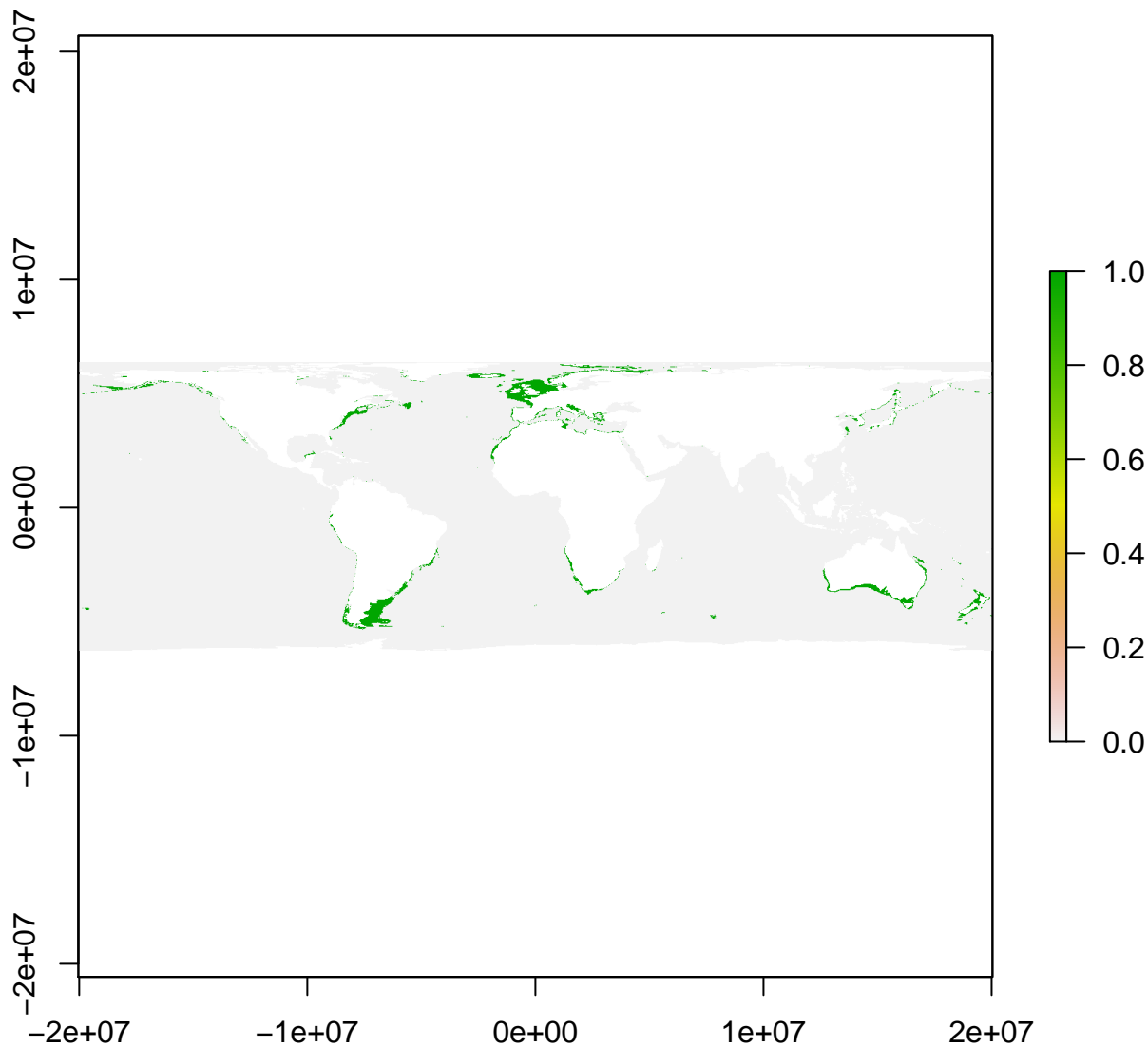

Supplement: Supplementary file 1 [file biology-11-01424-s001.zip › Maps/Binary/a_minutum/present_binary.pdf]

# Gymnodinium\_catenatum\_2050\_RCP2.6\_binary

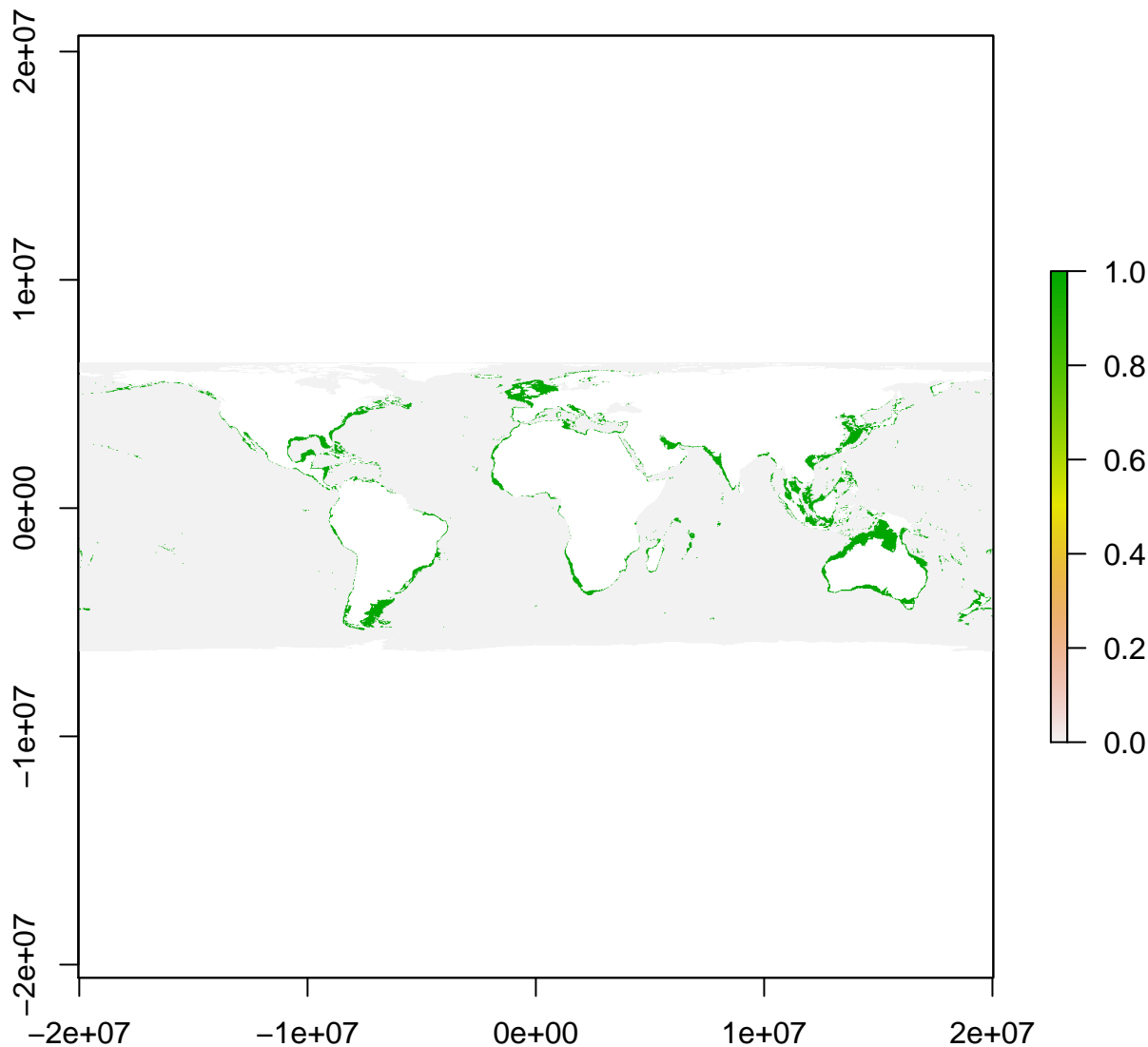

Supplement: Supplementary file 1 [file biology-11-01424-s001.zip › Maps/Binary/g_catenatum/2050_RCP2.6_binary.pdf]

# Gymnodinium\_catenatum\_2050\_RCP4.5\_binary

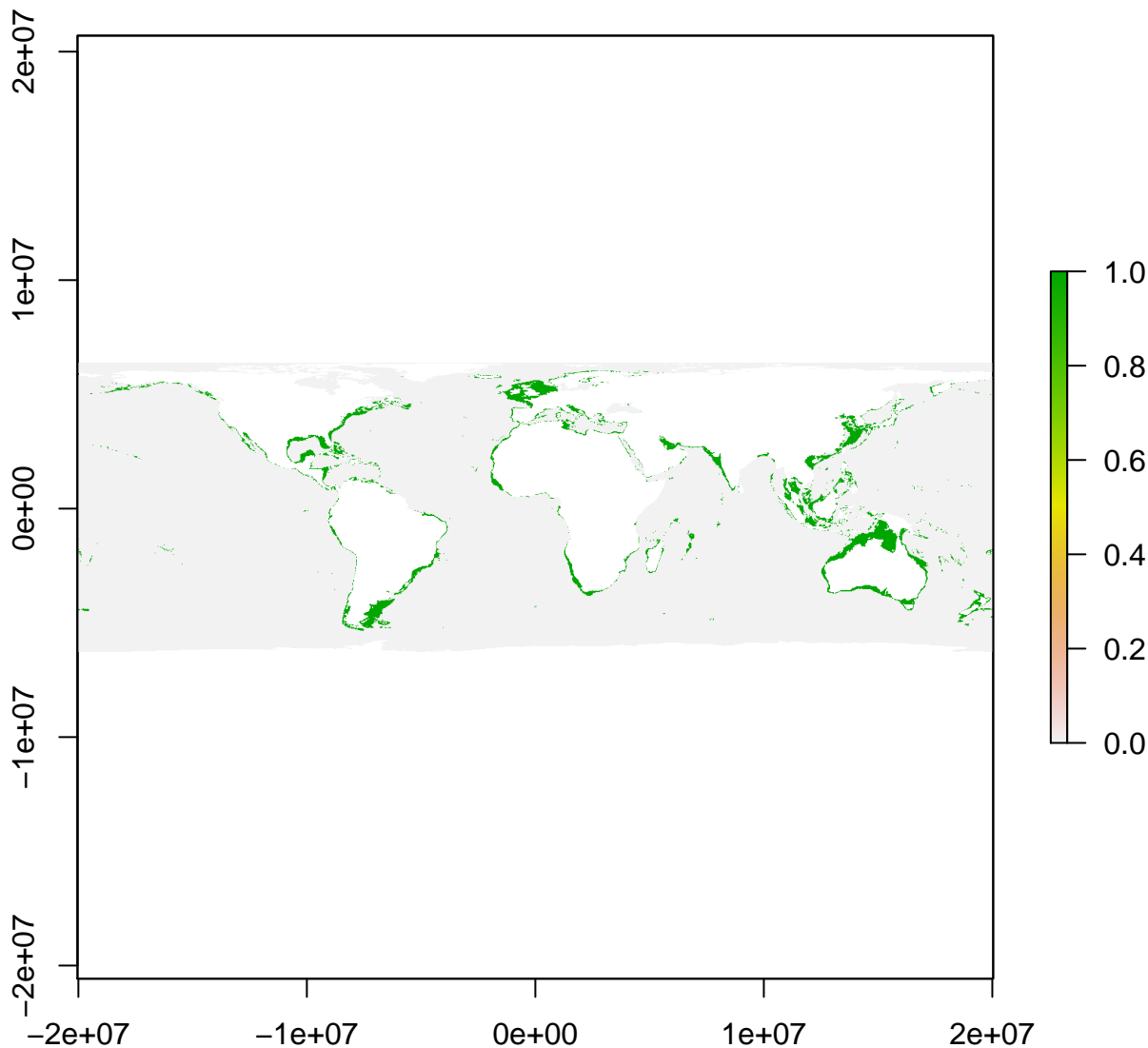

Supplement: Supplementary file 1 [file biology-11-01424-s001.zip › Maps/Binary/g_catenatum/2050_RCP4.5_binary.pdf]

# Gymnodinium\_catenatum\_2050\_RCP6.0\_binary

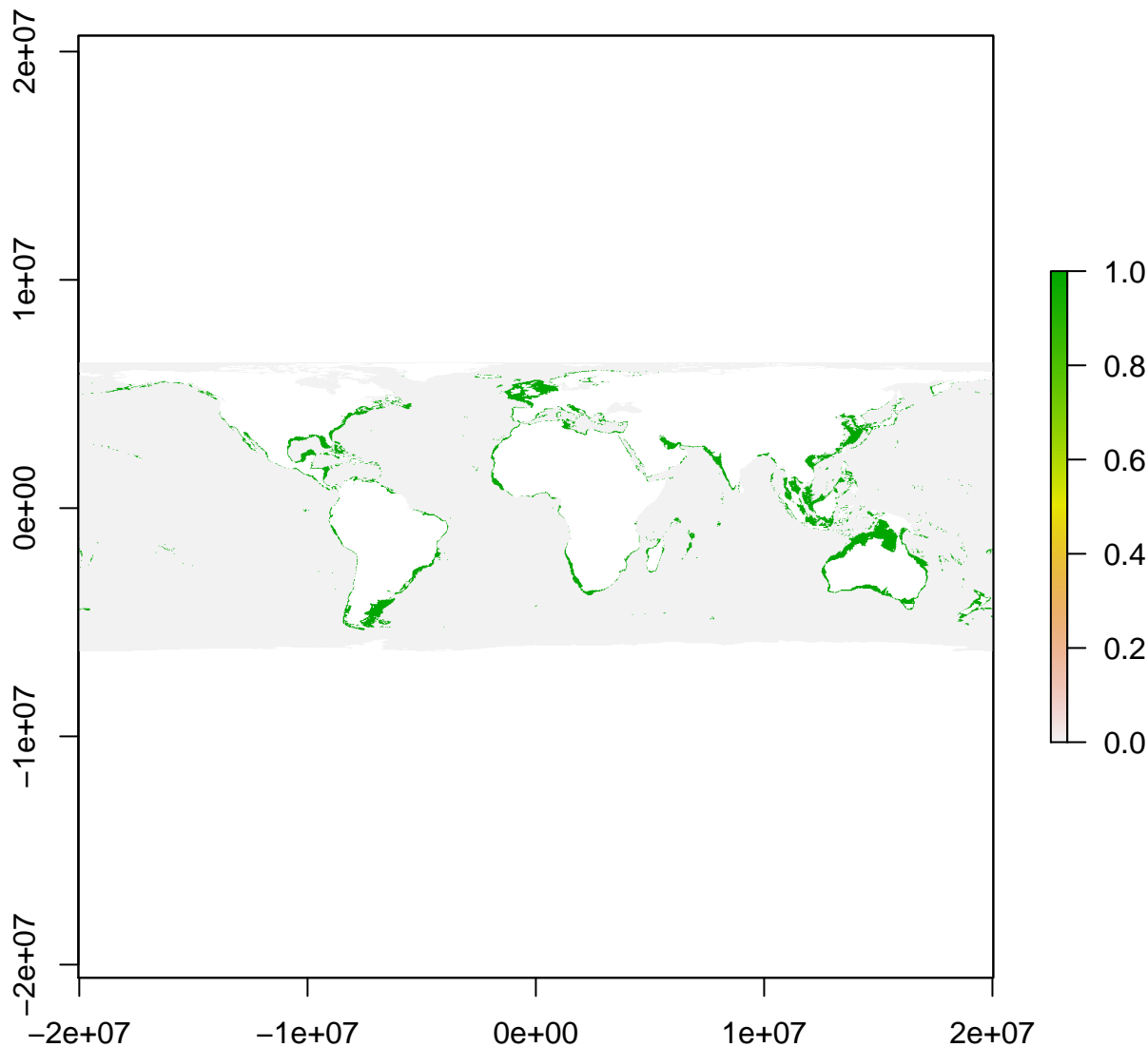

Supplement: Supplementary file 1 [file biology-11-01424-s001.zip › Maps/Binary/g_catenatum/2050_RCP6.0_binary.pdf]

# Gymnodinium\_catenatum\_2050\_RCP8.5\_binary

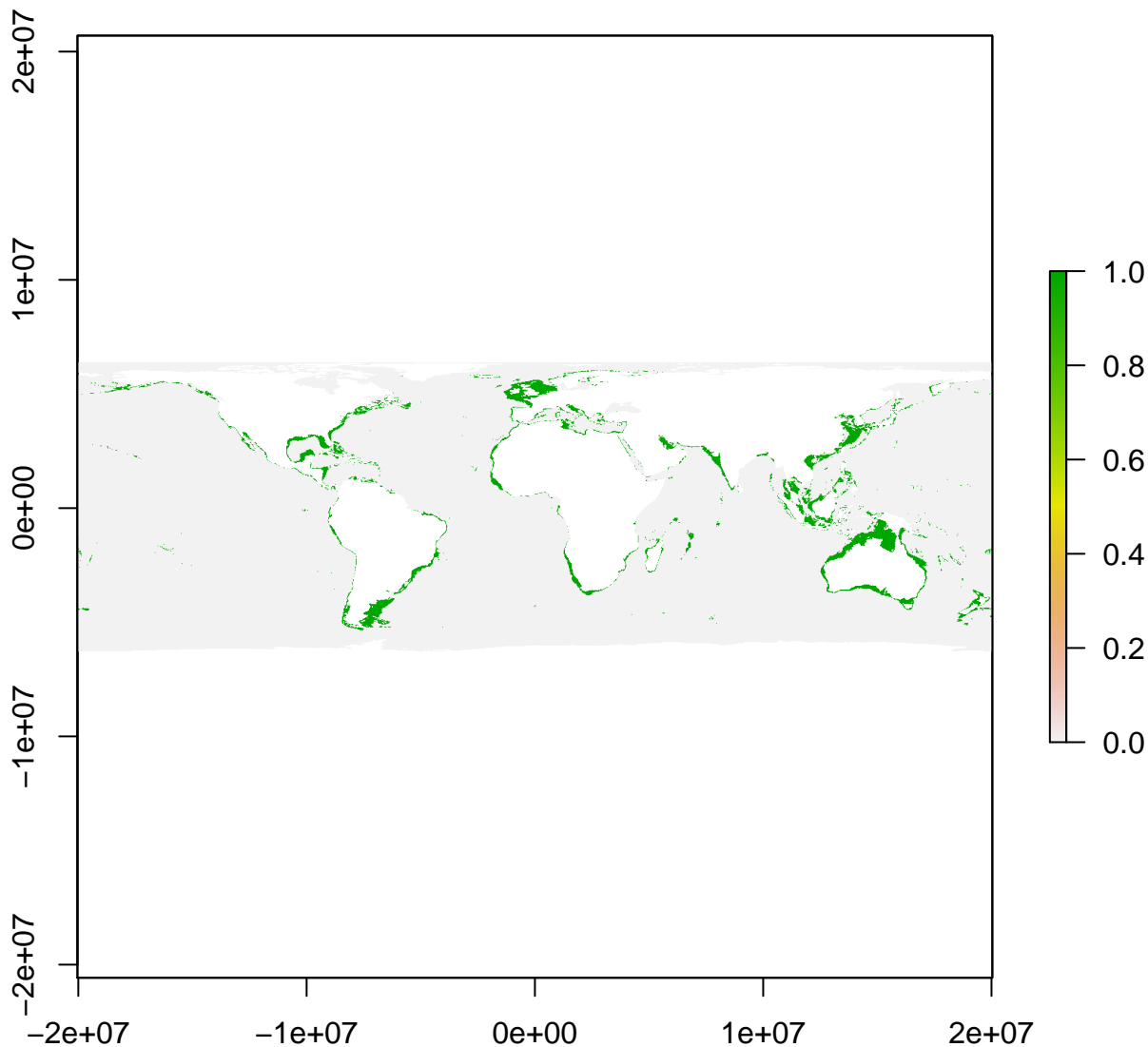

Supplement: Supplementary file 1 [file biology-11-01424-s001.zip › Maps/Binary/g_catenatum/2050_RCP8.5_binary.pdf]

# Gymnodinium\_catenatum\_2100\_RCP2.6\_binary

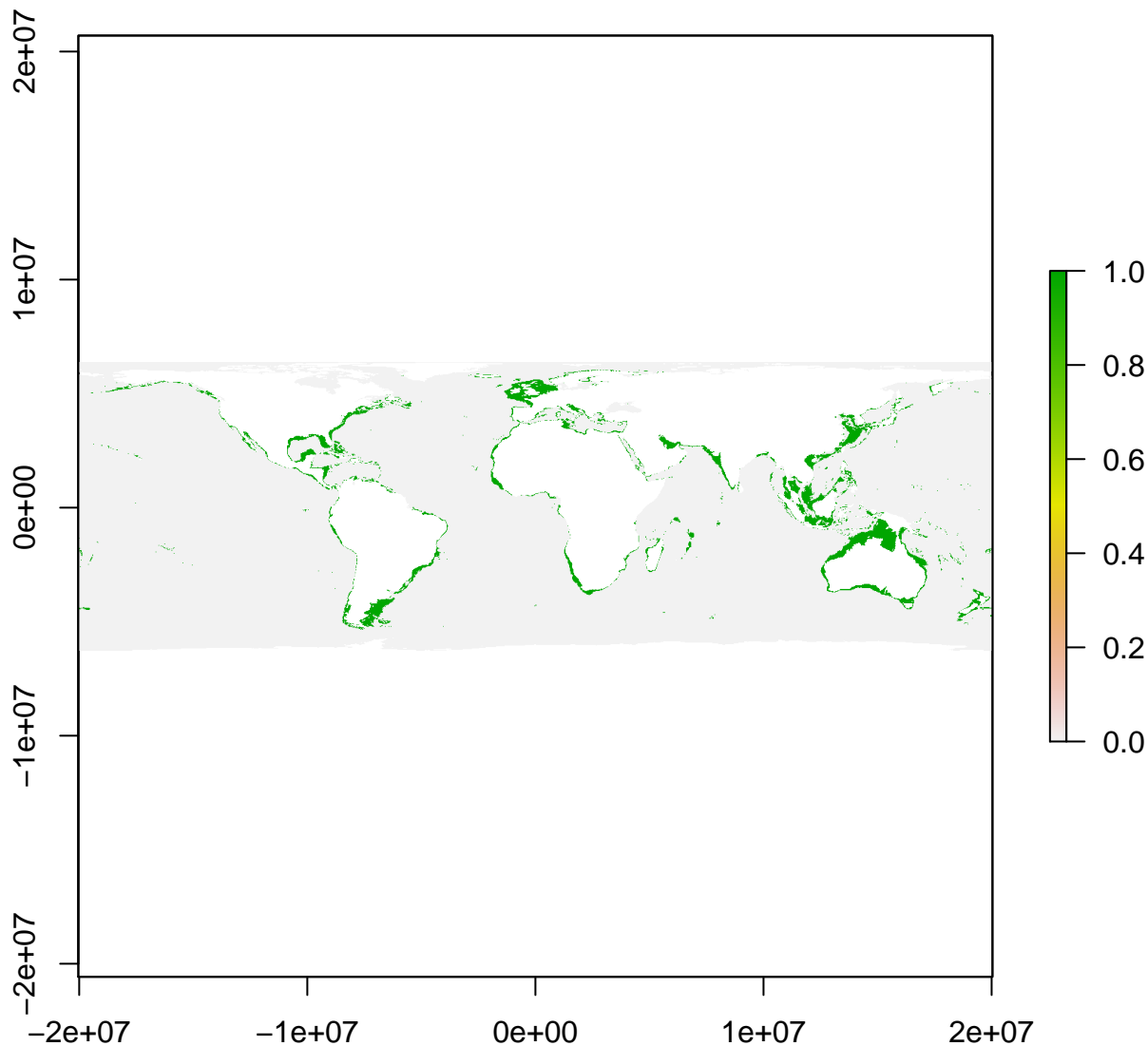

Supplement: Supplementary file 1 [file biology-11-01424-s001.zip › Maps/Binary/g_catenatum/2100_RCP2.6_binary.pdf]

# Gymnodinium\_catenatum\_2100\_RCP4.5\_binary

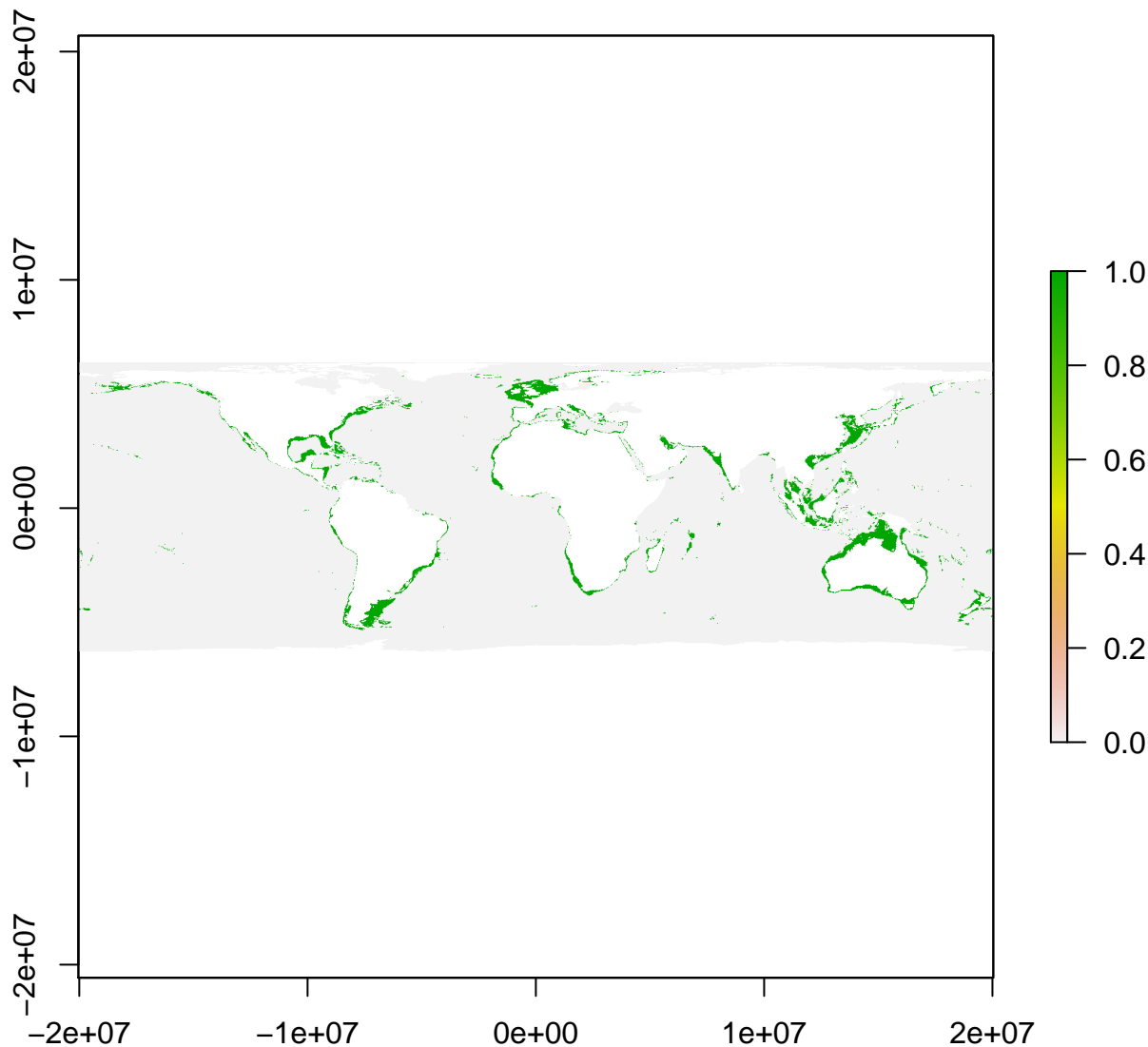

Supplement: Supplementary file 1 [file biology-11-01424-s001.zip › Maps/Binary/g_catenatum/2100_RCP4.5_binary.pdf]

# Gymnodinium\_catenatum\_2100\_RCP6.0\_binary

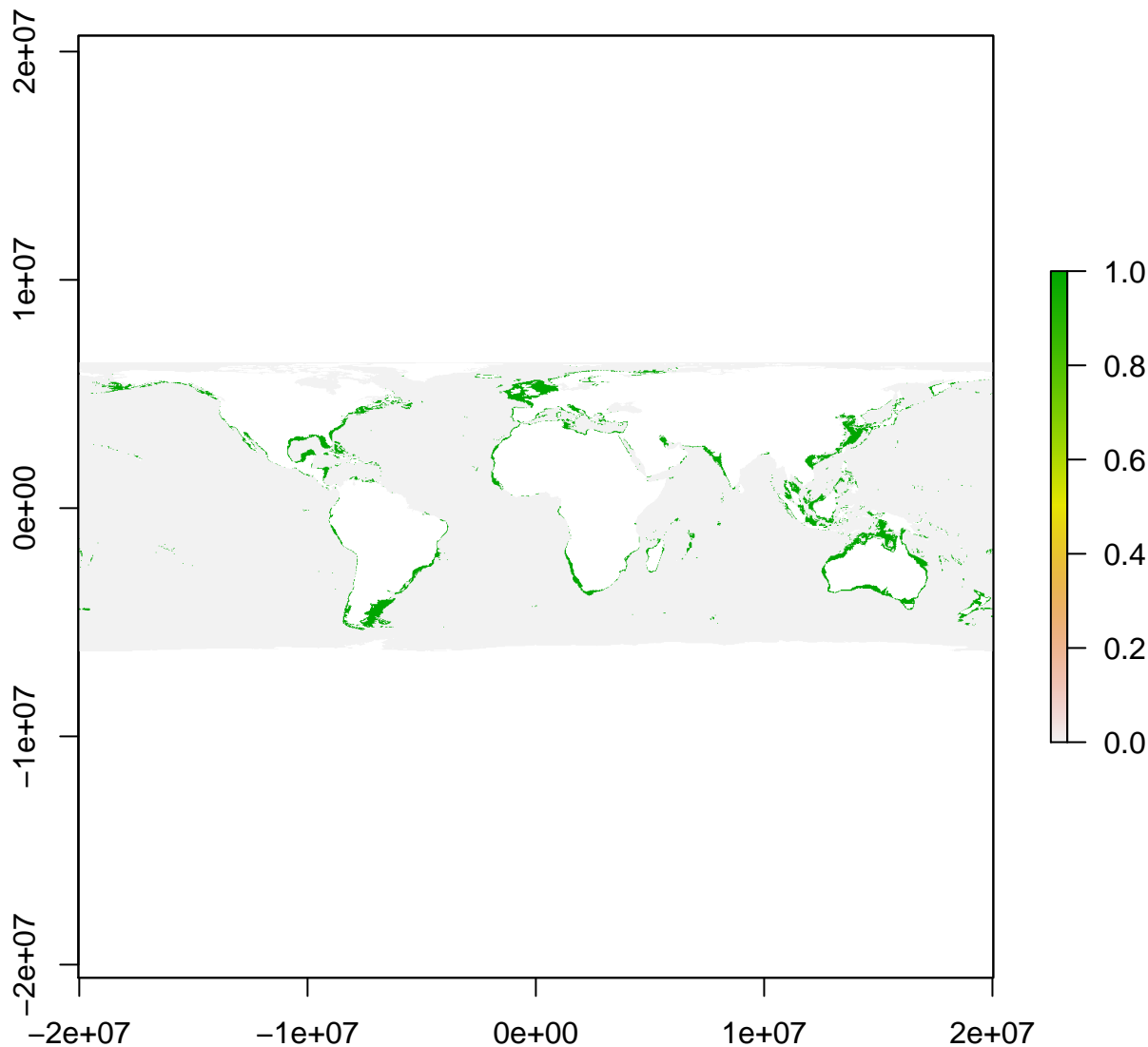

Supplement: Supplementary file 1 [file biology-11-01424-s001.zip › Maps/Binary/g_catenatum/2100_RCP6.0_binary.pdf]

# Gymnodinium\_catenatum\_2100\_RCP8.5\_binary

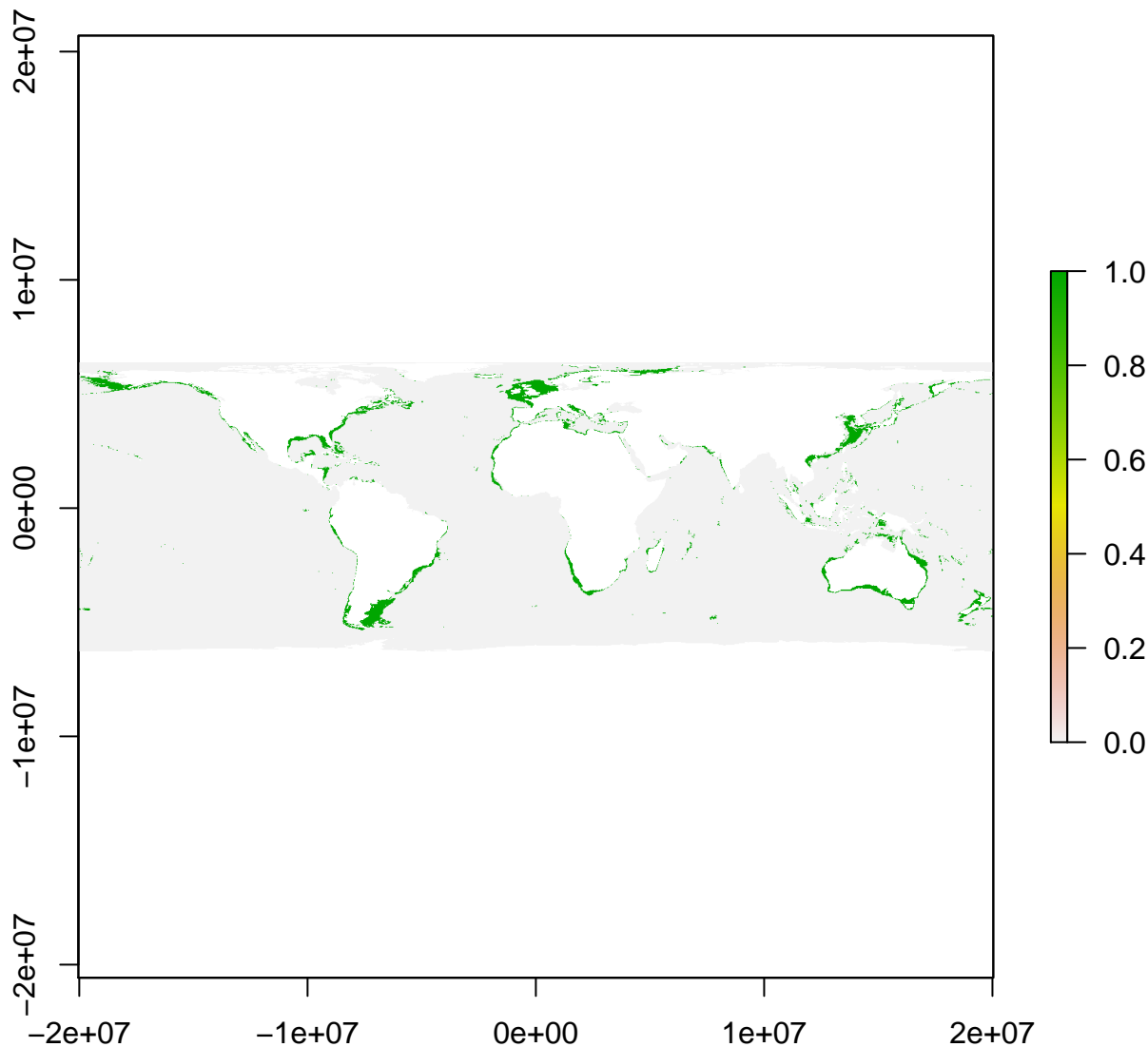

Supplement: Supplementary file 1 [file biology-11-01424-s001.zip › Maps/Binary/g_catenatum/2100_RCP8.5_binary.pdf]

# Gymnodinium\_catenatum\_present\_binary

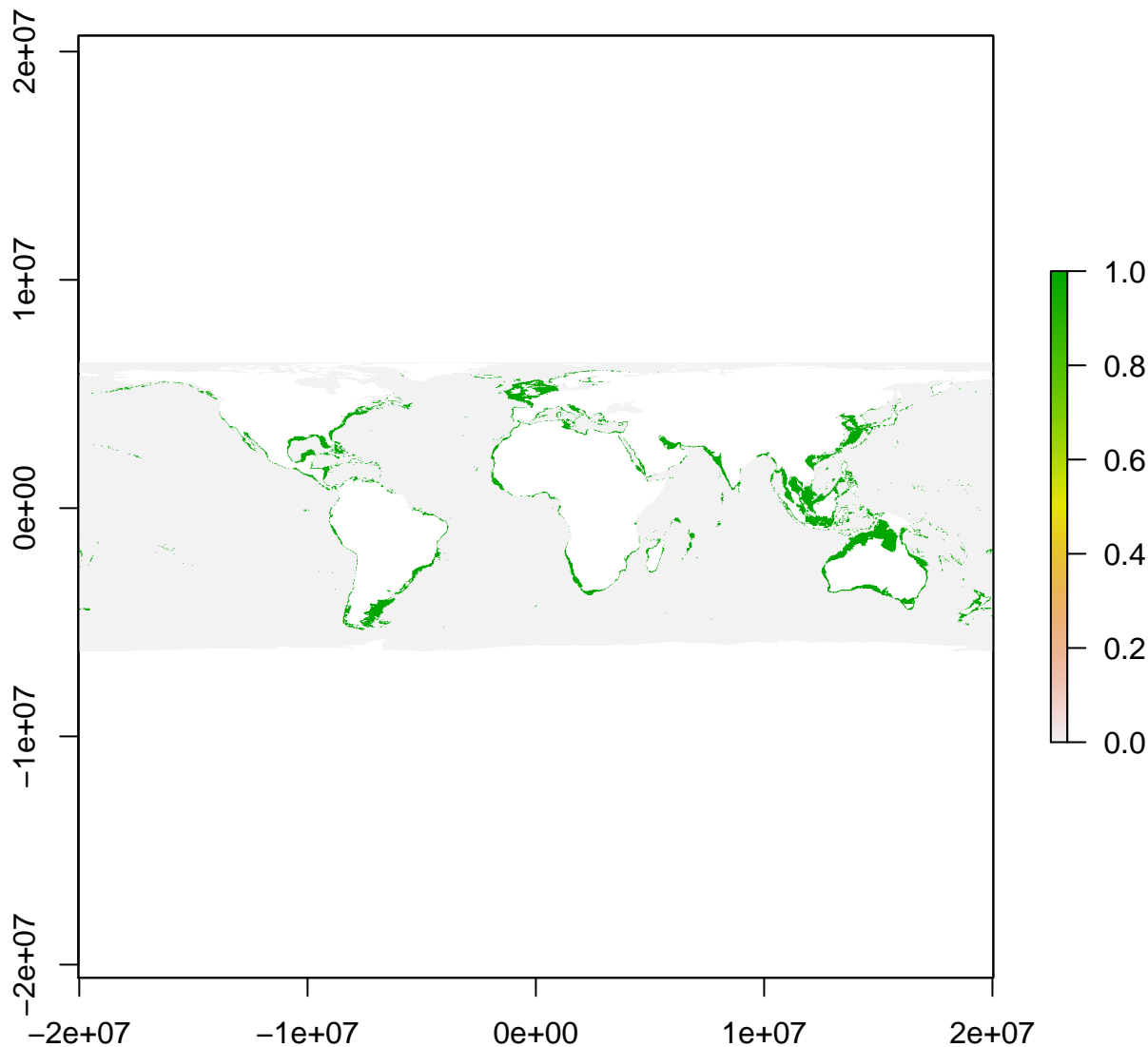

Supplement: Supplementary file 1 [file biology-11-01424-s001.zip › Maps/Binary/g_catenatum/present_binary.pdf]

# Alexandrium\_catenella\_2050\_RCP2.6

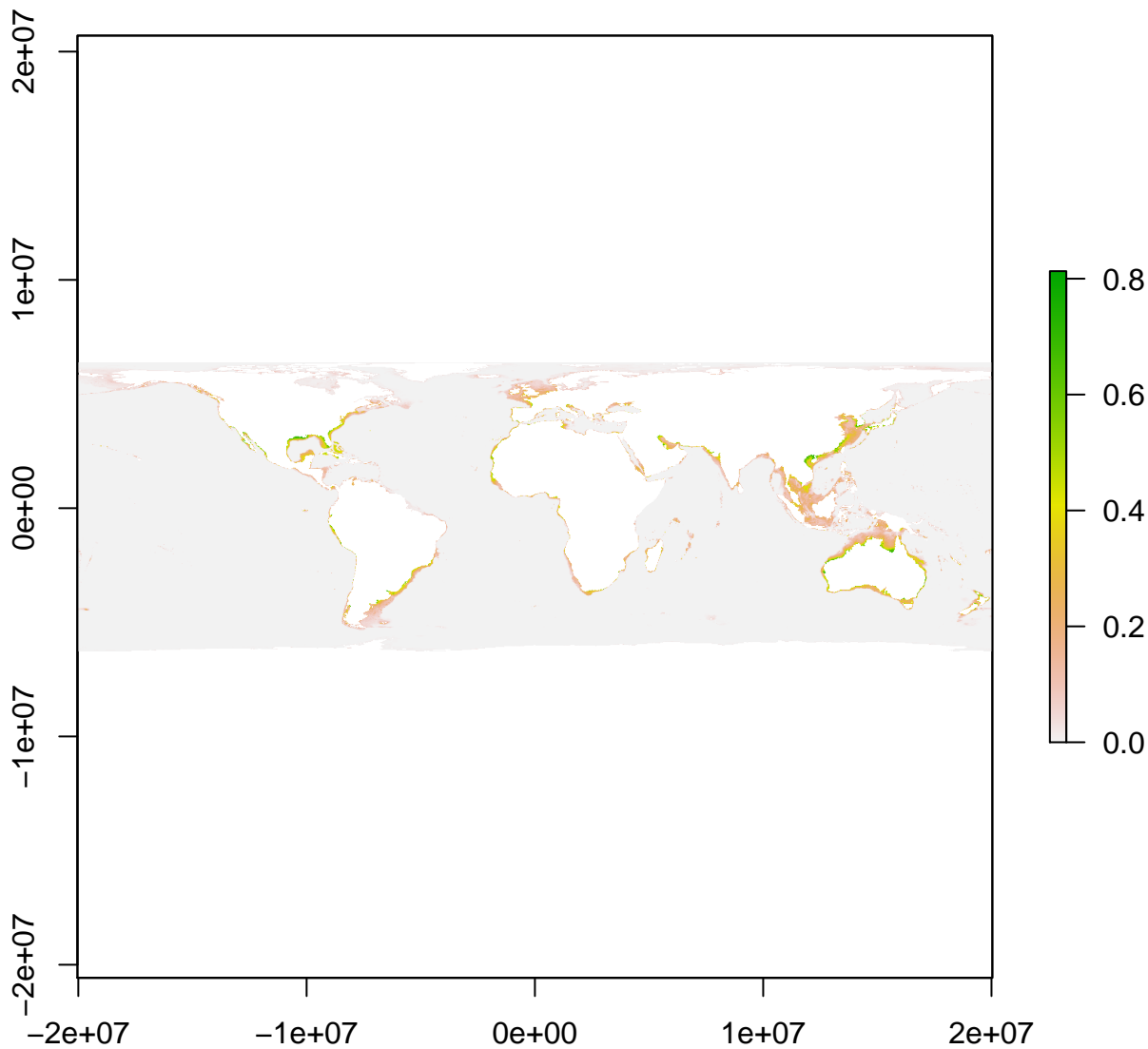

Supplement: Supplementary file 1 [file biology-11-01424-s001.zip › Maps/Ensemble/a_catenella/2050_RCP2.6_ensembled.pdf]

# Alexandrium\_catenella\_2050\_RCP4.5

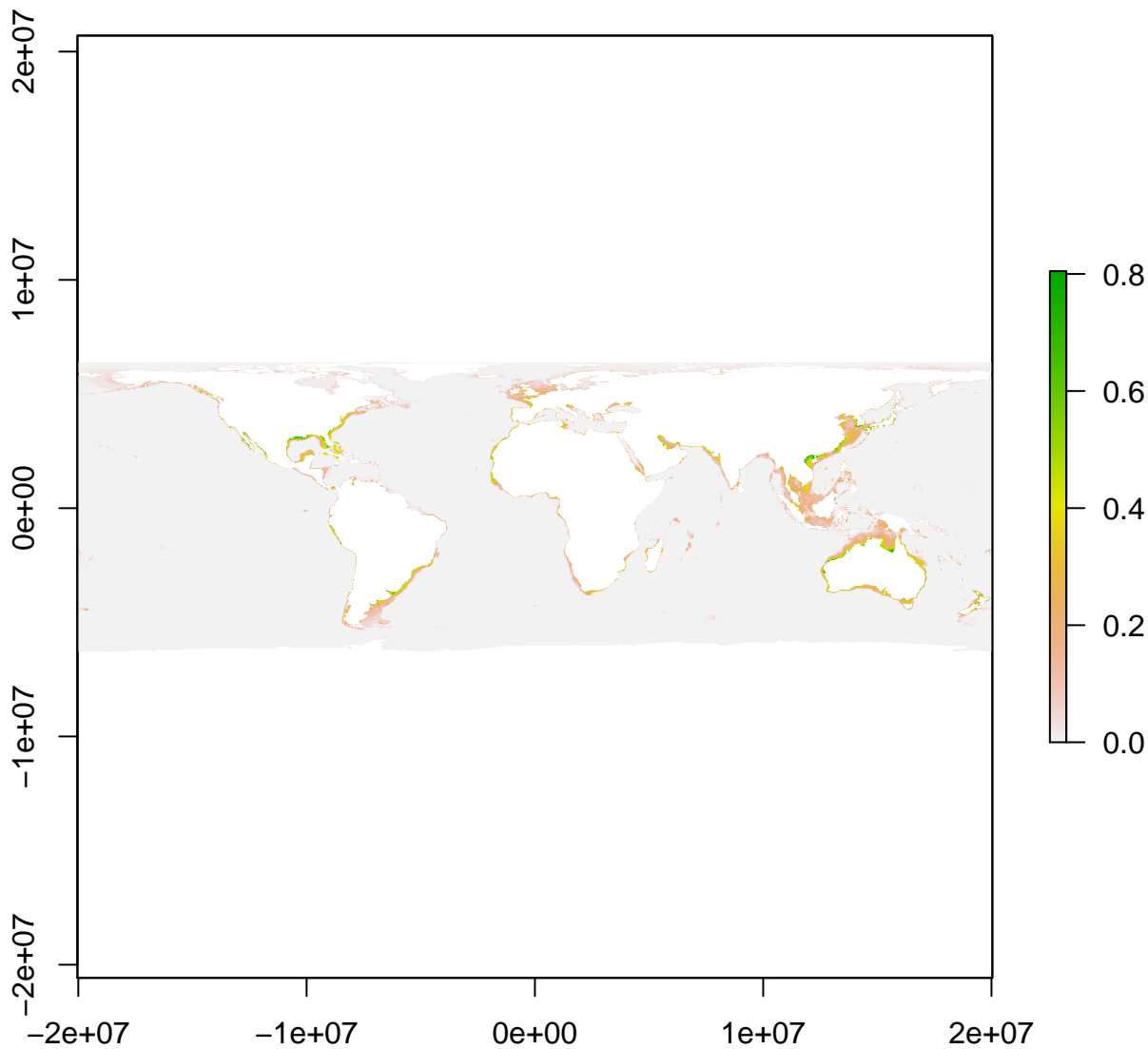

Supplement: Supplementary file 1 [file biology-11-01424-s001.zip › Maps/Ensemble/a_catenella/2050_RCP4.5_ensembled.pdf]

# Alexandrium\_catenella\_2050\_RCP6.0

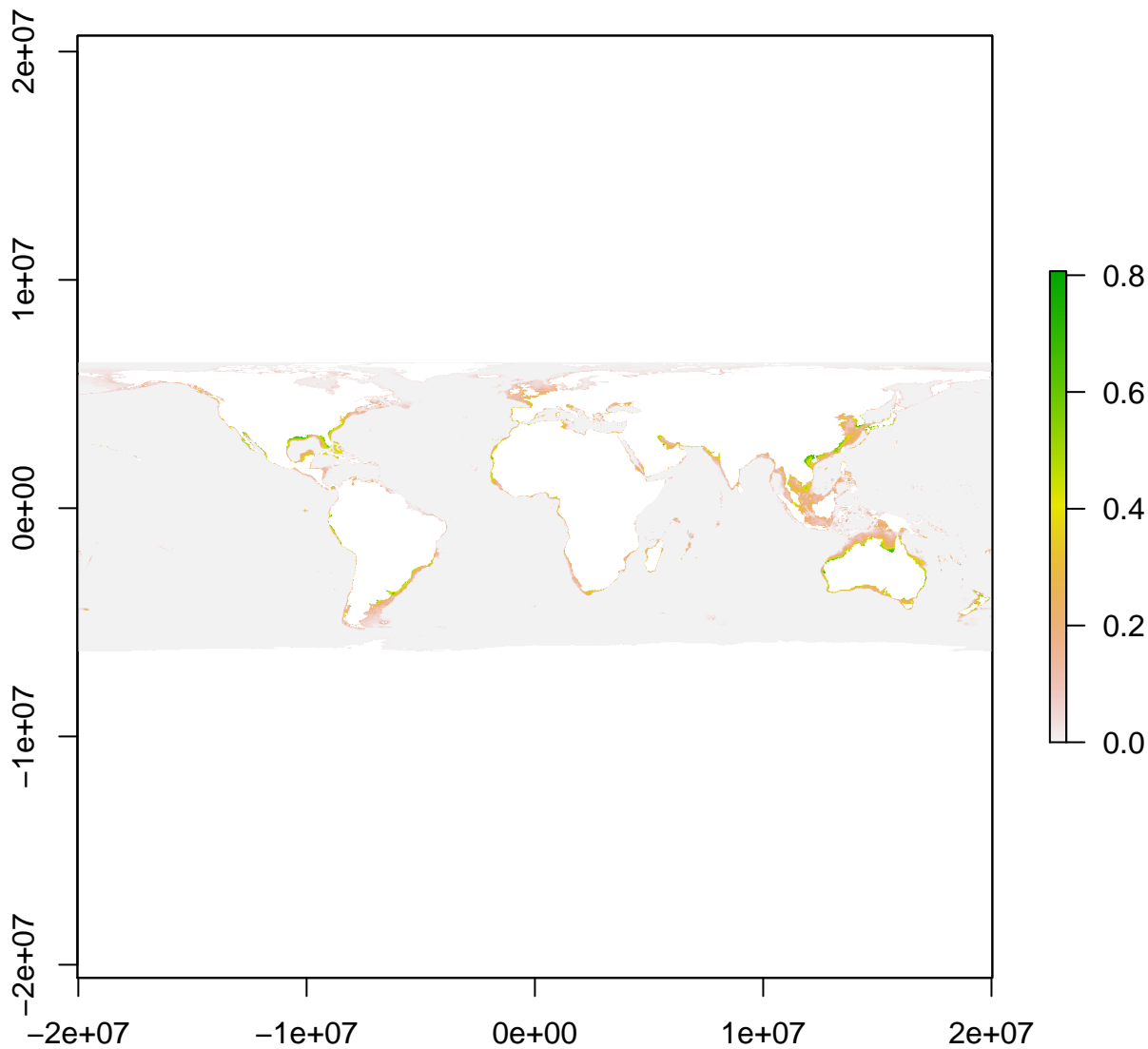

Supplement: Supplementary file 1 [file biology-11-01424-s001.zip › Maps/Ensemble/a_catenella/2050_RCP6.0_ensembled.pdf]

# Alexandrium\_catenella\_2050\_RCP8.5

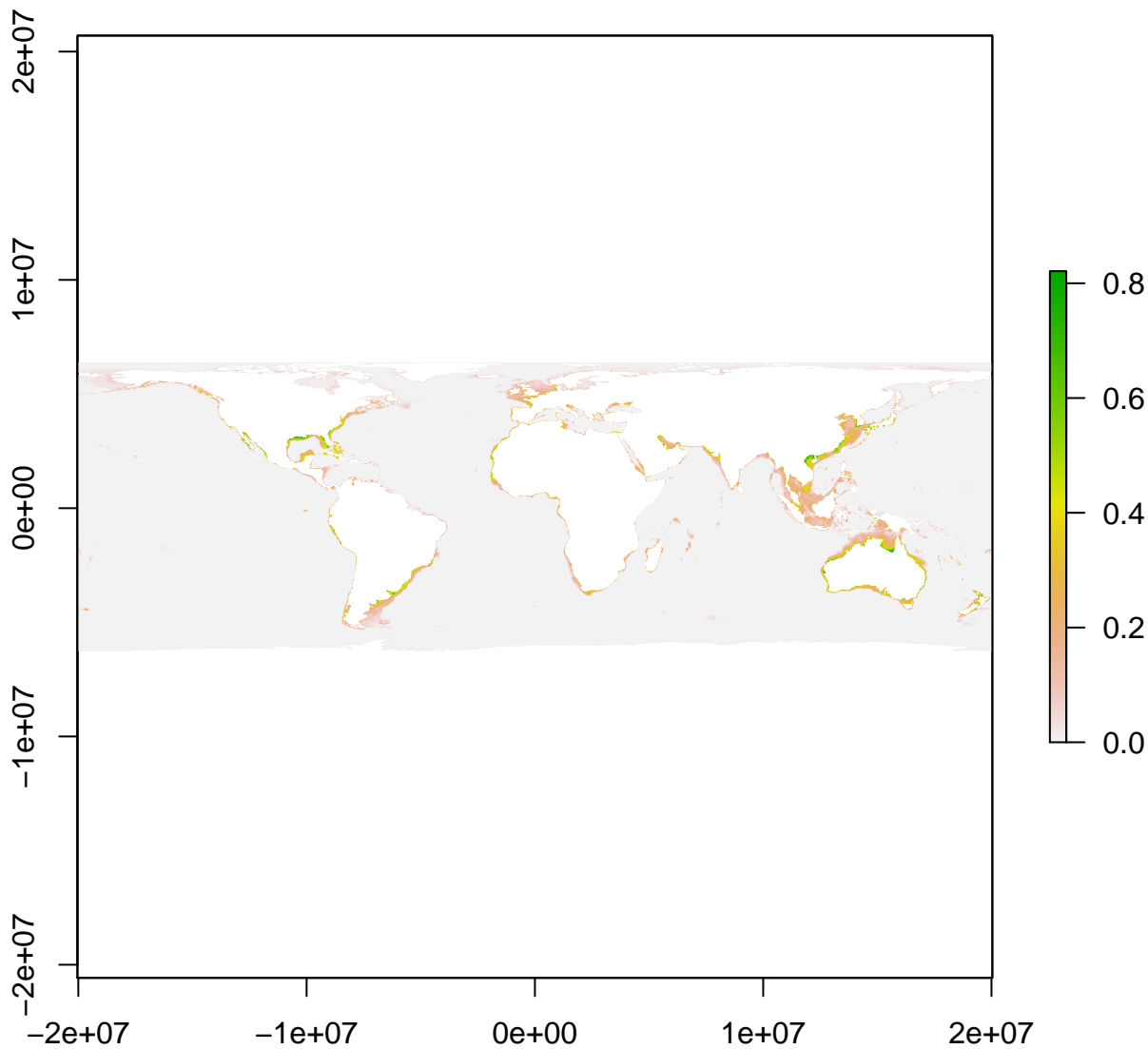

Supplement: Supplementary file 1 [file biology-11-01424-s001.zip › Maps/Ensemble/a_catenella/2050_RCP8.5_ensembled.pdf]

# Alexandrium\_catenella\_2100\_RCP2.6

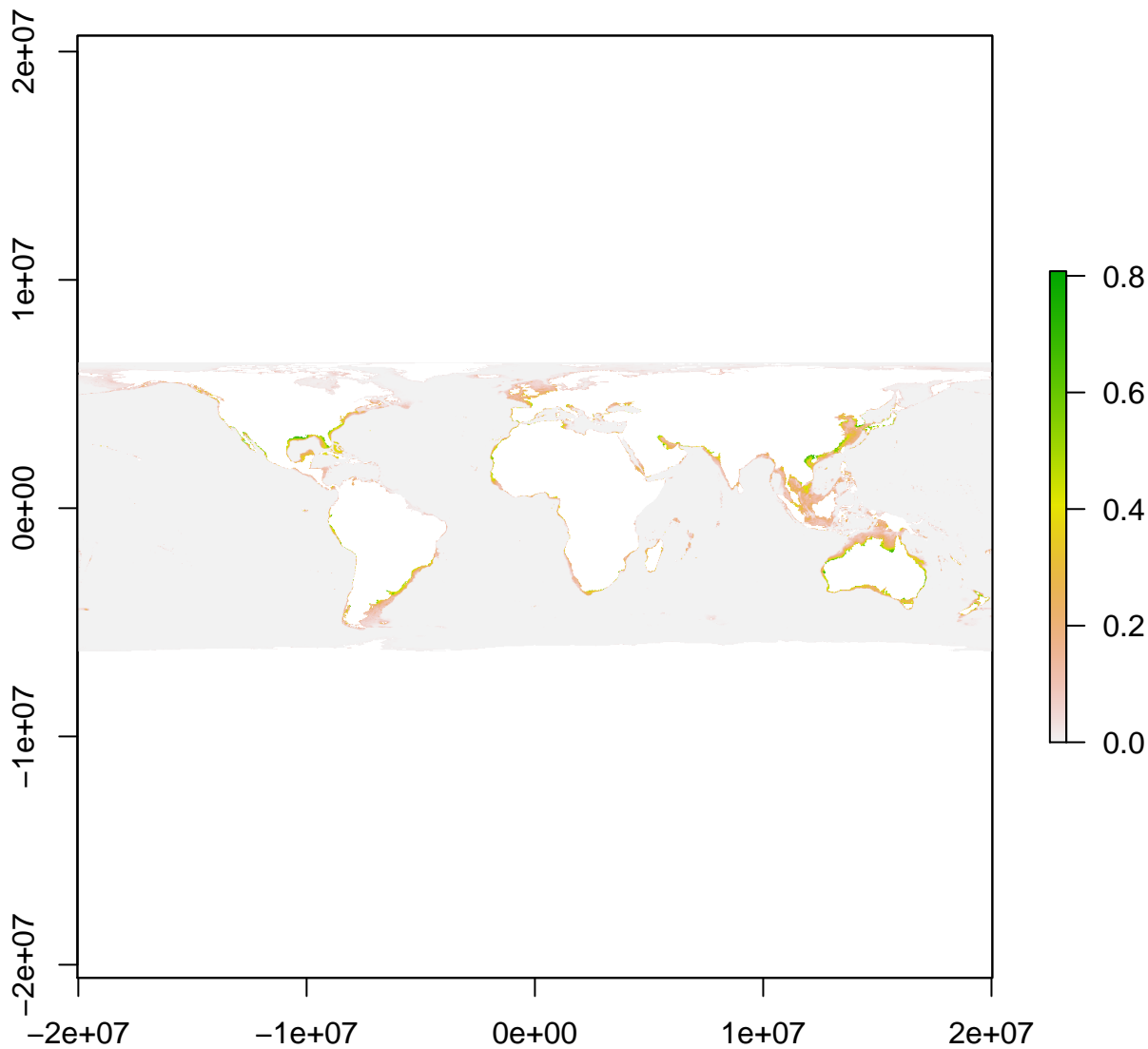

Supplement: Supplementary file 1 [file biology-11-01424-s001.zip › Maps/Ensemble/a_catenella/2100_RCP2.6_ensembled.pdf]

# Alexandrium\_catenella\_2100\_RCP4.5

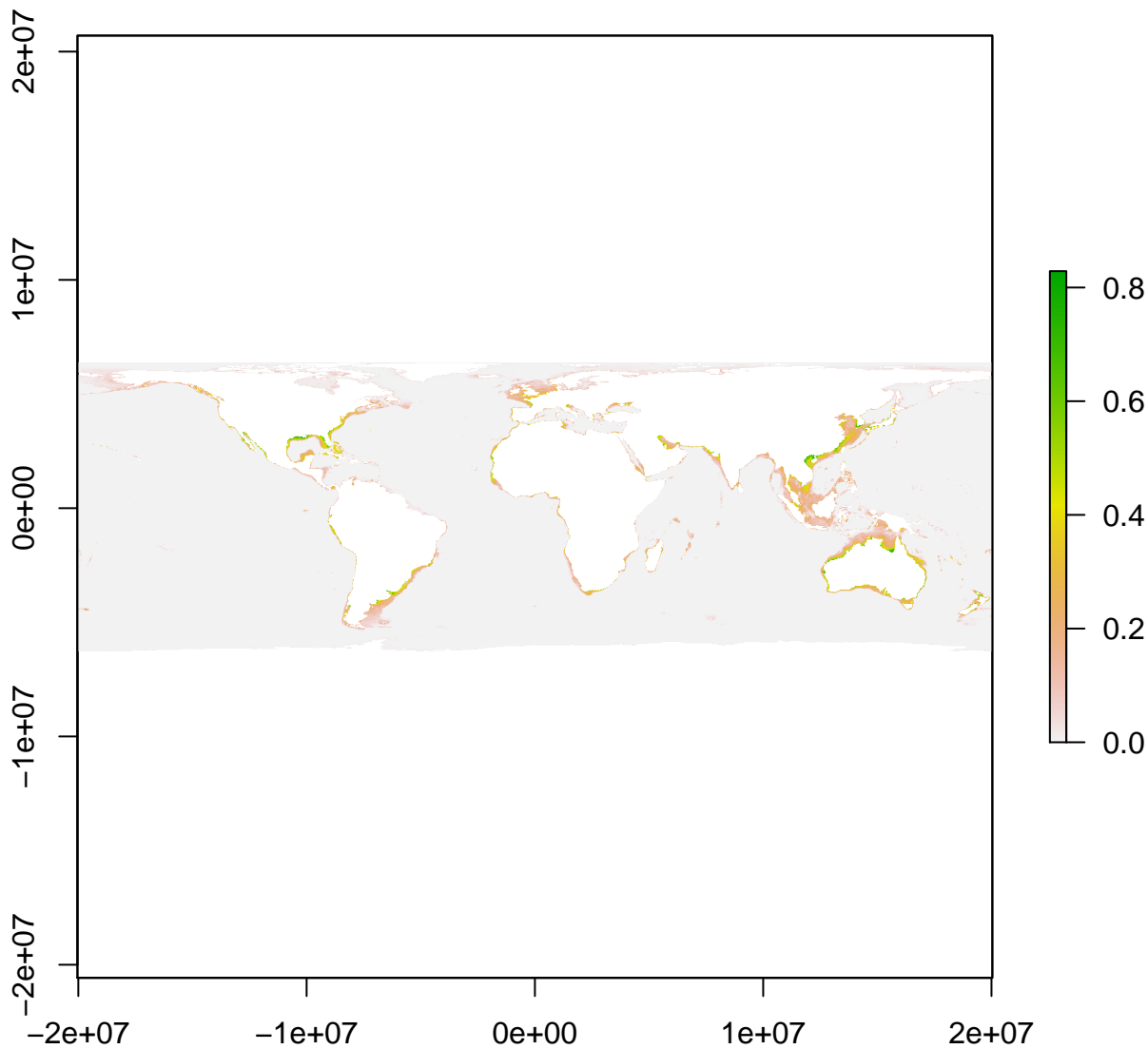

Supplement: Supplementary file 1 [file biology-11-01424-s001.zip › Maps/Ensemble/a_catenella/2100_RCP4.5_ensembled.pdf]

# Alexandrium\_catenella\_2100\_RCP6.0

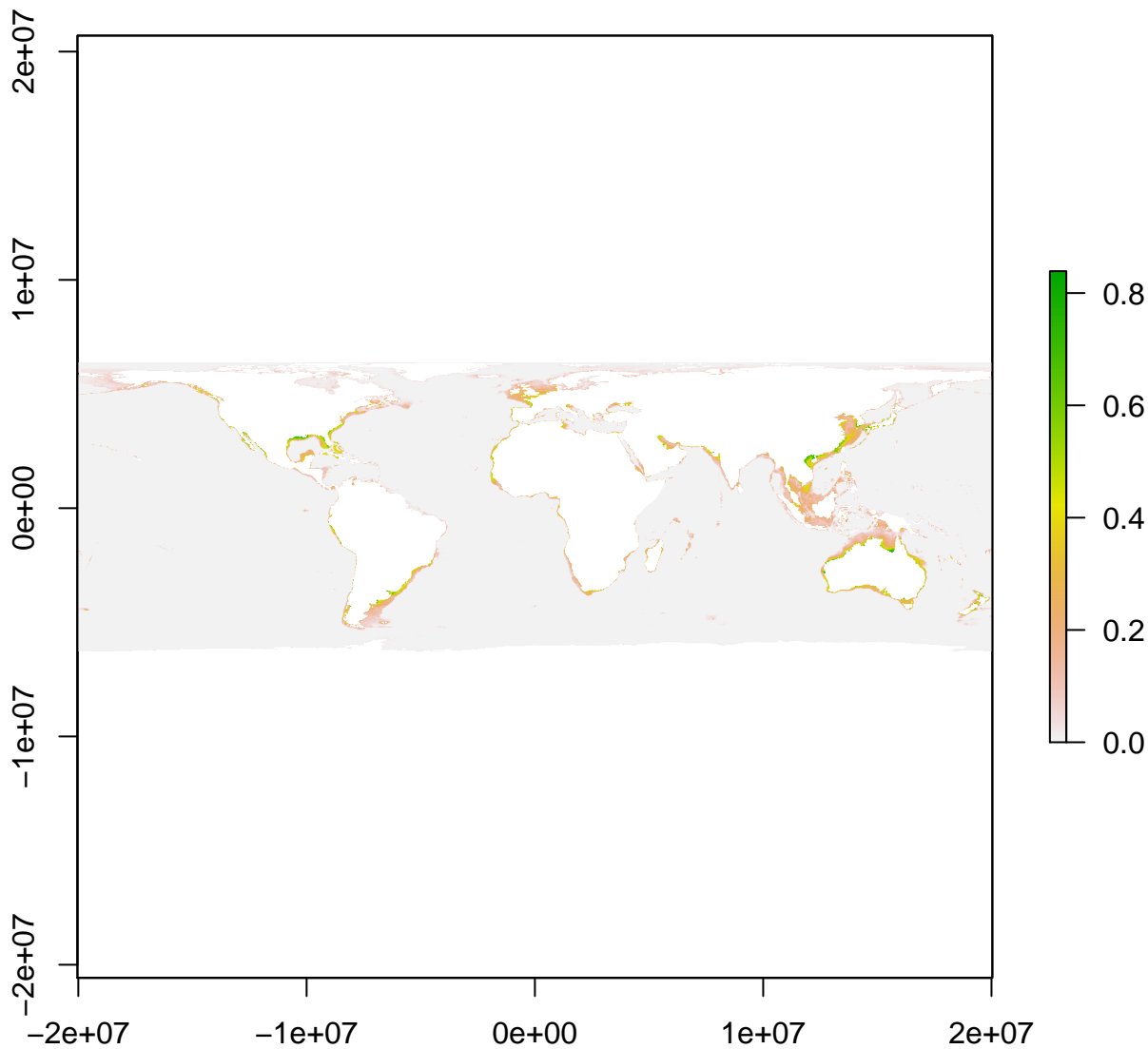

Supplement: Supplementary file 1 [file biology-11-01424-s001.zip › Maps/Ensemble/a_catenella/2100_RCP6.0_ensembled.pdf]

# Alexandrium\_catenella\_2100\_RCP8.5

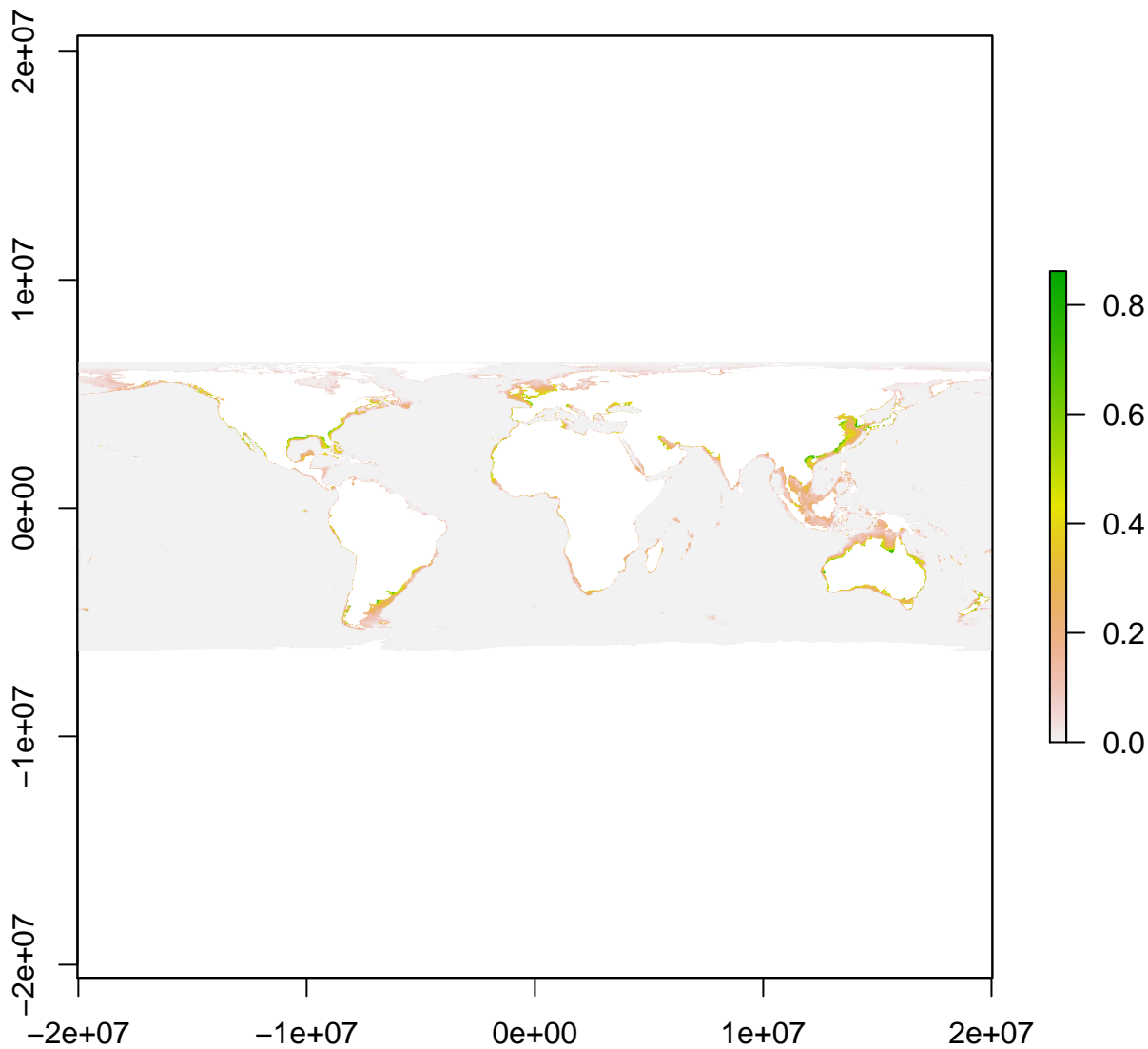

Supplement: Supplementary file 1 [file biology-11-01424-s001.zip › Maps/Ensemble/a_catenella/2100_RCP8.5_ensembled.pdf]

# Alexandrium\_catenella\_present

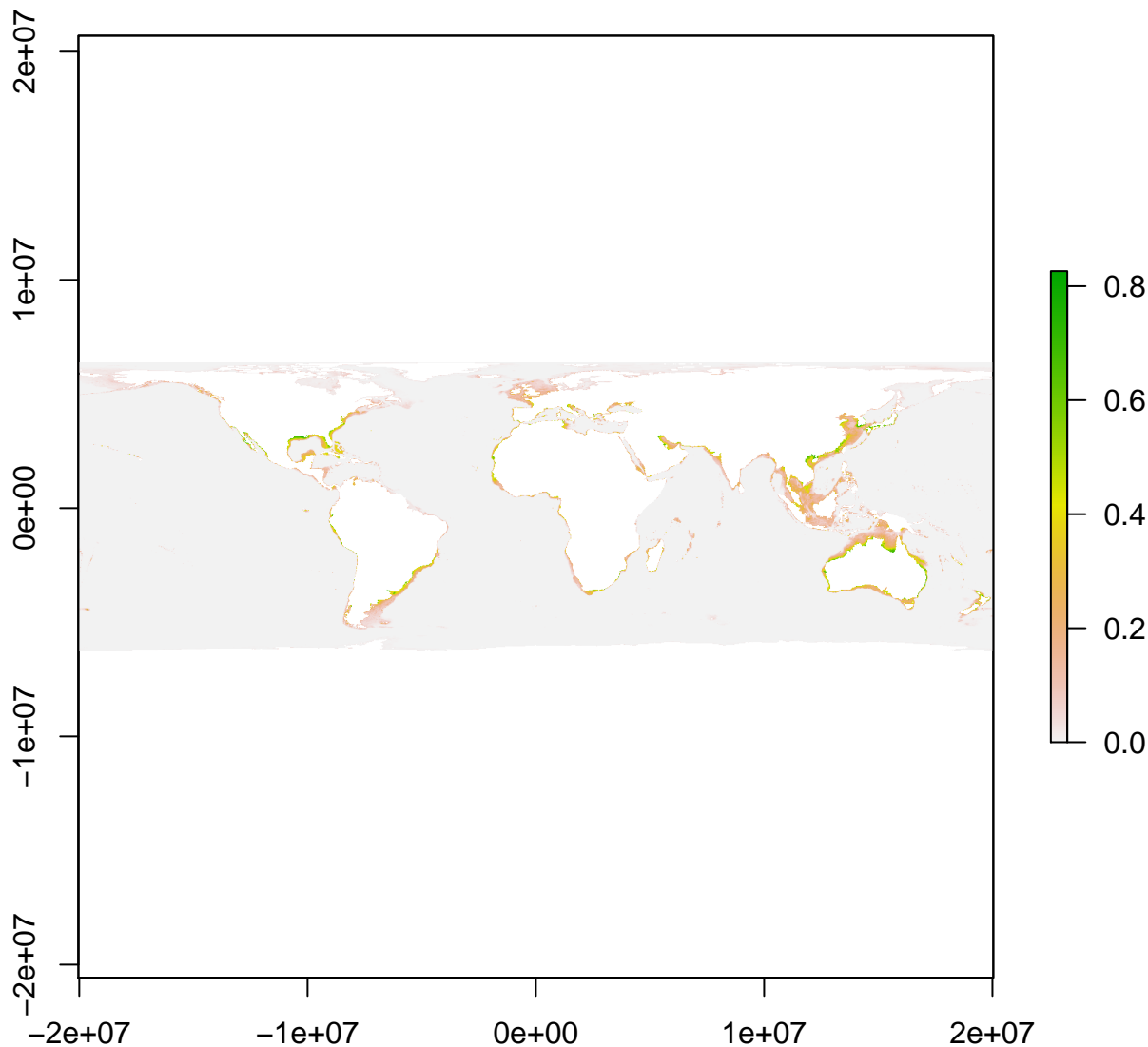

Supplement: Supplementary file 1 [file biology-11-01424-s001.zip › Maps/Ensemble/a_catenella/present_ensembled.pdf]

# Alexandrium\_minutum\_2050\_RCP2.6

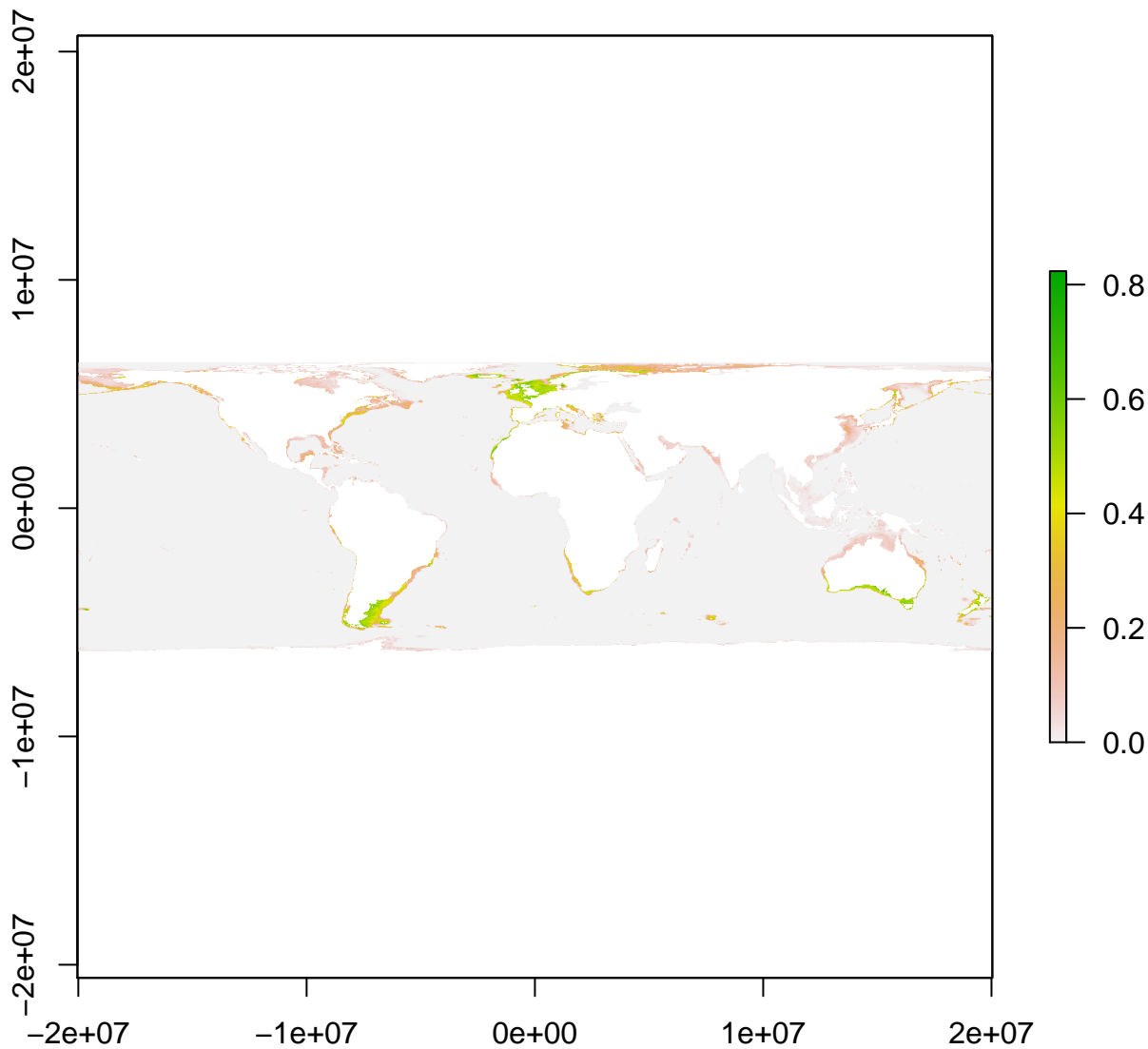

Supplement: Supplementary file 1 [file biology-11-01424-s001.zip › Maps/Ensemble/a_minutum/2050_RCP2.6_ensembled.pdf]

# Alexandrium\_minutum\_2050\_RCP4.5

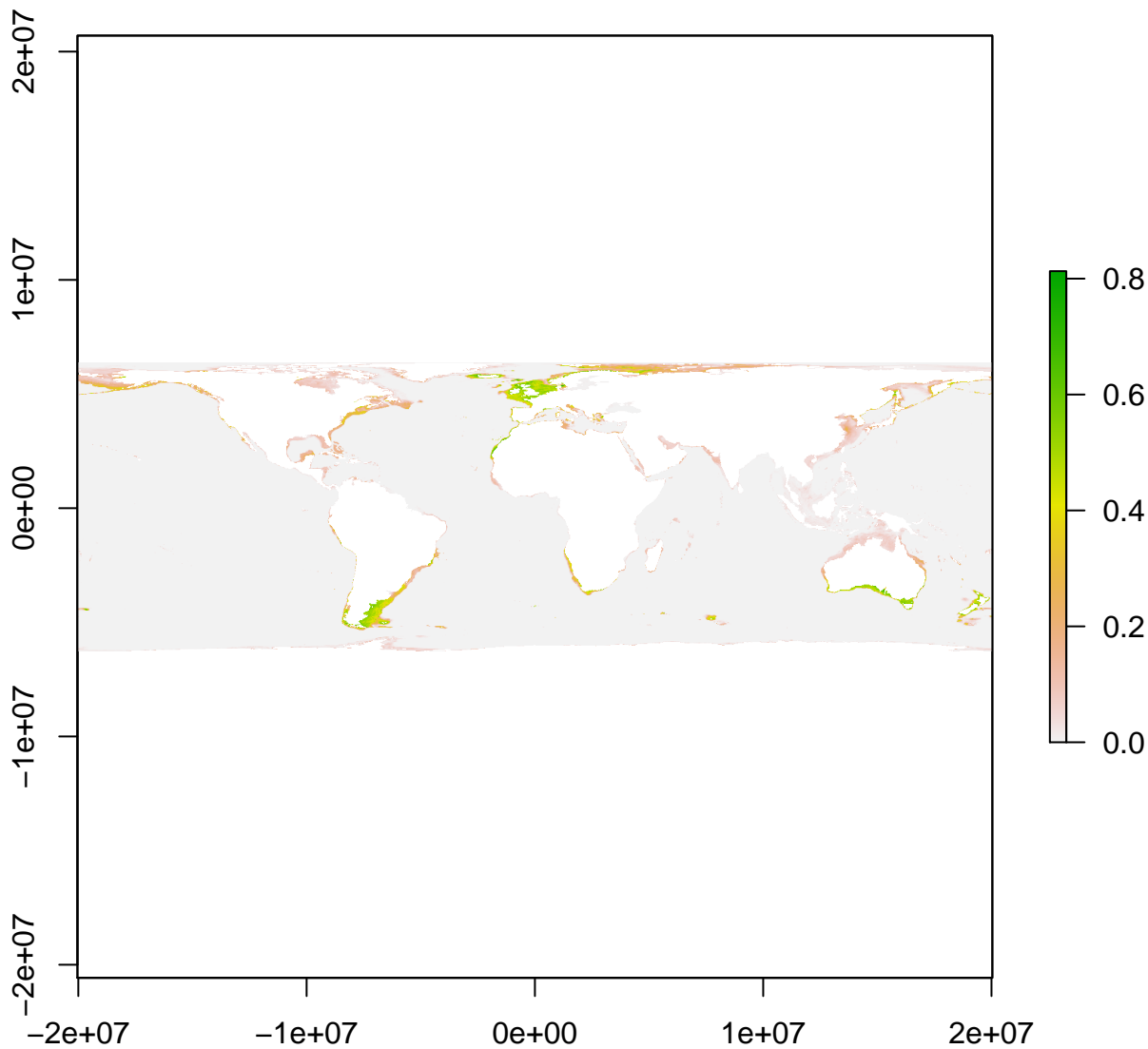

Supplement: Supplementary file 1 [file biology-11-01424-s001.zip › Maps/Ensemble/a_minutum/2050_RCP4.5_ensembled.pdf]

# Alexandrium\_minutum\_2050\_RCP6.0

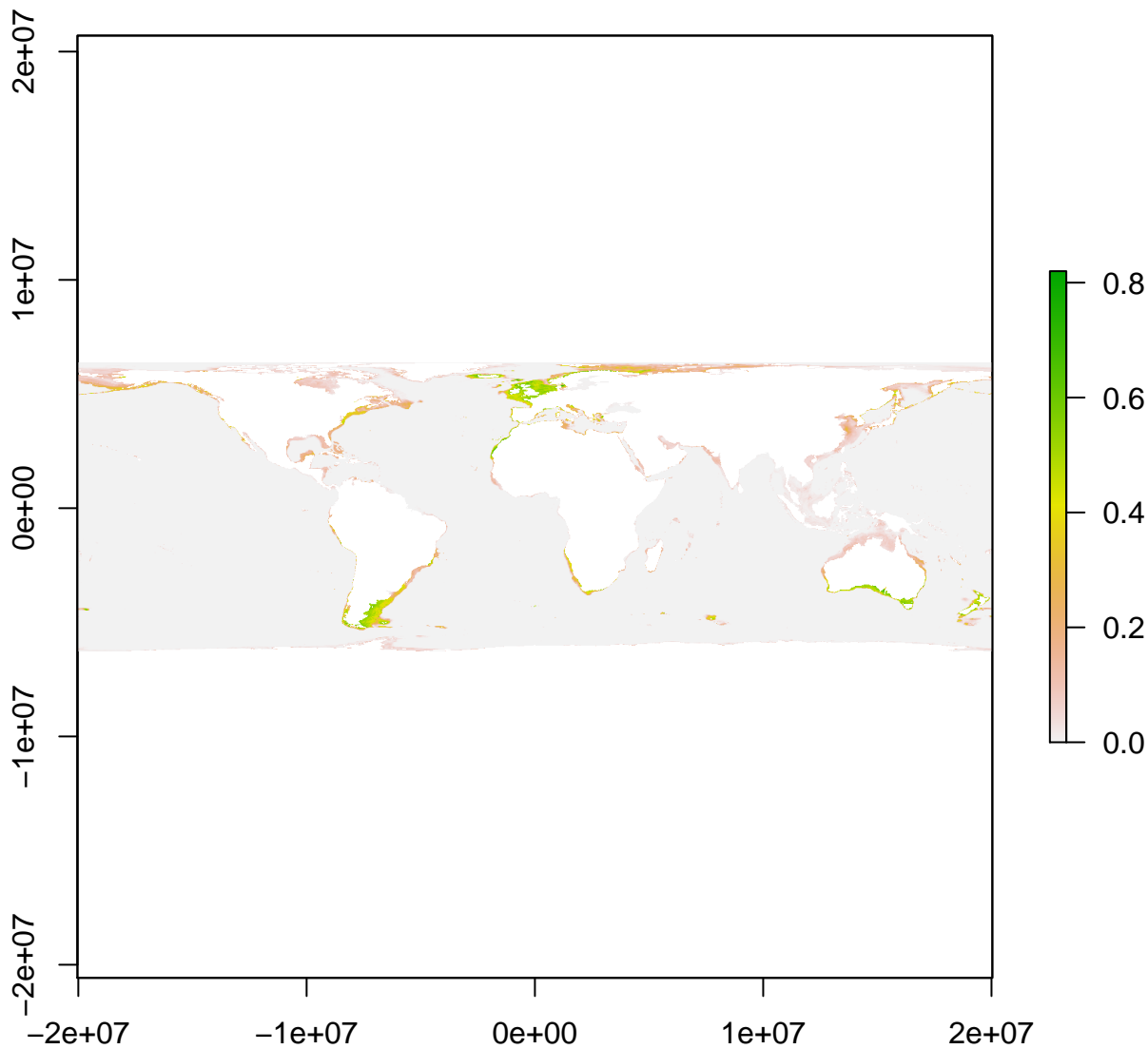

Supplement: Supplementary file 1 [file biology-11-01424-s001.zip › Maps/Ensemble/a_minutum/2050_RCP6.0_ensembled.pdf]

# Alexandrium\_minutum\_2050\_RCP8.5

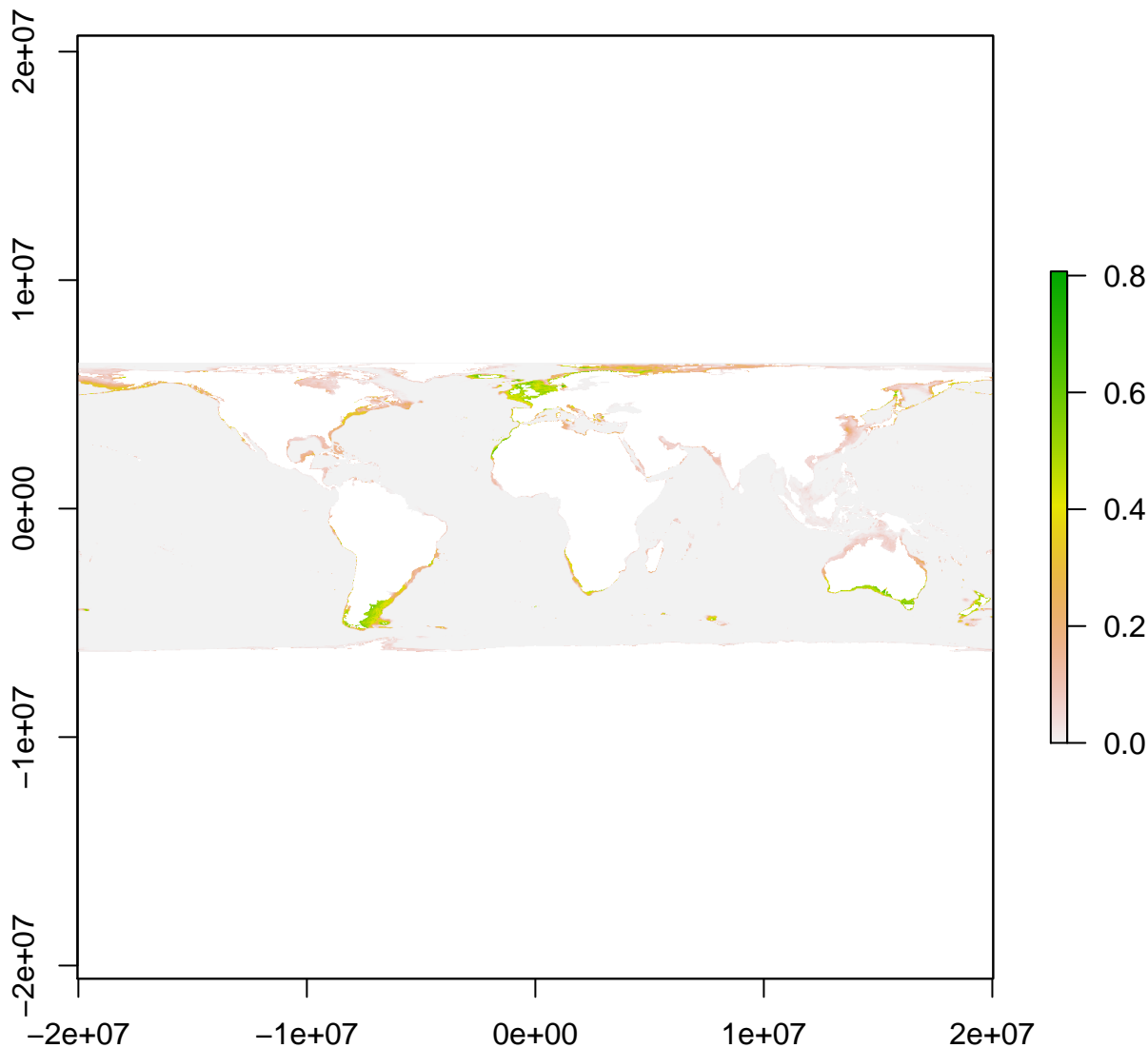

Supplement: Supplementary file 1 [file biology-11-01424-s001.zip › Maps/Ensemble/a_minutum/2050_RCP8.5_ensembled.pdf]

# Alexandrium\_minutum\_2100\_RCP2.6

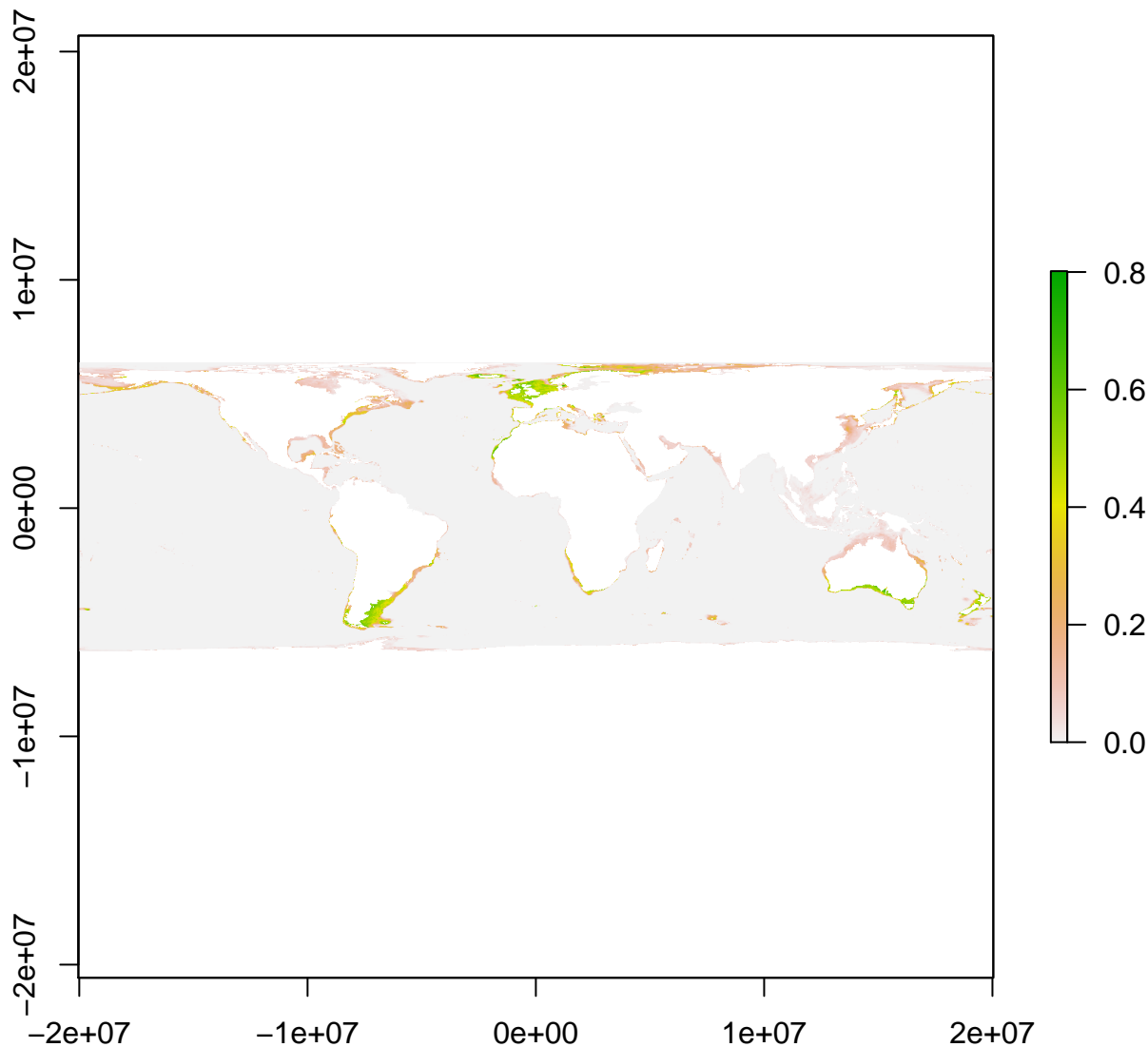

Supplement: Supplementary file 1 [file biology-11-01424-s001.zip › Maps/Ensemble/a_minutum/2100_RCP2.6_ensembled.pdf]

# Alexandrium\_minutum\_2100\_RCP4.5

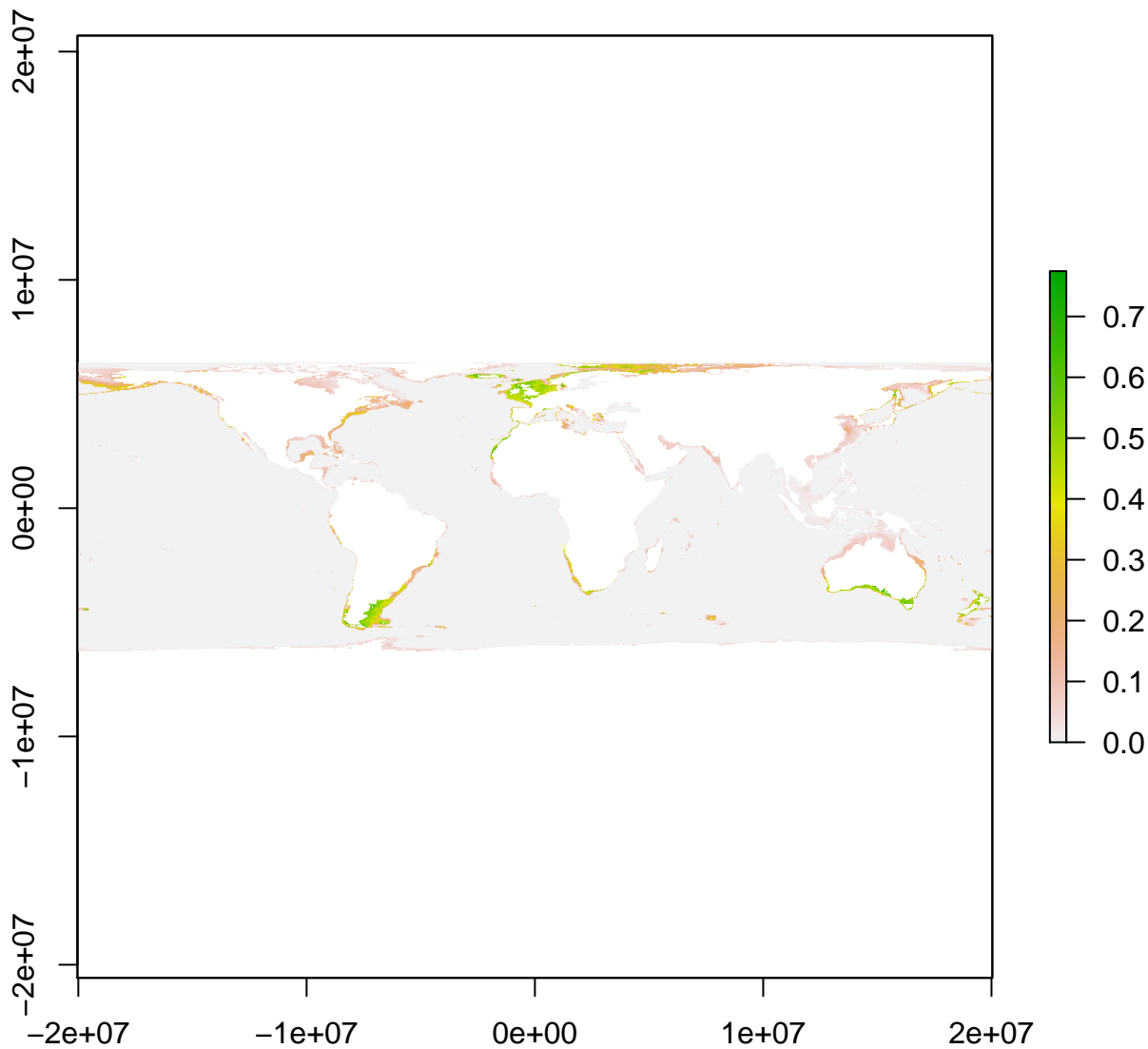

Supplement: Supplementary file 1 [file biology-11-01424-s001.zip › Maps/Ensemble/a_minutum/2100_RCP4.5_ensembled.pdf]

# Alexandrium\_minutum\_2100\_RCP6.0

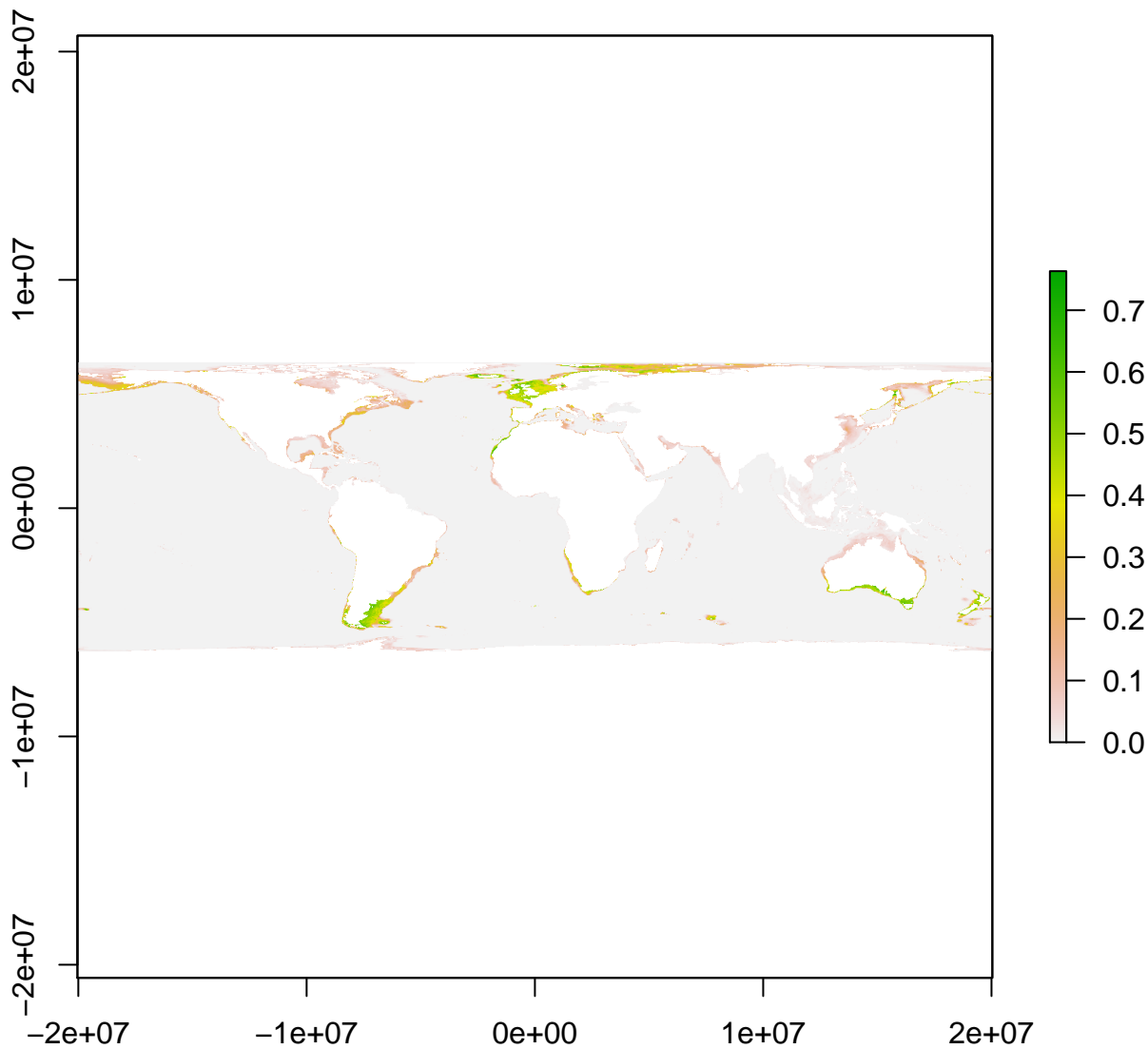

Supplement: Supplementary file 1 [file biology-11-01424-s001.zip › Maps/Ensemble/a_minutum/2100_RCP6.0_ensembled.pdf]

# Alexandrium\_minutum\_2100\_RCP8.5

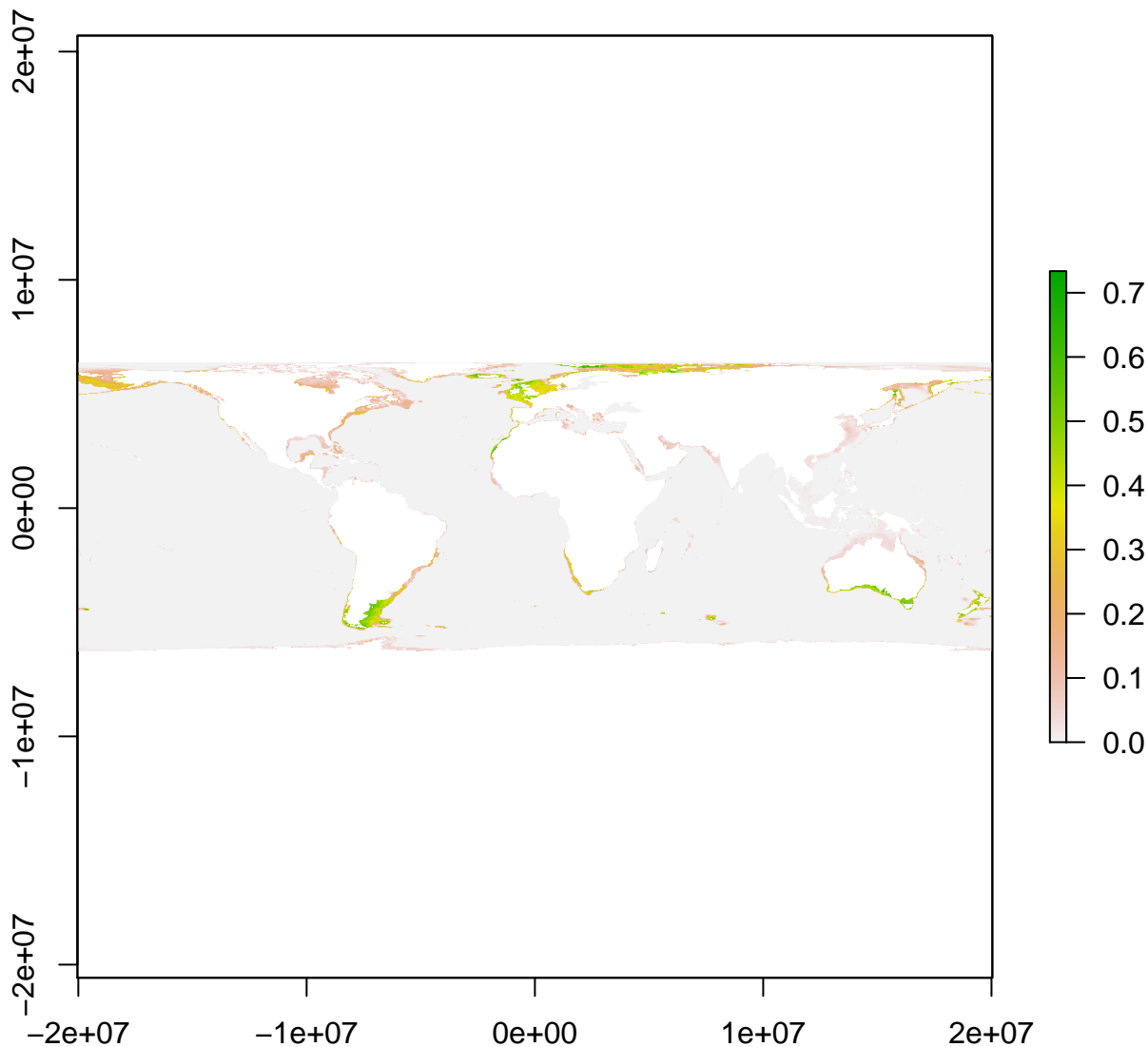

Supplement: Supplementary file 1 [file biology-11-01424-s001.zip › Maps/Ensemble/a_minutum/2100_RCP8.5_ensembled.pdf]

# Alexandrium\_minutum\_present

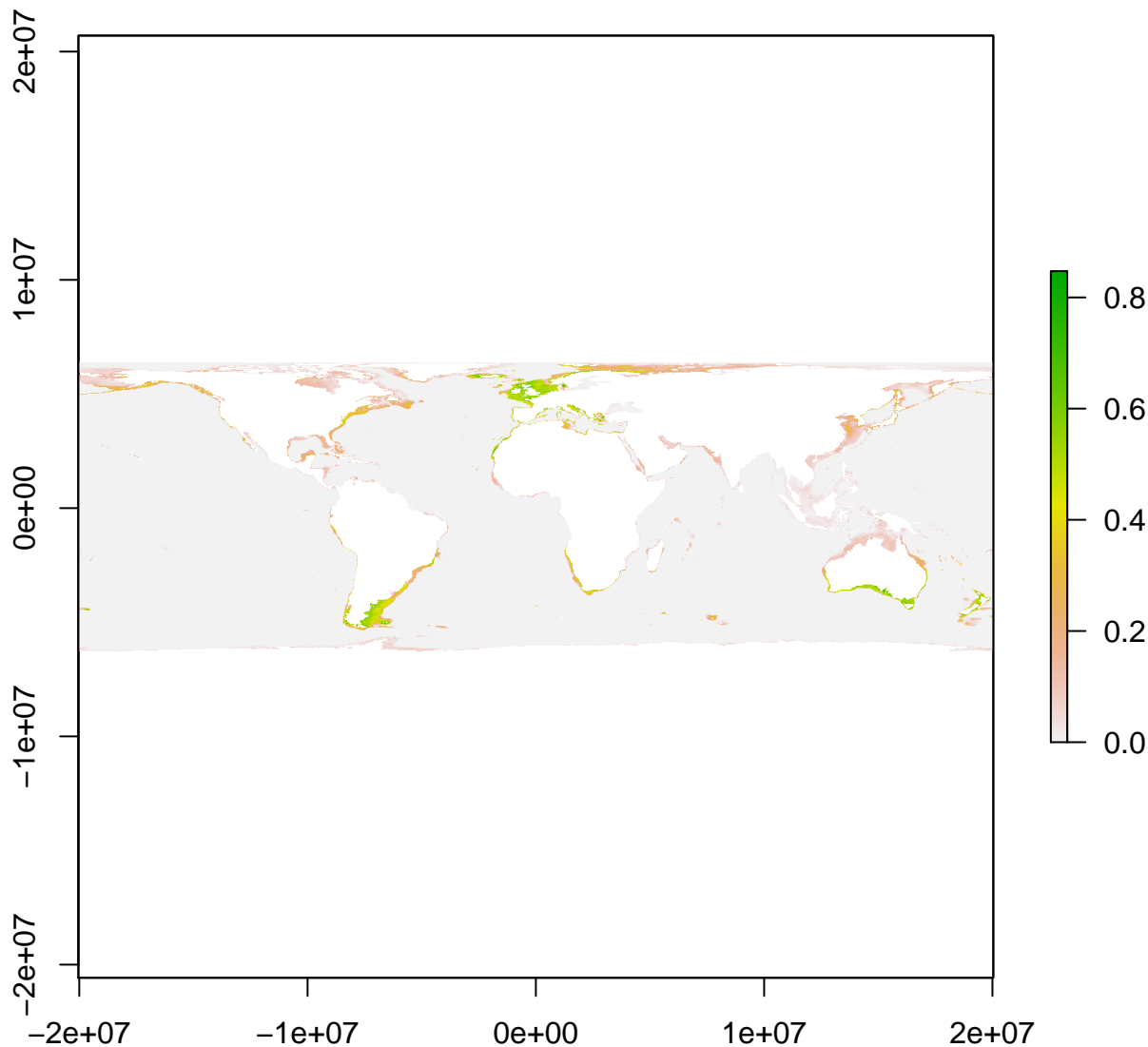

Supplement: Supplementary file 1 [file biology-11-01424-s001.zip › Maps/Ensemble/a_minutum/present_ensembled.pdf]

# Gymnodinium\_catenatum\_2050\_RCP2.6

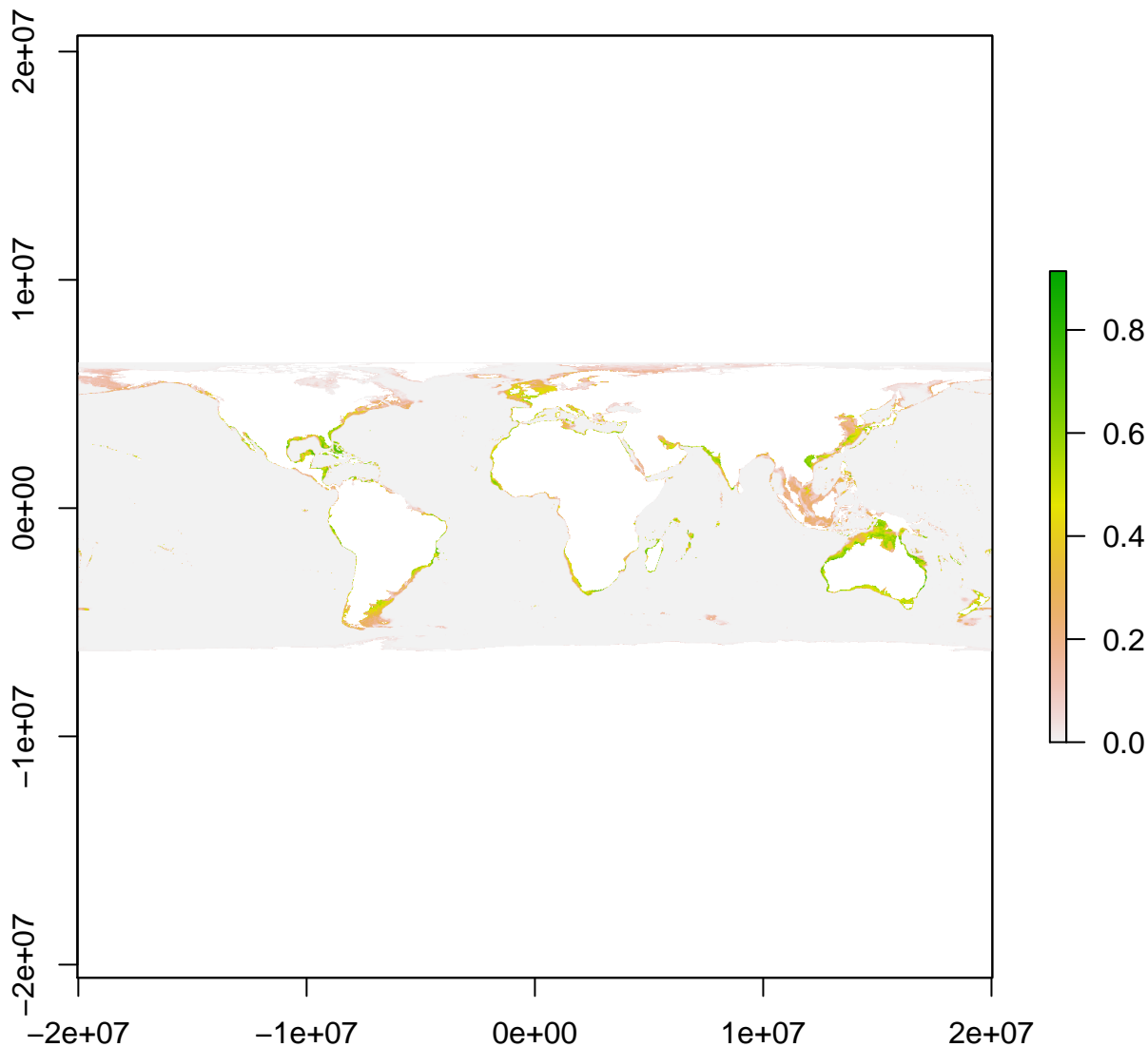

Supplement: Supplementary file 1 [file biology-11-01424-s001.zip › Maps/Ensemble/g_catenatum/2050_RCP2.6_ensembled.pdf]

# Gymnodinium\_catenatum\_2050\_RCP4.5

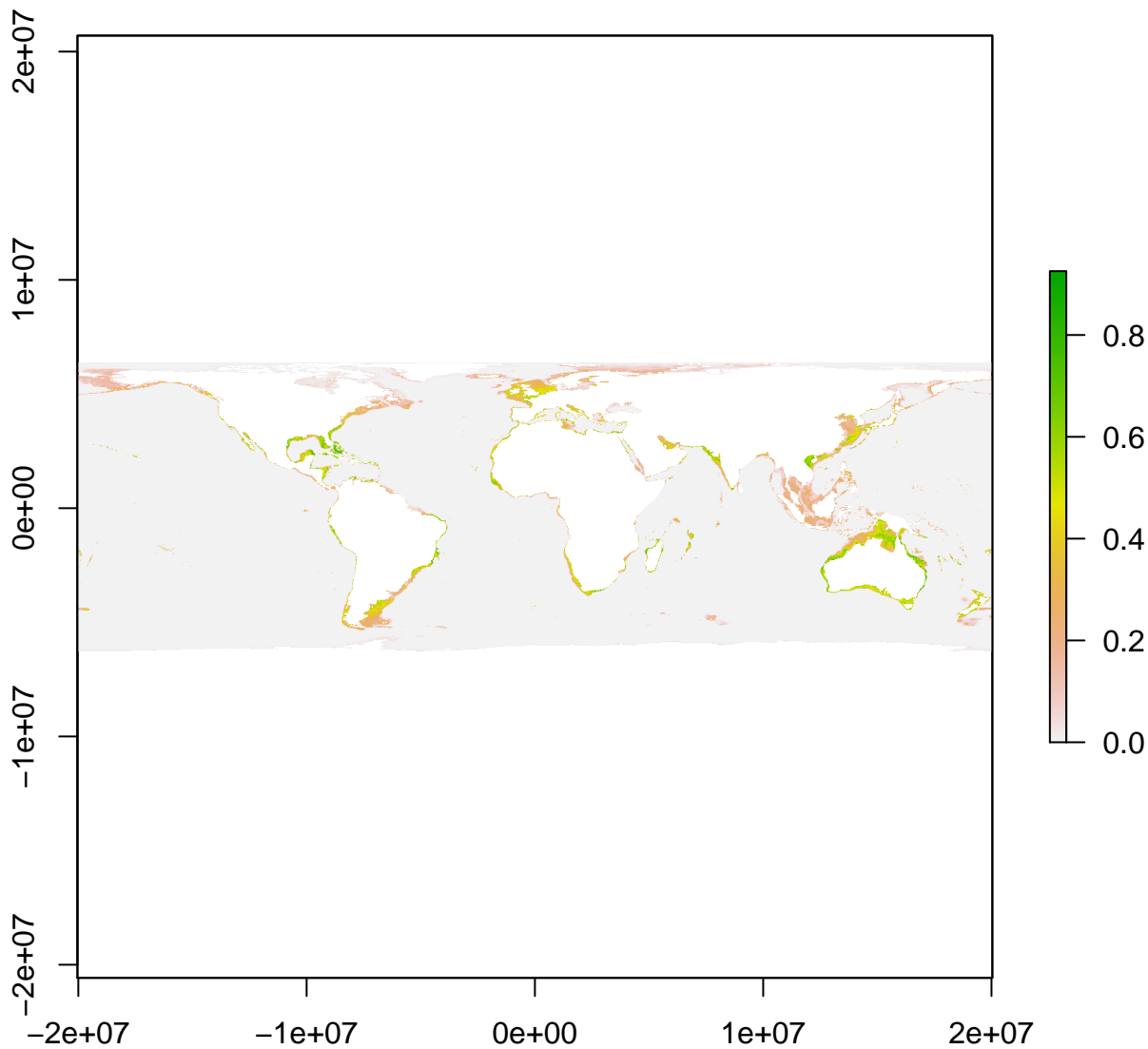

Supplement: Supplementary file 1 [file biology-11-01424-s001.zip › Maps/Ensemble/g_catenatum/2050_RCP4.5_ensembled.pdf]

# Gymnodinium\_catenatum\_2050\_RCP6.0

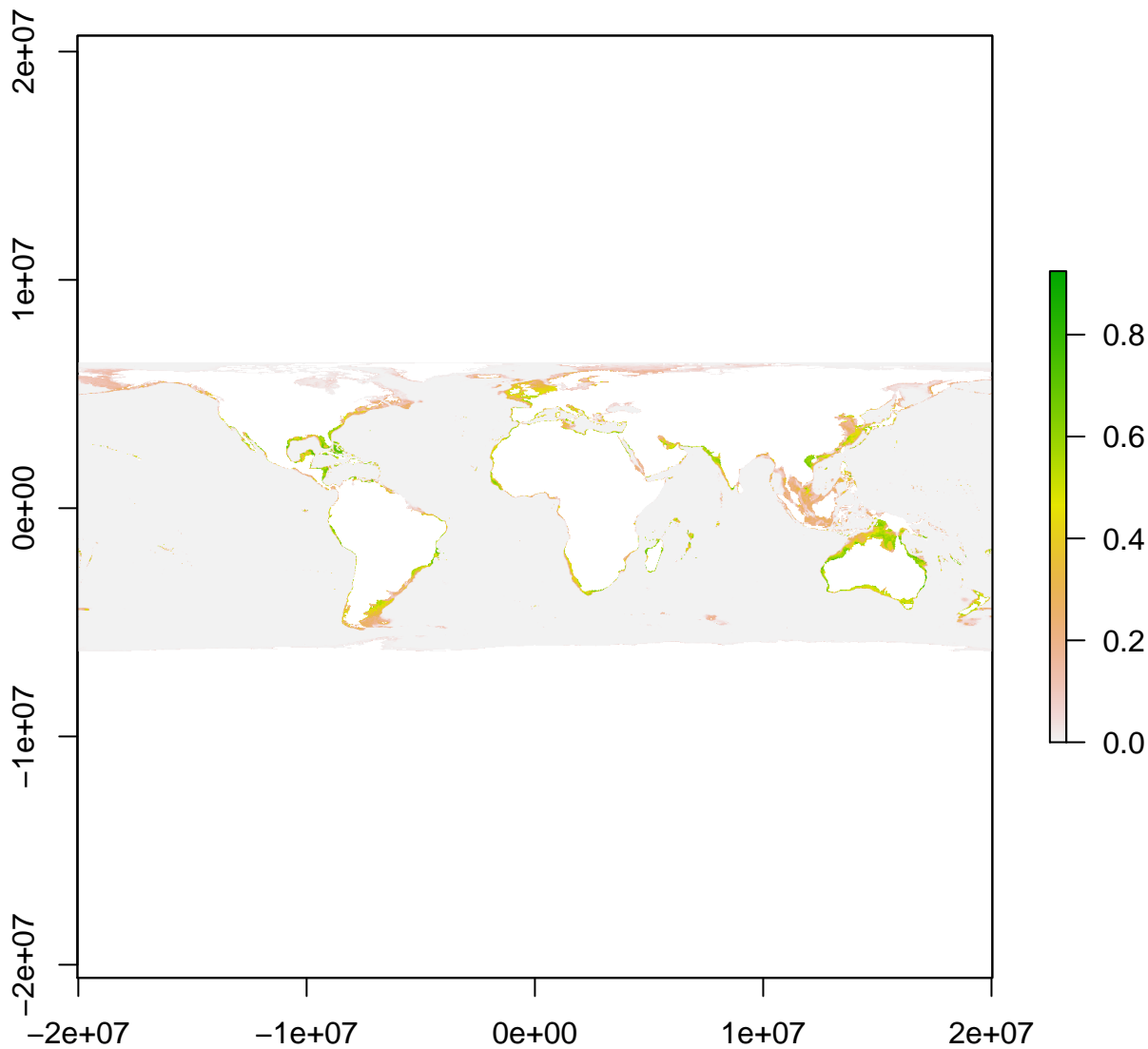

Supplement: Supplementary file 1 [file biology-11-01424-s001.zip › Maps/Ensemble/g_catenatum/2050_RCP6.0_ensembled.pdf]

# Gymnodinium\_catenatum\_2050\_RCP8.5

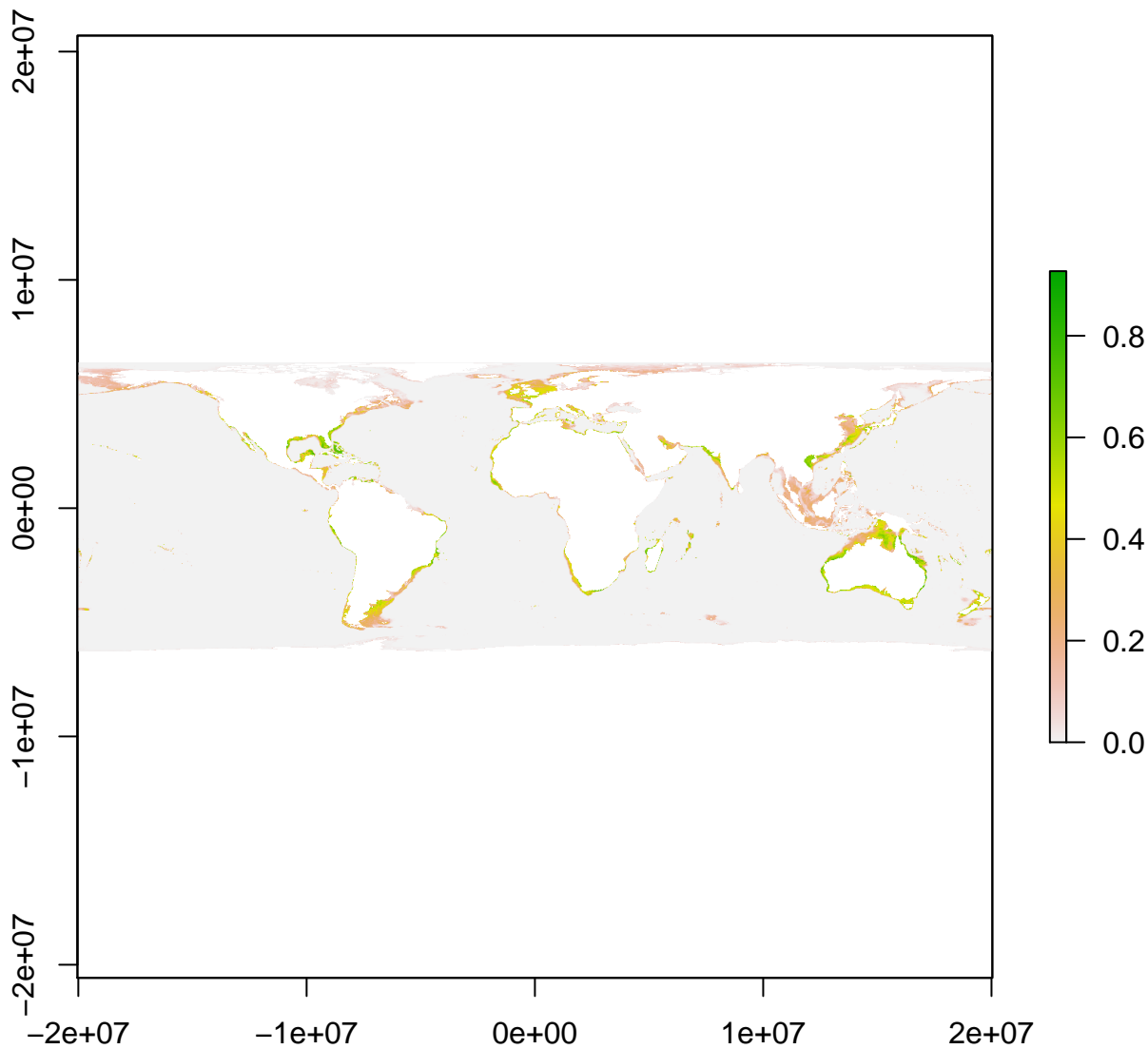

Supplement: Supplementary file 1 [file biology-11-01424-s001.zip › Maps/Ensemble/g_catenatum/2050_RCP8.5_ensembled.pdf]

# Gymnodinium\_catenatum\_2100\_RCP2.6

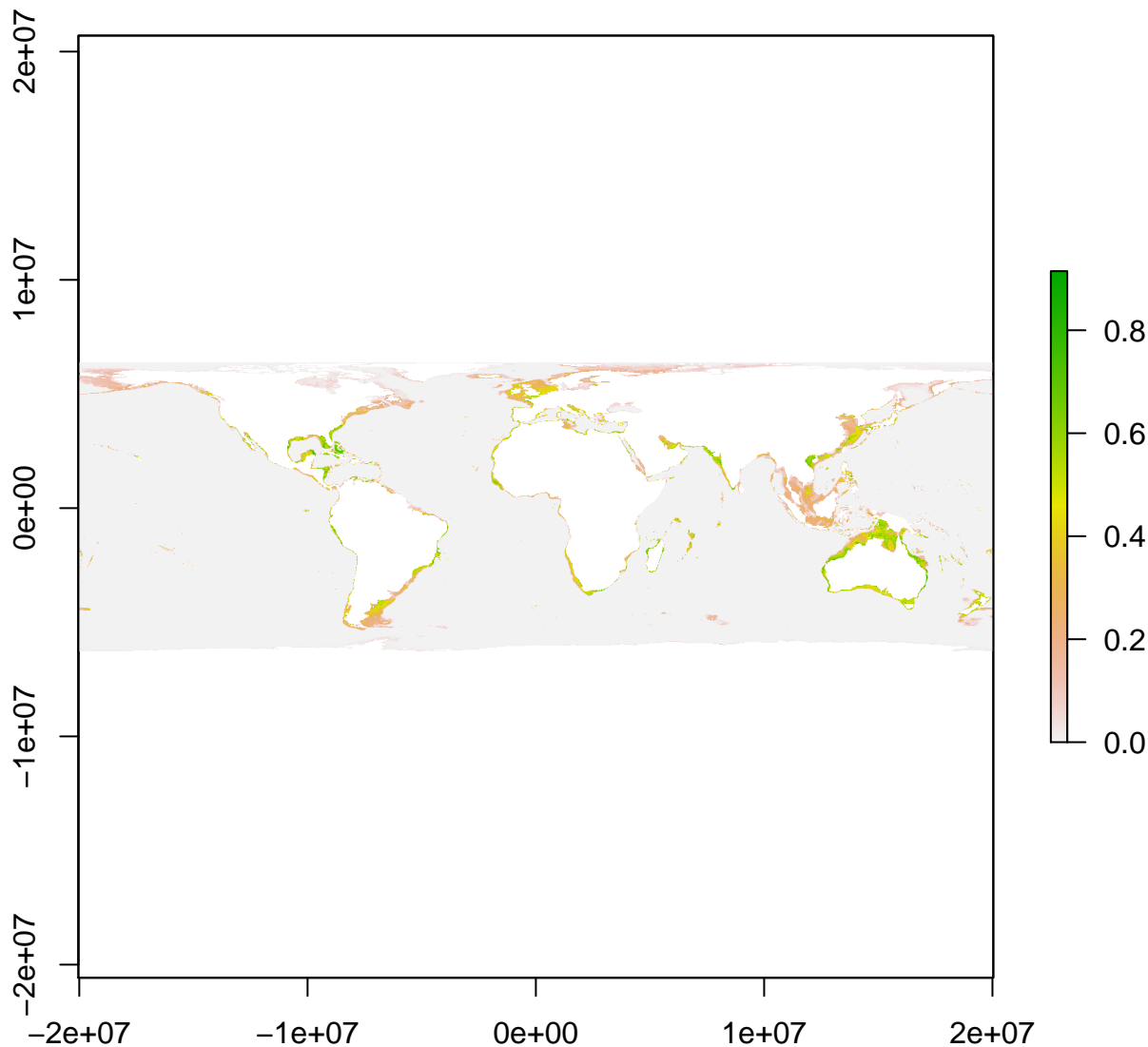

Supplement: Supplementary file 1 [file biology-11-01424-s001.zip › Maps/Ensemble/g_catenatum/2100_RCP2.6_ensembled.pdf]

# Gymnodinium\_catenatum\_2100\_RCP4.5

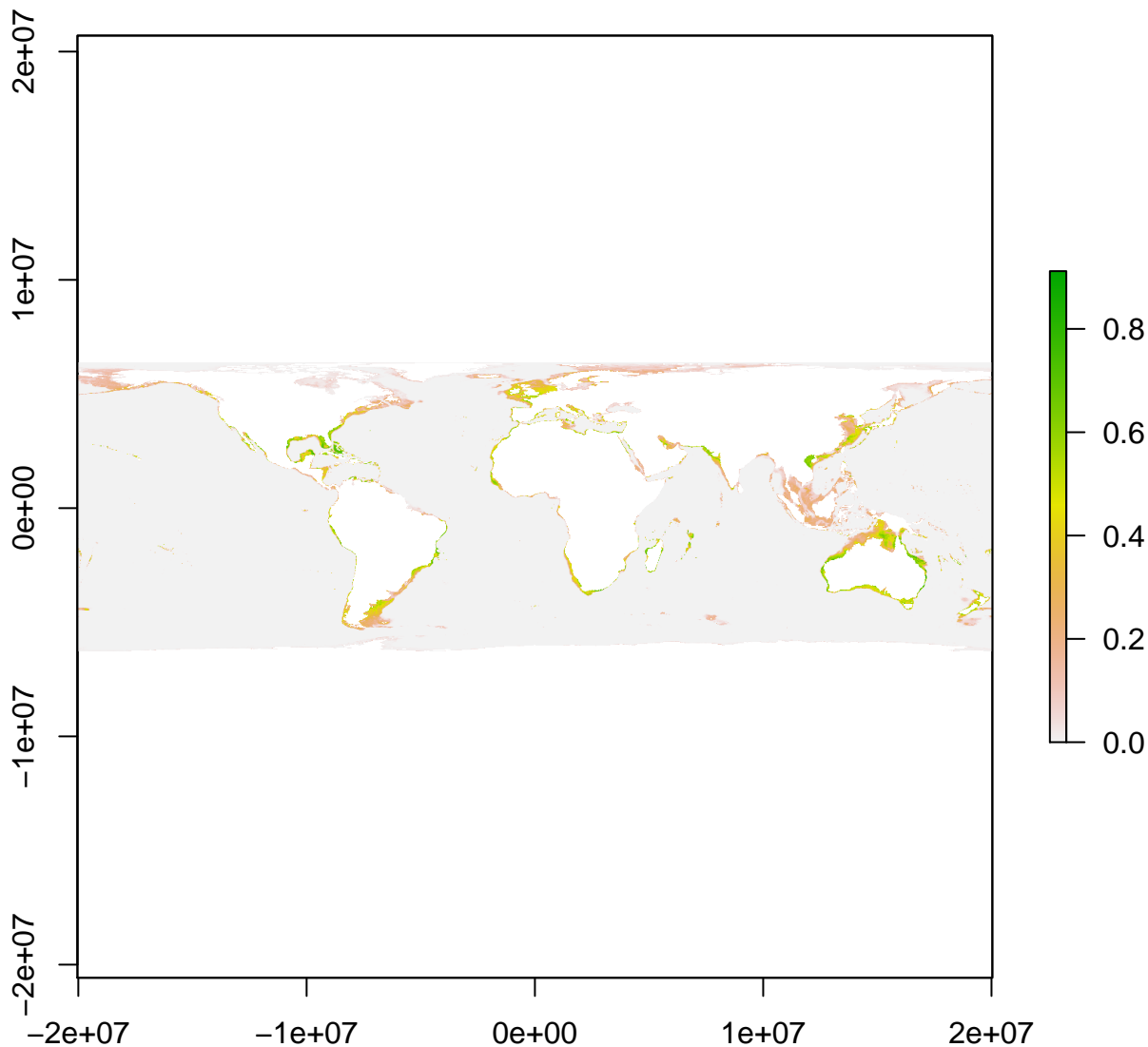

Supplement: Supplementary file 1 [file biology-11-01424-s001.zip › Maps/Ensemble/g_catenatum/2100_RCP4.5_ensembled.pdf]

# Gymnodinium\_catenatum\_2100\_RCP6.0

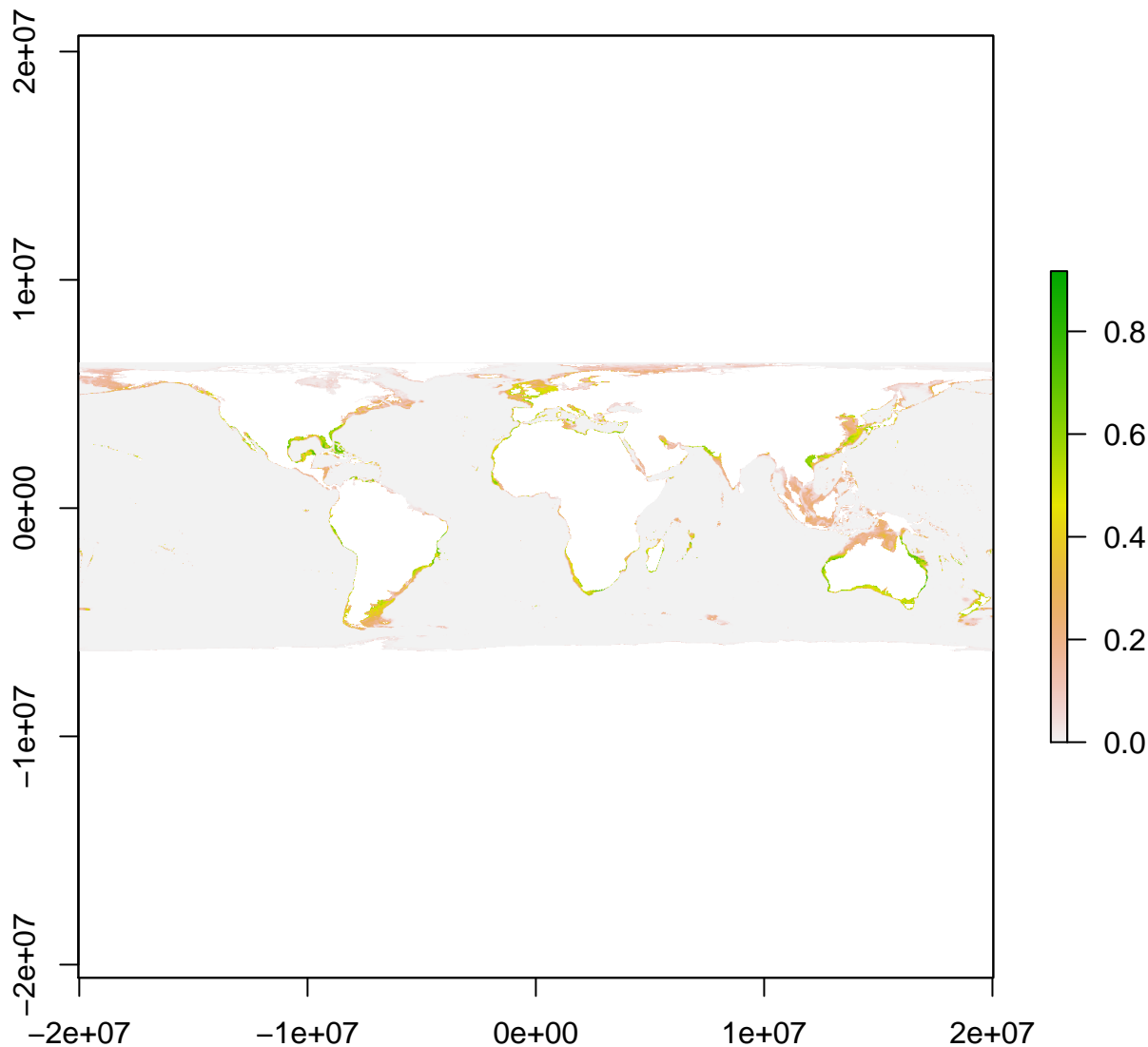

Supplement: Supplementary file 1 [file biology-11-01424-s001.zip › Maps/Ensemble/g_catenatum/2100_RCP6.0_ensembled.pdf]

# Gymnodinium\_catenatum\_2100\_RCP8.5

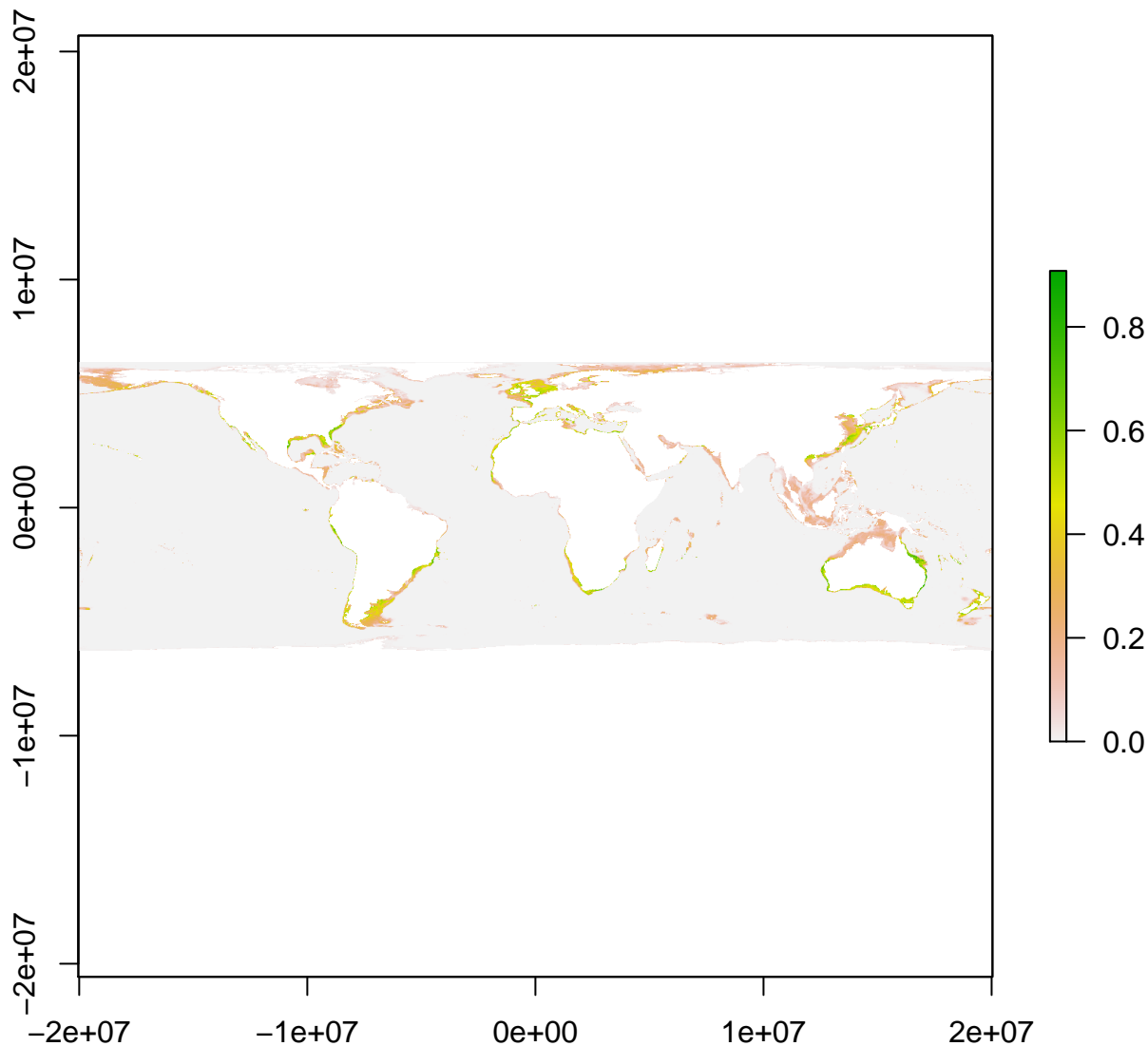

Supplement: Supplementary file 1 [file biology-11-01424-s001.zip › Maps/Ensemble/g_catenatum/2100_RCP8.5_ensembled.pdf]

# Gymnodinium\_catenatum\_present

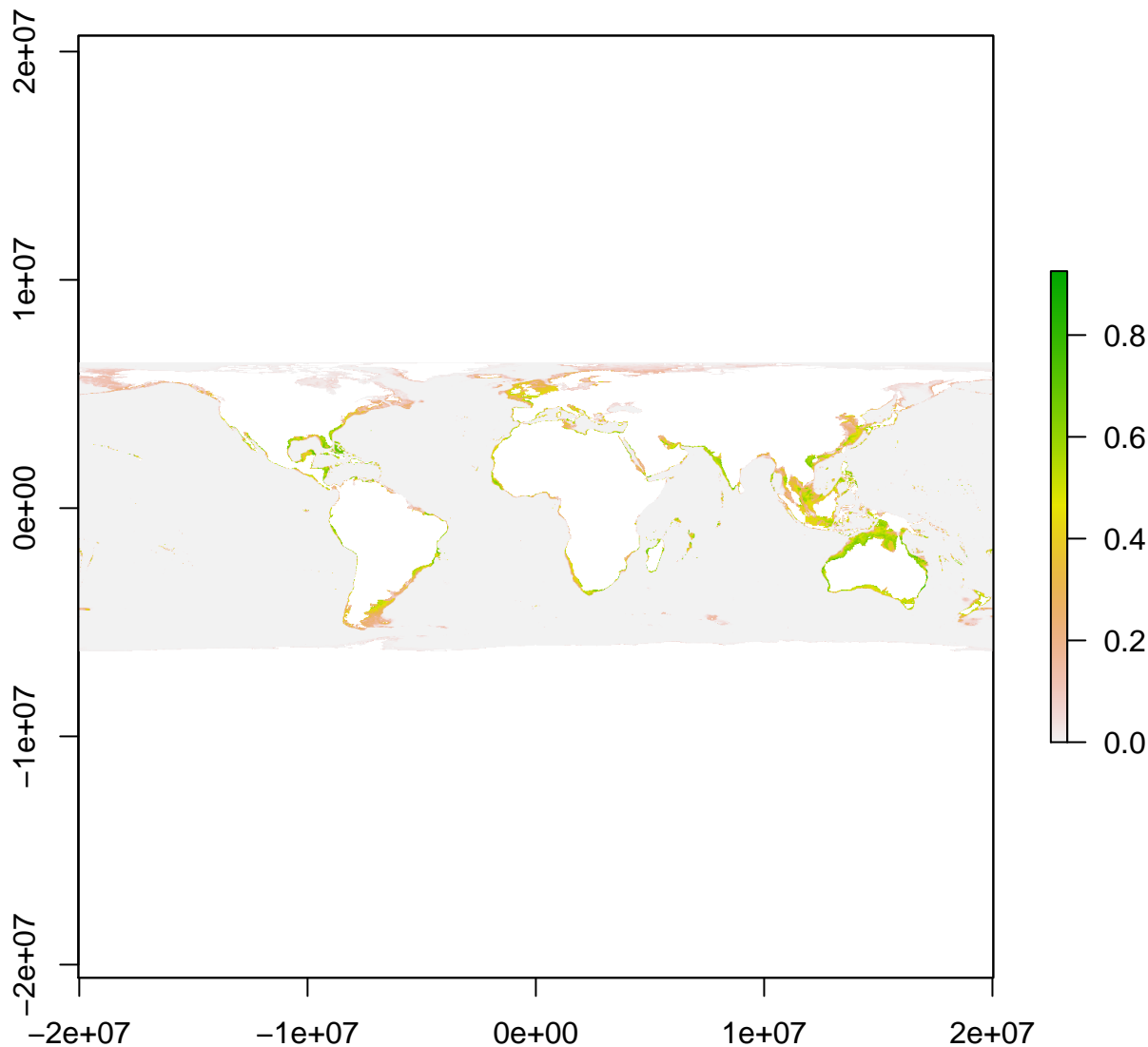

Supplement: Supplementary file 1 [file biology-11-01424-s001.zip › Maps/Ensemble/g_catenatum/present_ensembled.pdf]

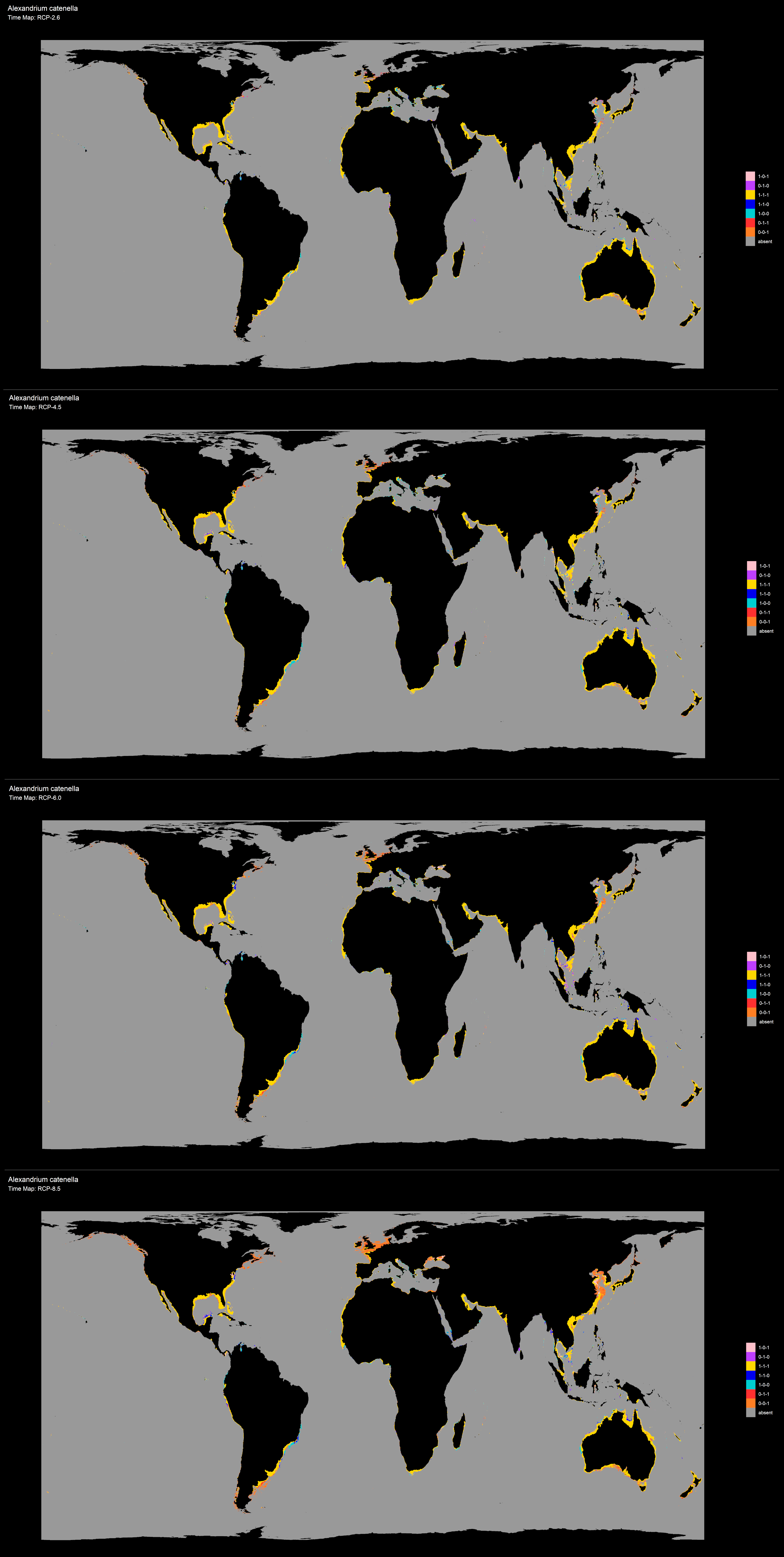

Supplement: Supplementary file 1 [file biology-11-01424-s001.zip › Maps/TimeMaps/a_catenella/Alexandrium_catenella_TimeMaps.png]

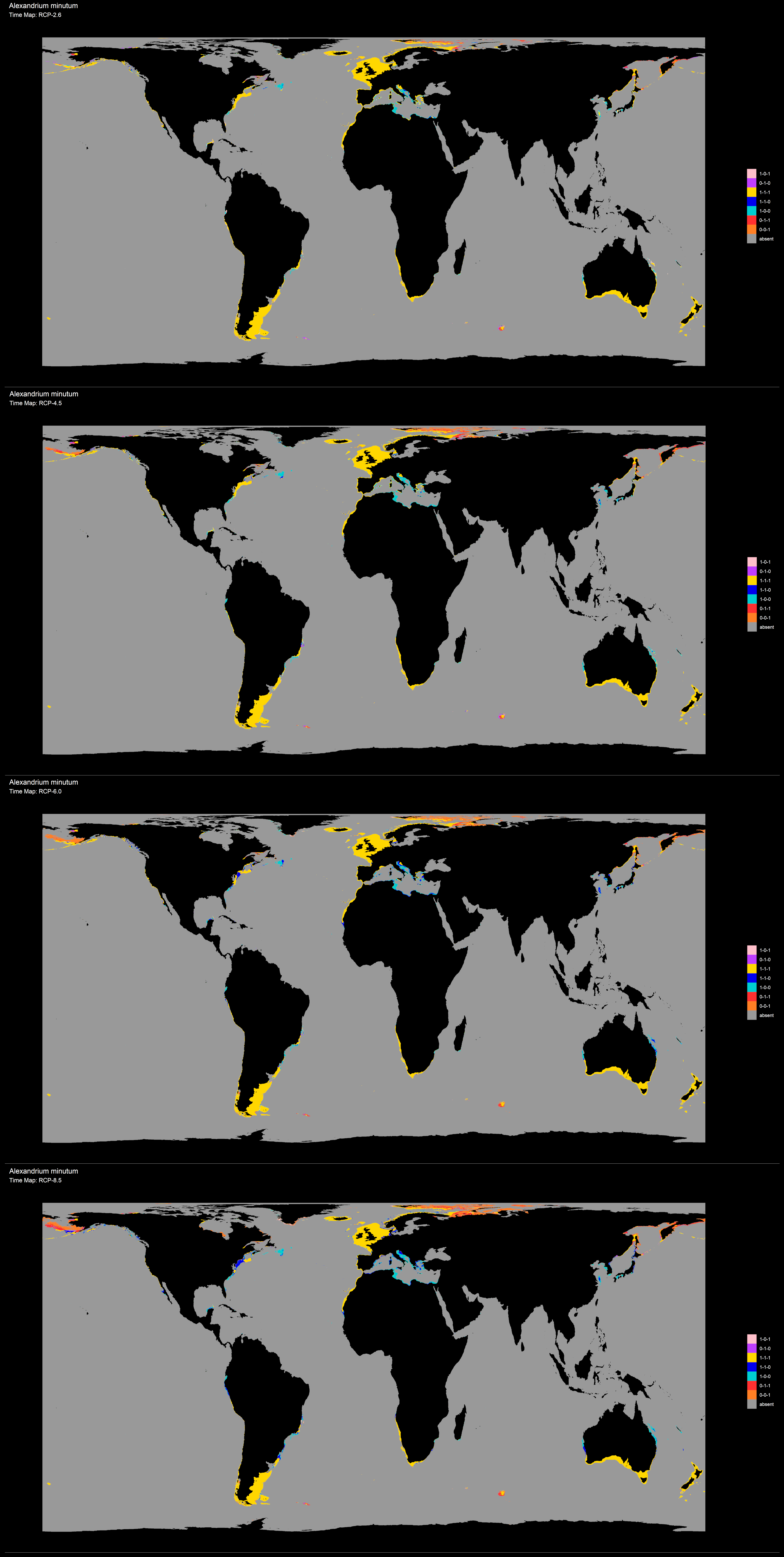

Supplement: Supplementary file 1 [file biology-11-01424-s001.zip › Maps/TimeMaps/a_minutum/Alexandrium_minutum_TimeMaps.png]

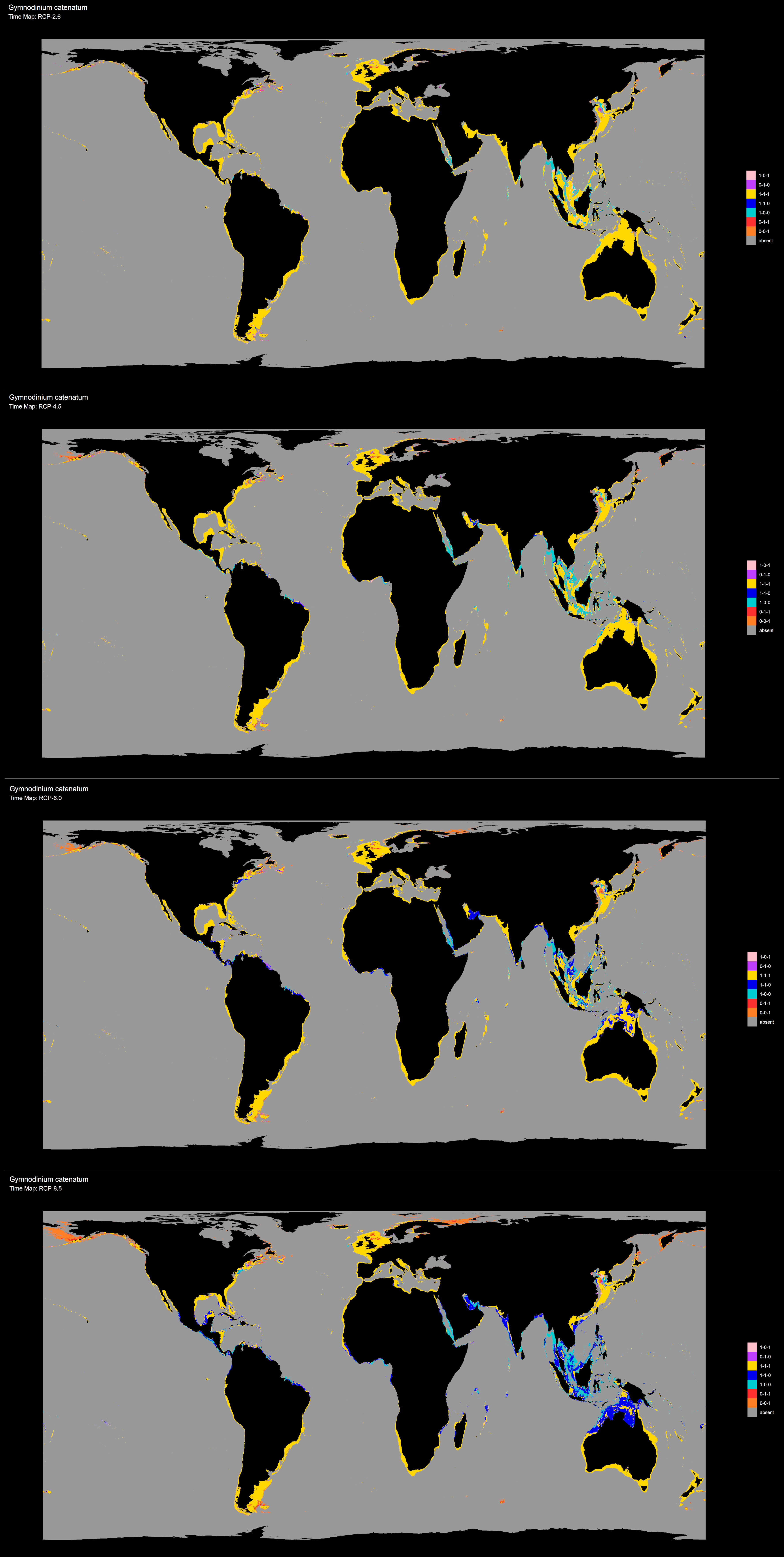

Supplement: Supplementary file 1 [file biology-11-01424-s001.zip › Maps/TimeMaps/g_catenatum/Gymnodinium_catenatum_TimeMaps.png]

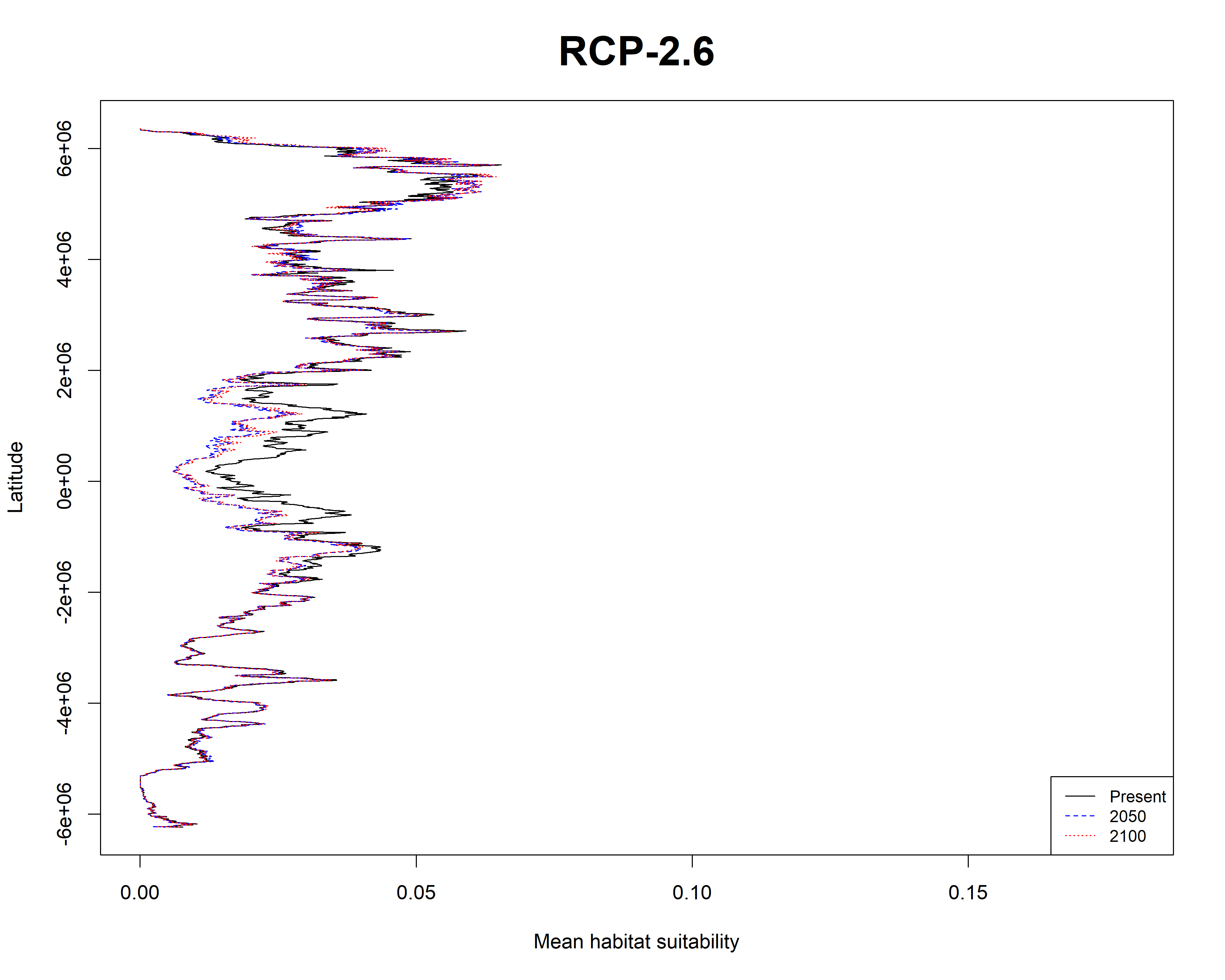

Supplement: Supplementary file 1 [file biology-11-01424-s001.zip › Post_analysis/mean_hab_suit_lat/lat_hab_catenatum26.tiff]

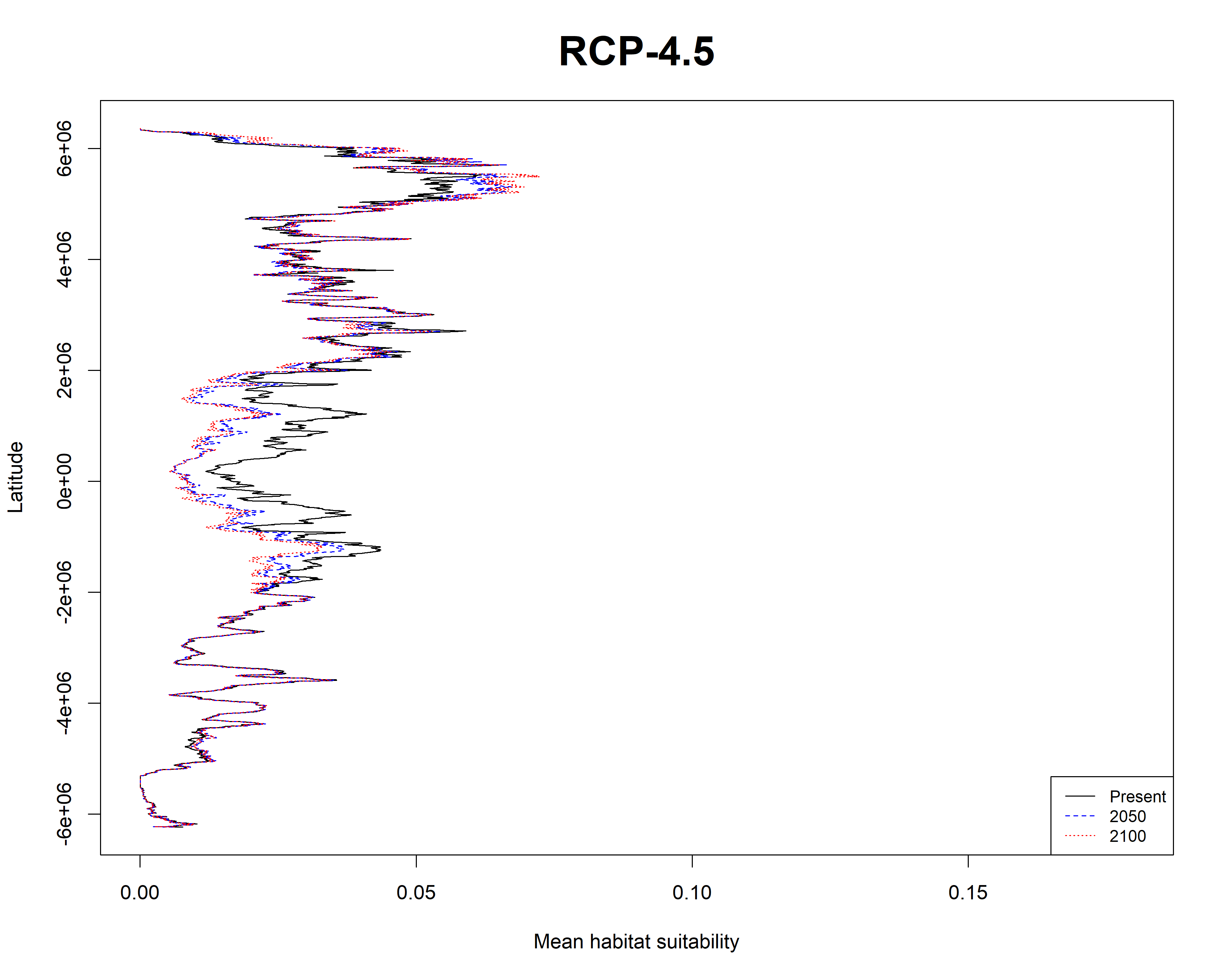

Supplement: Supplementary file 1 [file biology-11-01424-s001.zip › Post_analysis/mean_hab_suit_lat/lat_hab_catenatum45.tiff]

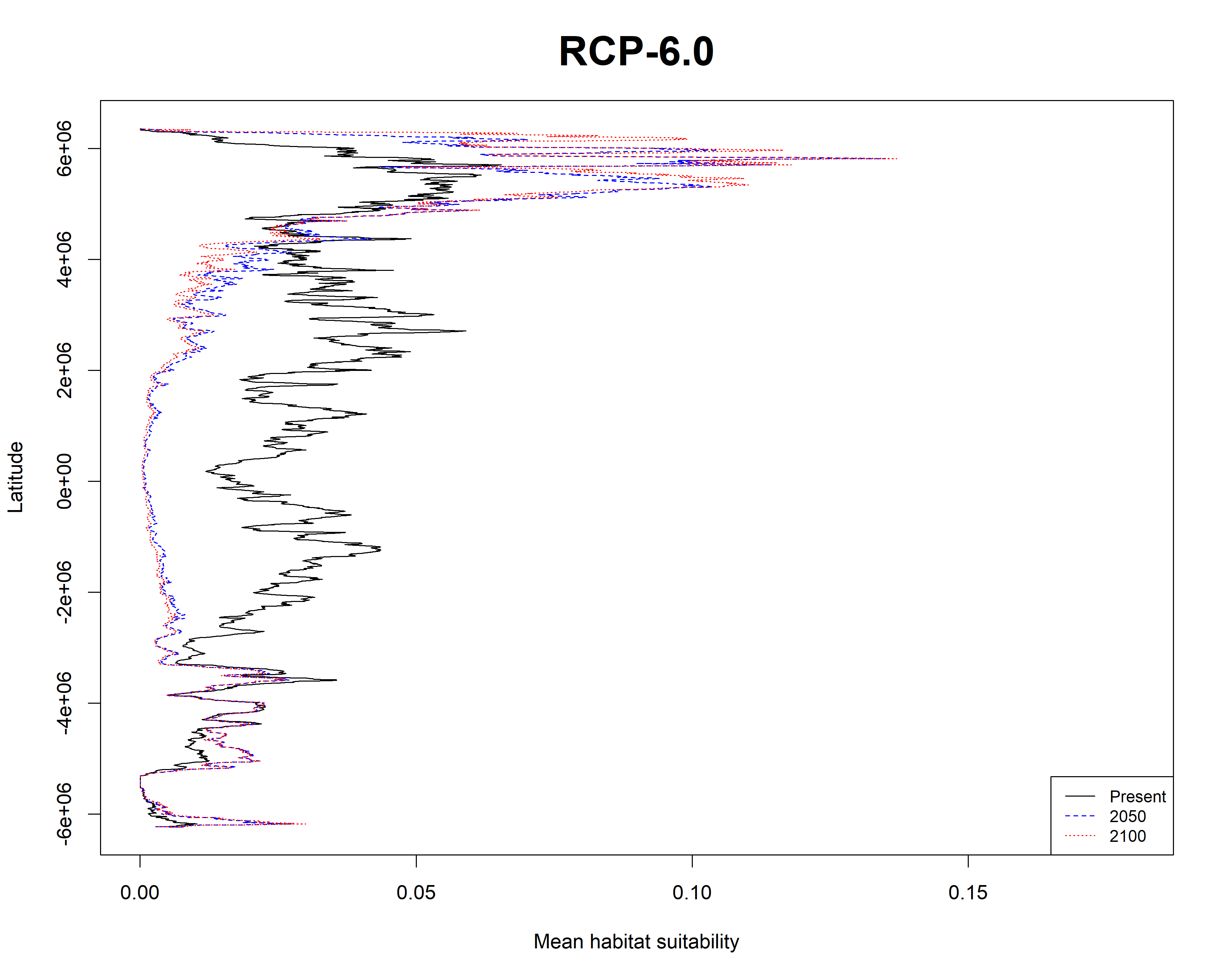

Supplement: Supplementary file 1 [file biology-11-01424-s001.zip › Post_analysis/mean_hab_suit_lat/lat_hab_catenatum60.tiff]

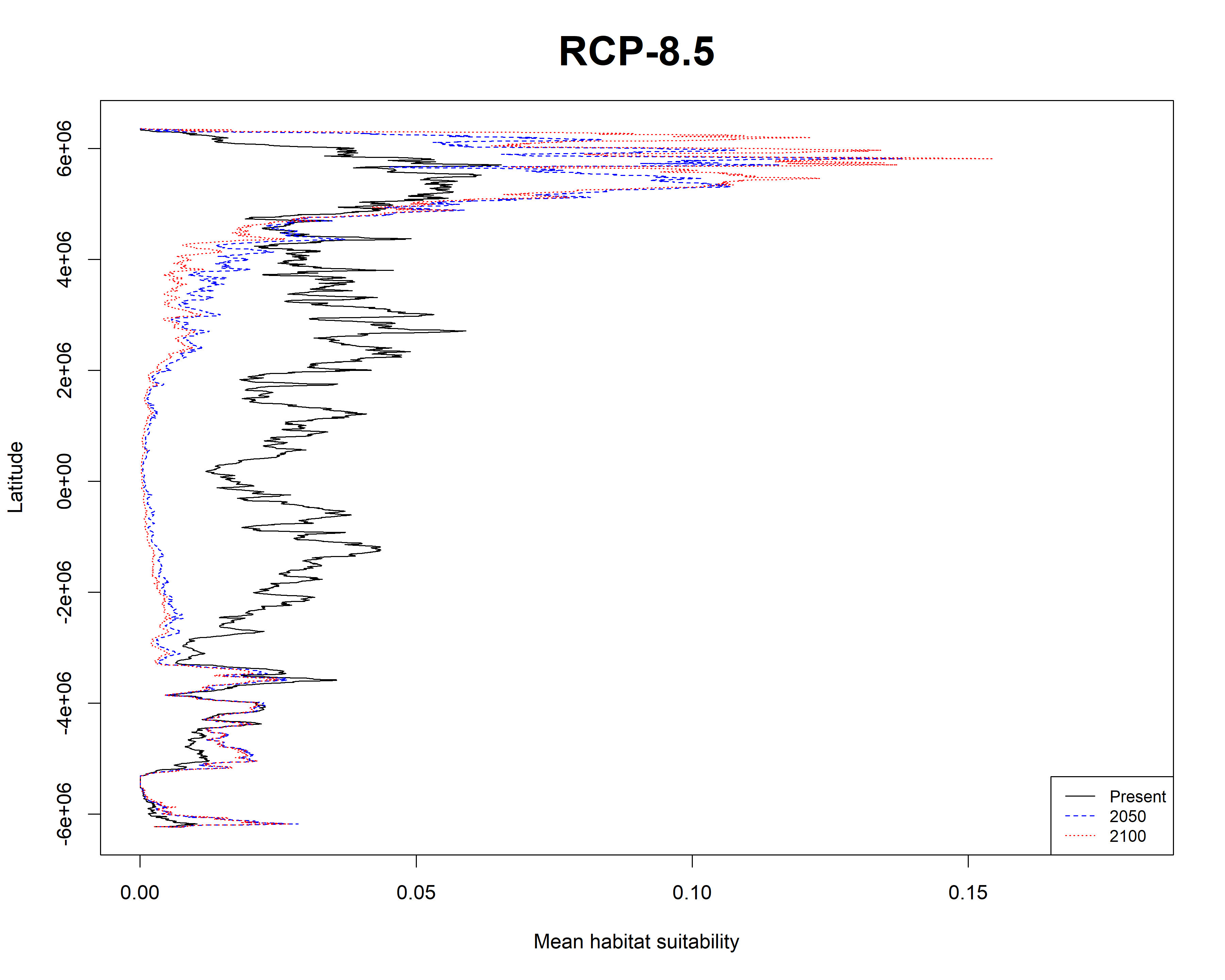

Supplement: Supplementary file 1 [file biology-11-01424-s001.zip › Post_analysis/mean_hab_suit_lat/lat_hab_catenatum85.tiff]

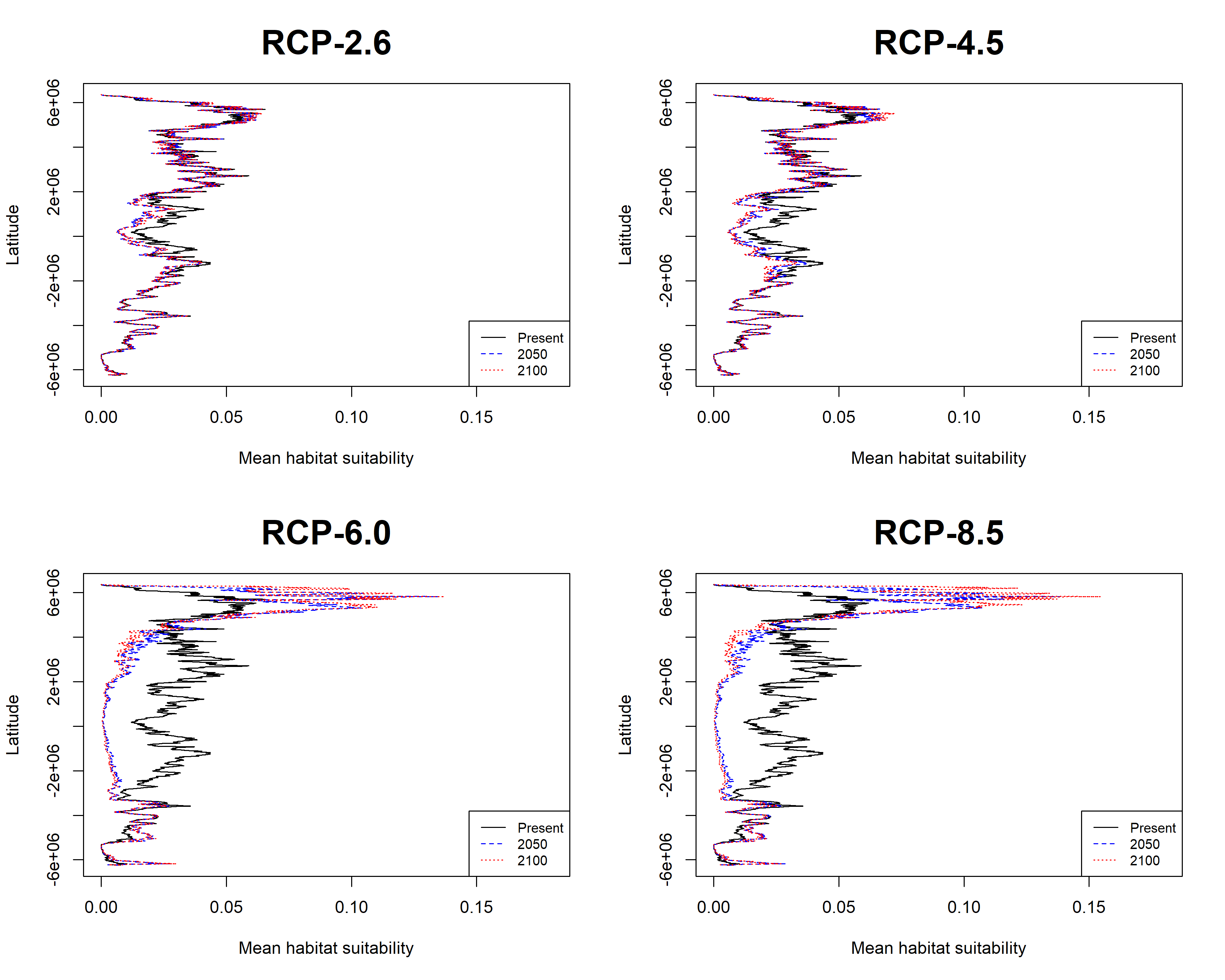

Supplement: Supplementary file 1 [file biology-11-01424-s001.zip › Post_analysis/mean_hab_suit_lat/lat_hab_catenatum_full.tiff]

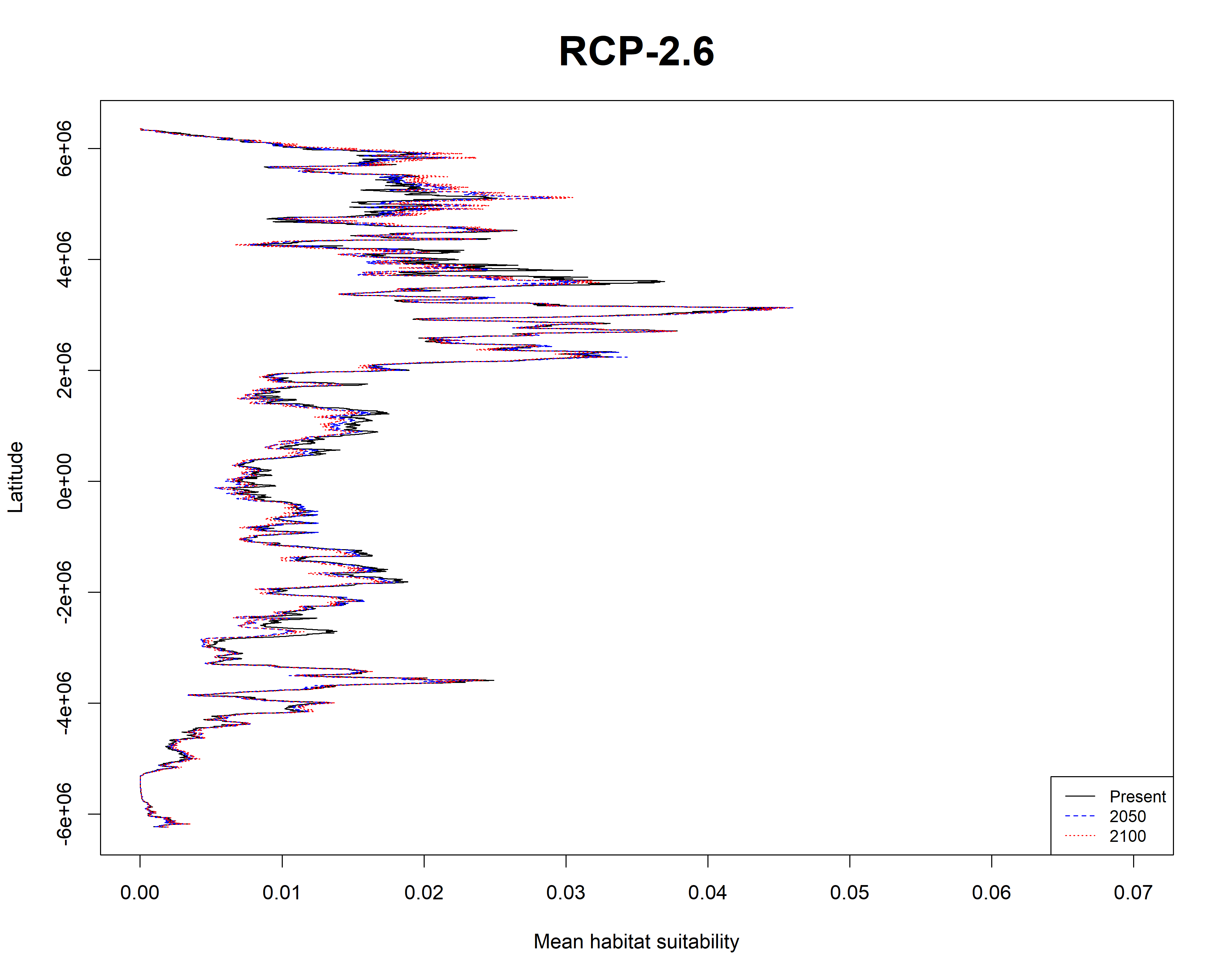

Supplement: Supplementary file 1 [file biology-11-01424-s001.zip › Post_analysis/mean_hab_suit_lat/lat_hab_catenella26.tiff]

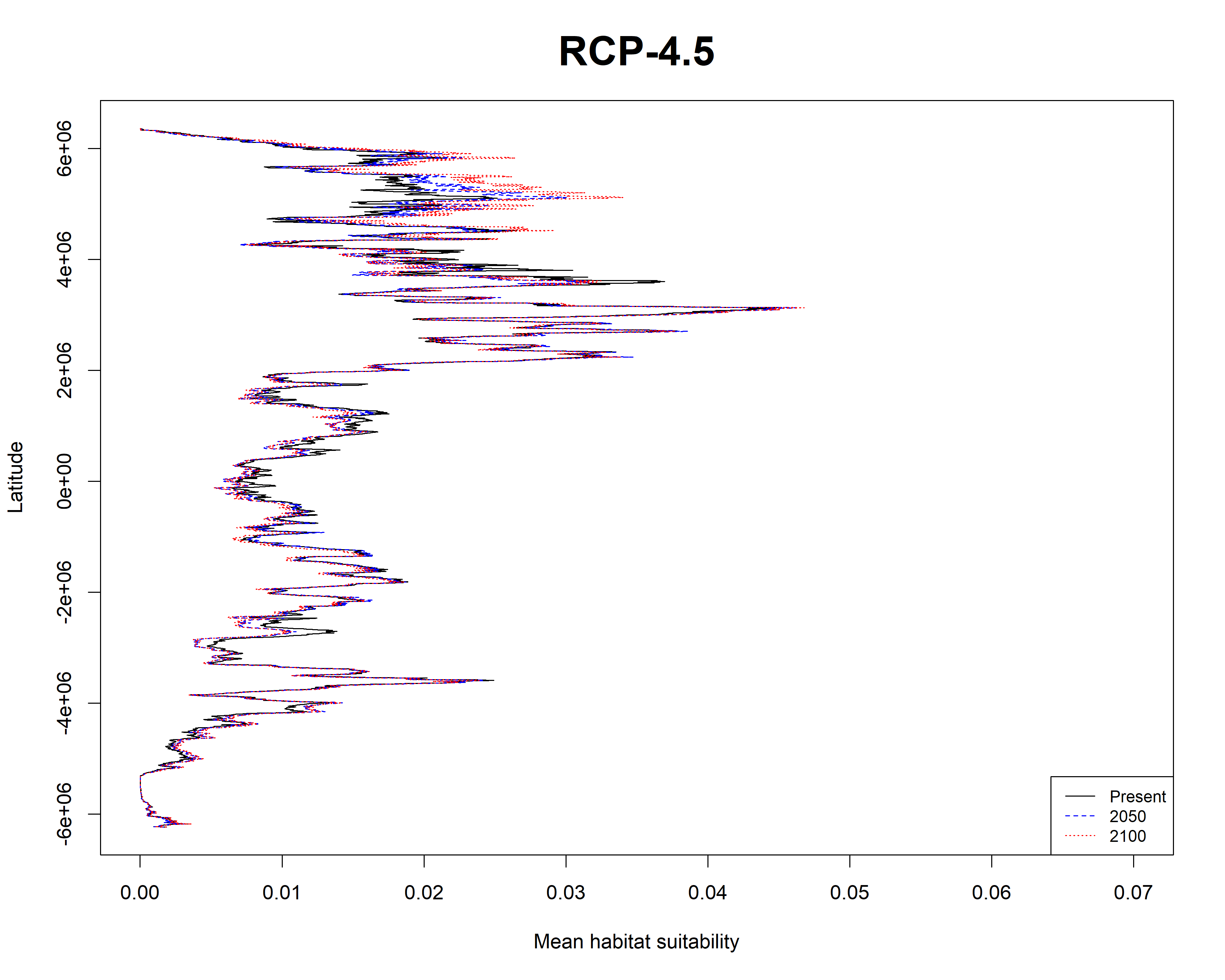

Supplement: Supplementary file 1 [file biology-11-01424-s001.zip › Post_analysis/mean_hab_suit_lat/lat_hab_catenella45.tiff]

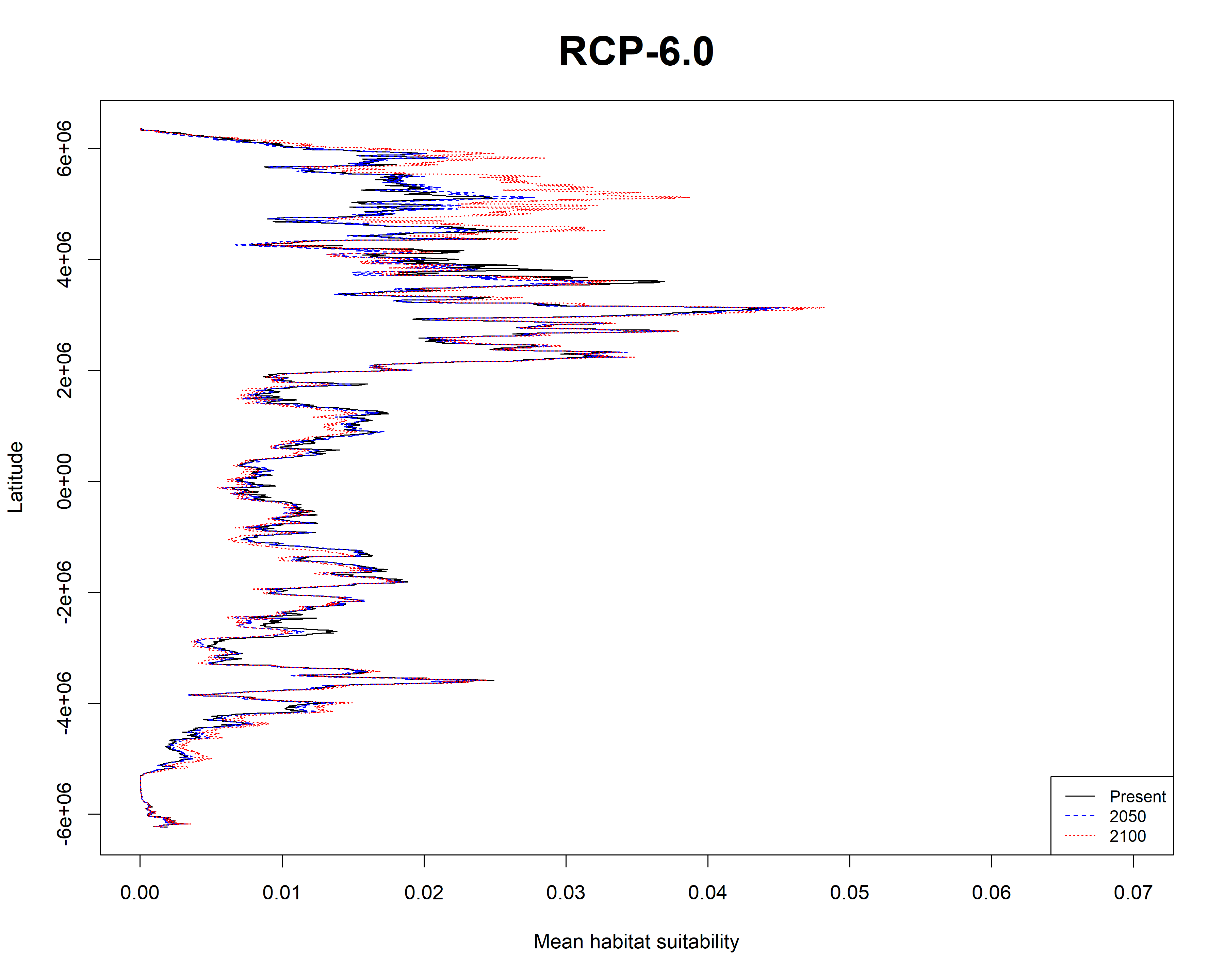

Supplement: Supplementary file 1 [file biology-11-01424-s001.zip › Post_analysis/mean_hab_suit_lat/lat_hab_catenella60.tiff]

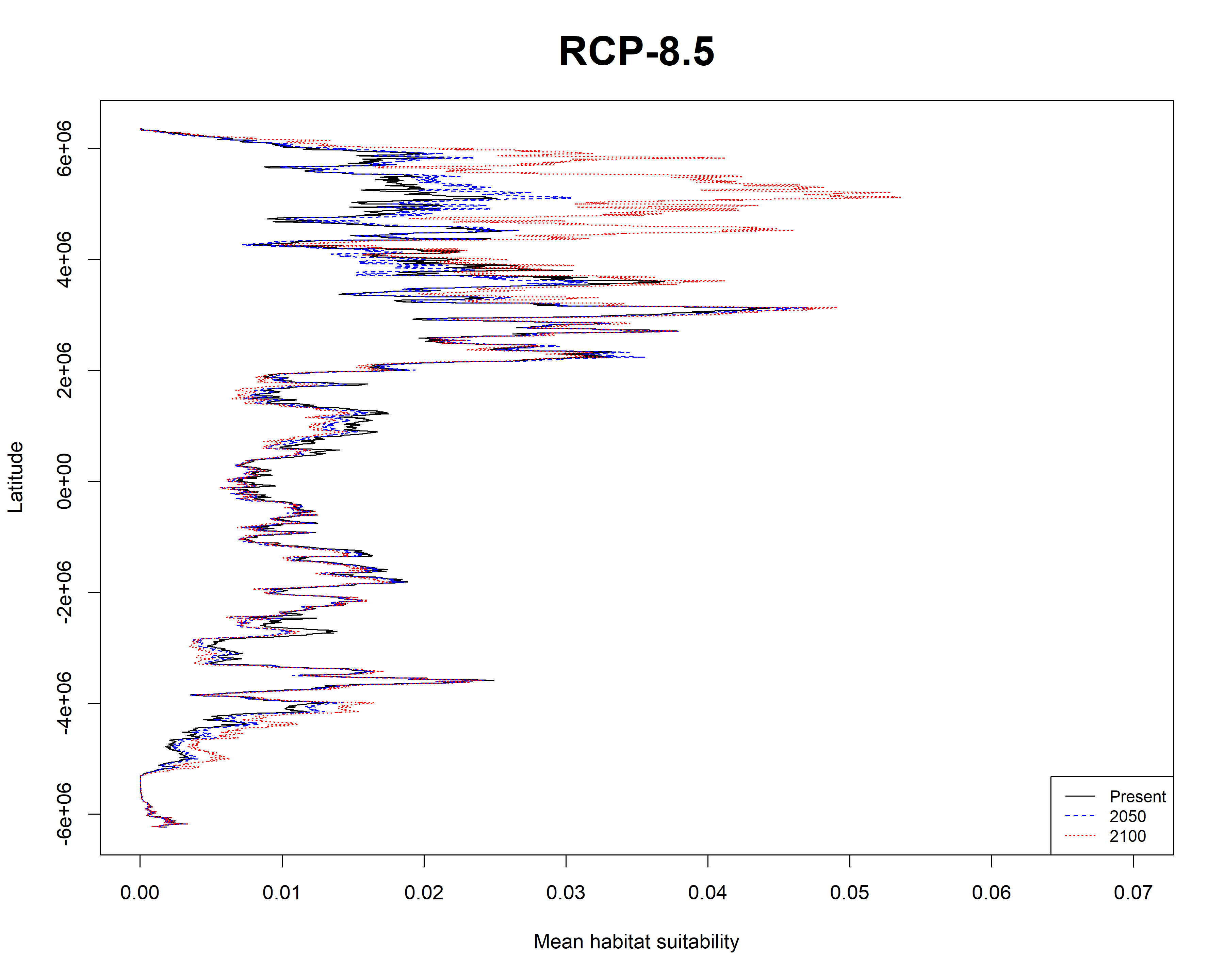

Supplement: Supplementary file 1 [file biology-11-01424-s001.zip › Post_analysis/mean_hab_suit_lat/lat_hab_catenella85.tiff]

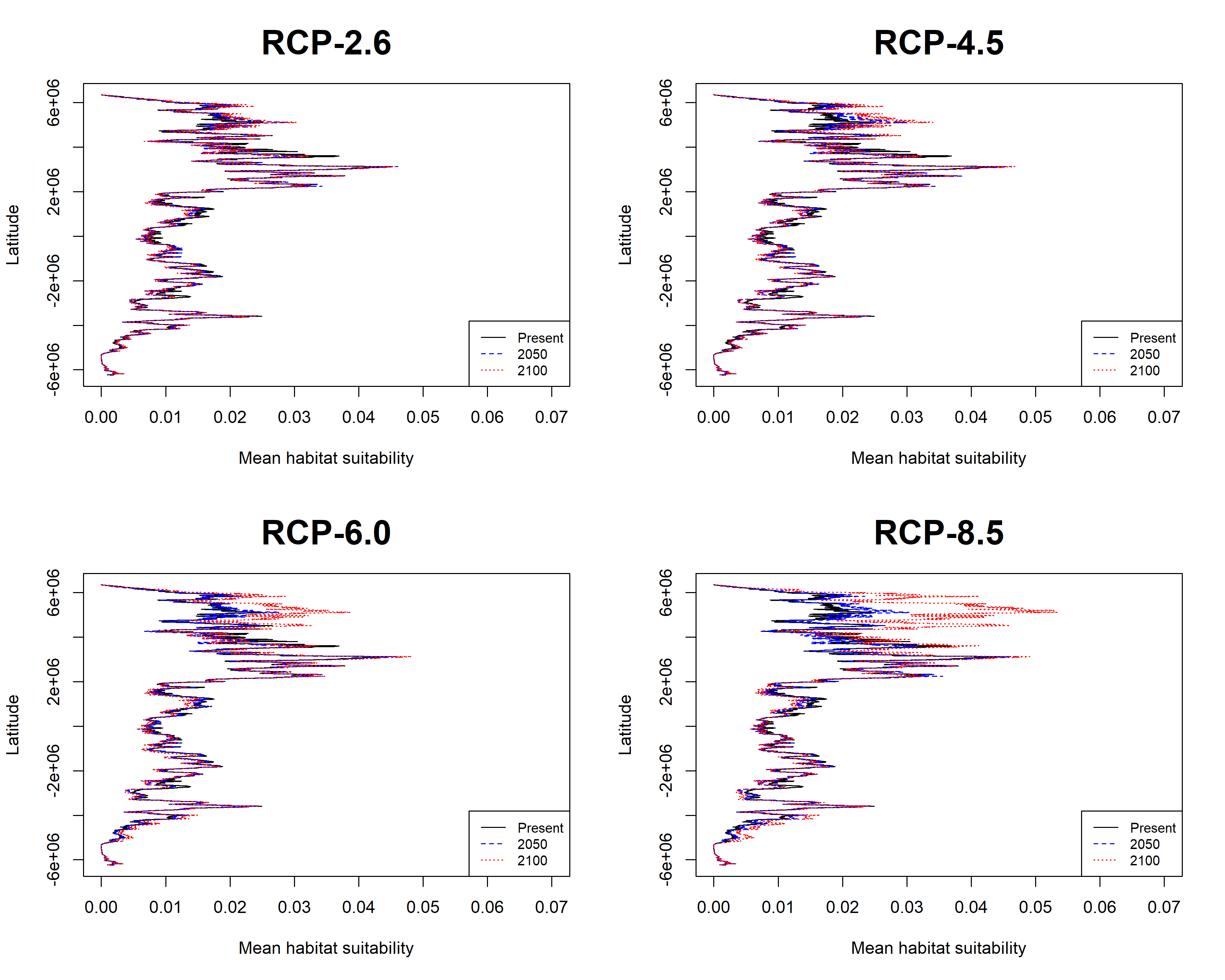

Supplement: Supplementary file 1 [file biology-11-01424-s001.zip › Post_analysis/mean_hab_suit_lat/lat_hab_catenella_full.tiff]

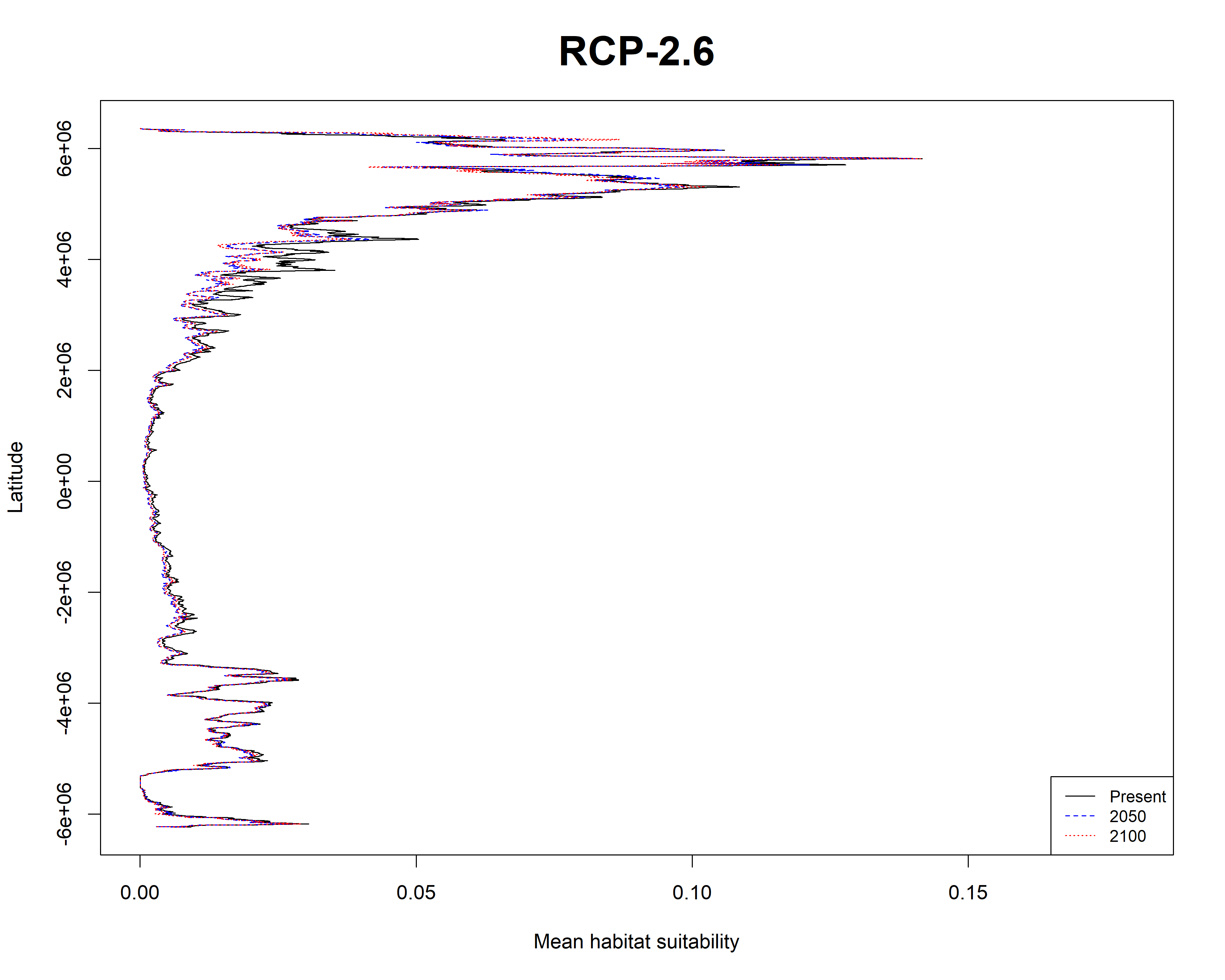

Supplement: Supplementary file 1 [file biology-11-01424-s001.zip › Post_analysis/mean_hab_suit_lat/lat_hab_minutum26.tiff]

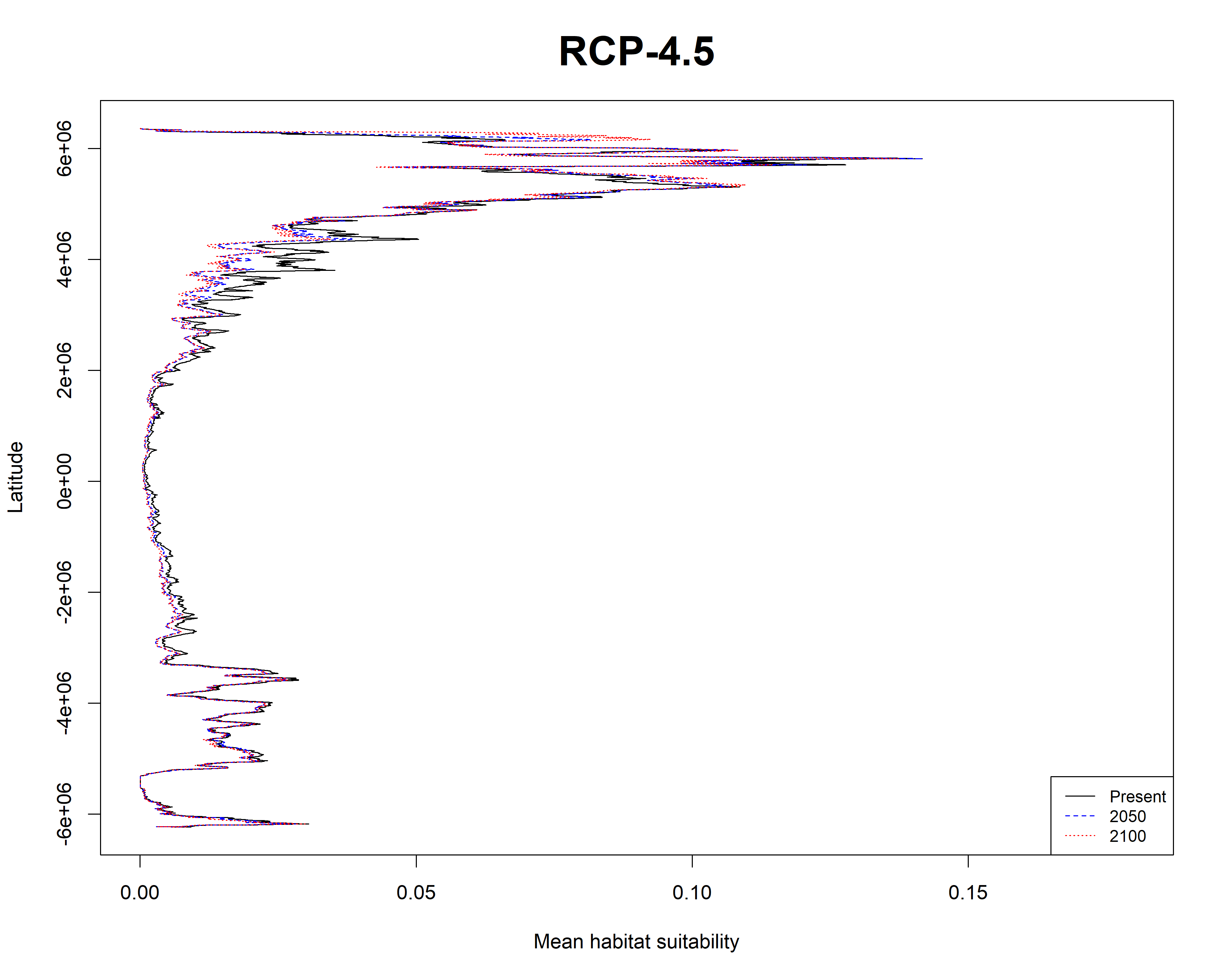

Supplement: Supplementary file 1 [file biology-11-01424-s001.zip › Post_analysis/mean_hab_suit_lat/lat_hab_minutum45.tiff]

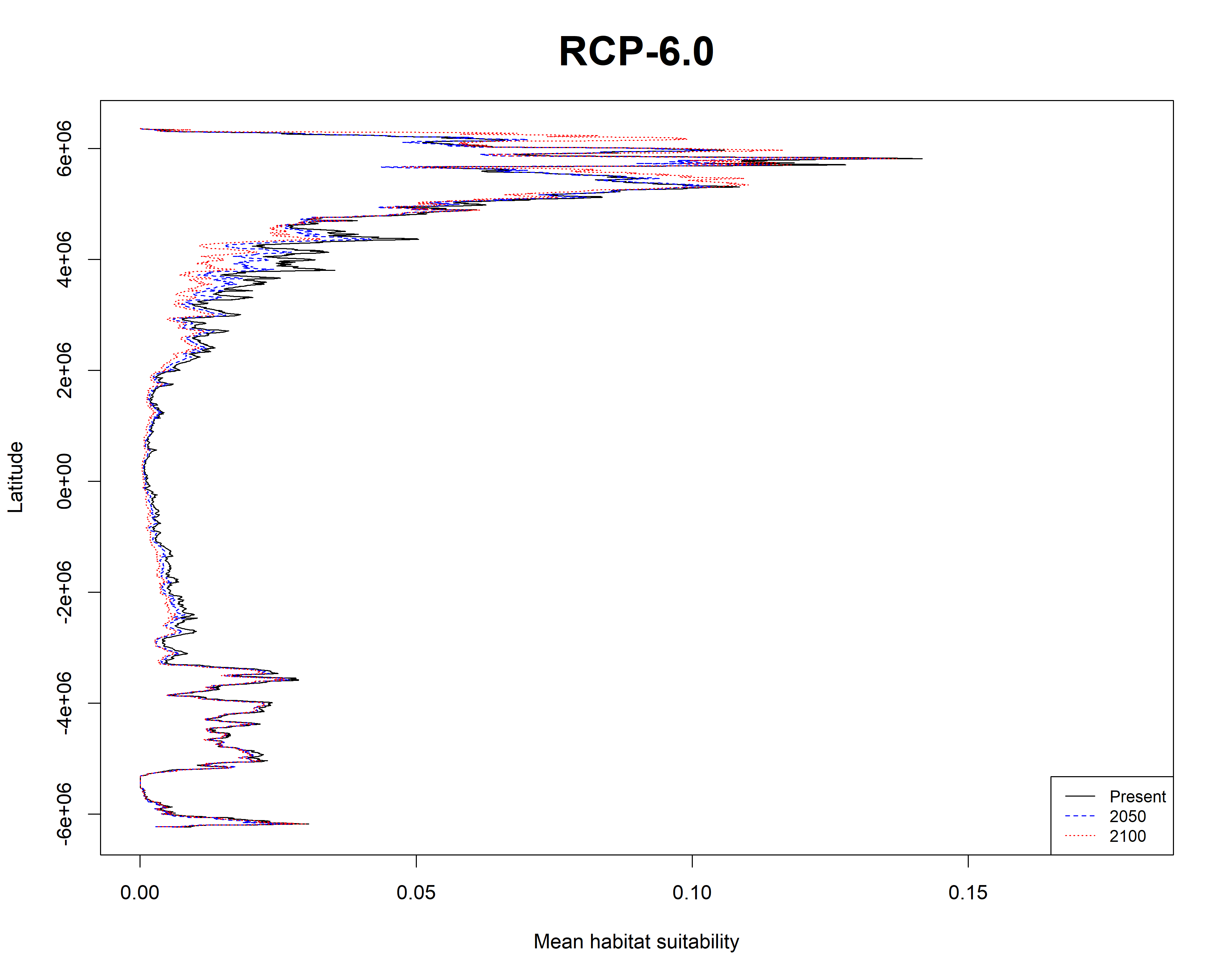

Supplement: Supplementary file 1 [file biology-11-01424-s001.zip › Post_analysis/mean_hab_suit_lat/lat_hab_minutum60.tiff]

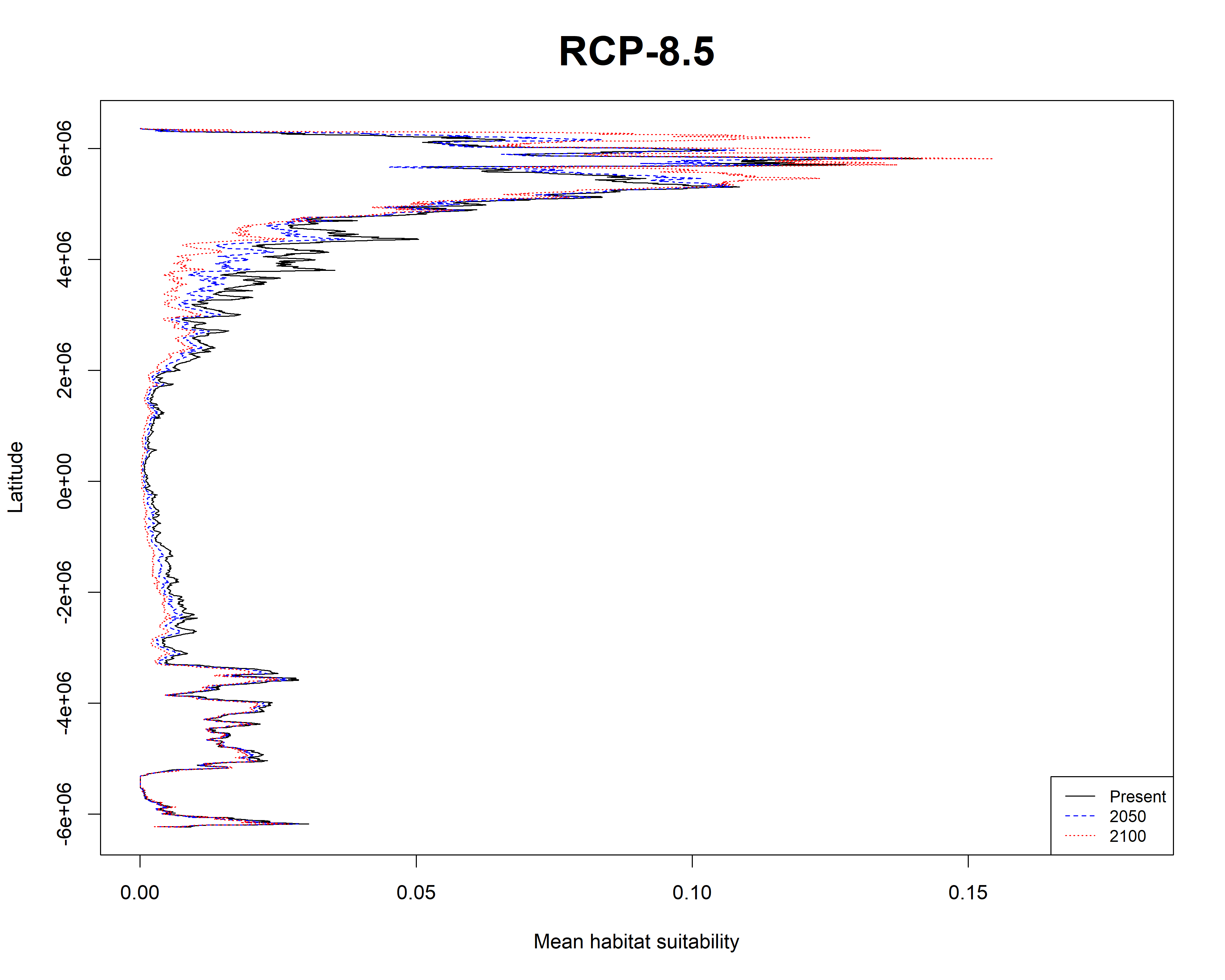

Supplement: Supplementary file 1 [file biology-11-01424-s001.zip › Post_analysis/mean_hab_suit_lat/lat_hab_minutum85.tiff]

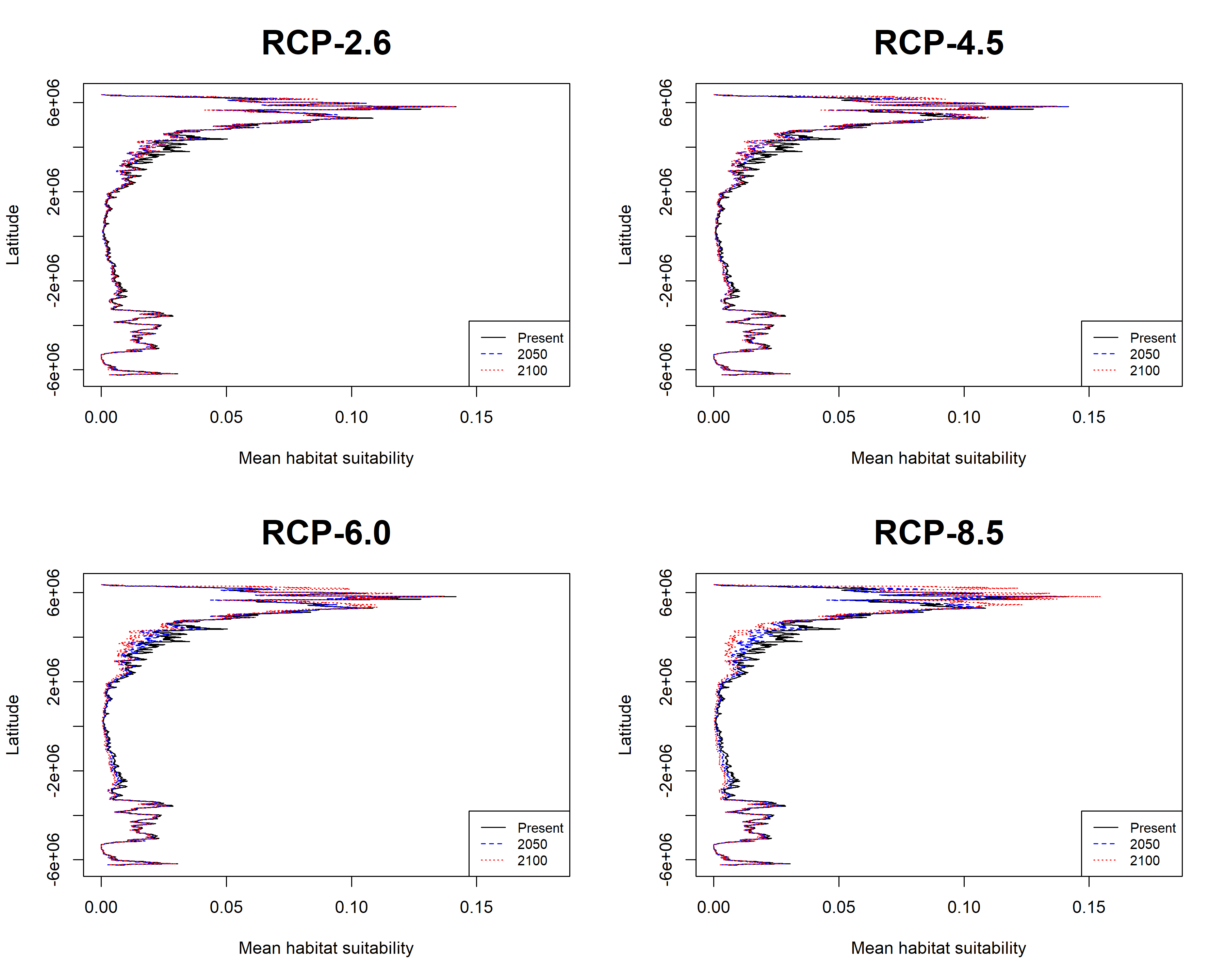

Supplement: Supplementary file 1 [file biology-11-01424-s001.zip › Post_analysis/mean_hab_suit_lat/lat_hab_minutum_full.tiff]
